# *N*‐Methyl‐Benzothiazolium Salts as Carbon Lewis Acids for Si−H σ‐Bond Activation and Catalytic (De)hydrosilylation

**DOI:** 10.1002/chem.201604613

**Published:** 2016-11-22

**Authors:** Valerio Fasano, James E. Radcliffe, Liam D. Curless, Michael J. Ingleson

**Affiliations:** ^1^ School of ChemistryUniversity of ManchesterManchesterM13 9PLUK

**Keywords:** benzothiazolium salts, frustrated Lewis pairs, hydrosilylation, Lewis acids, organocatalysis

## Abstract

*N*−Me‐Benzothiazolium salts are introduced as a new family of Lewis acids able to activate Si−H σ bonds. These carbon‐centred Lewis acids were demonstrated to have comparable Lewis acidity towards hydride as found for the triarylboranes widely used in Si−H σ‐bond activation. However, they display low Lewis acidity towards hard Lewis bases such as Et_3_PO and H_2_O in contrast to triarylboranes. The *N*−Me‐benzothiazolium salts are effective catalysts for a range of hydrosilylation and dehydrosilylation reactions. Judicious selection of the C2 aryl substituent in these cations enables tuning of the steric and electronic environment around the electrophilic centre to generate more active catalysts. Finally, related benzoxazolium and benzimidazolium salts were found also to be active for Si−H bond activation and as catalysts for the hydrosilylation of imines.

## Introduction

**Scheme 1:**
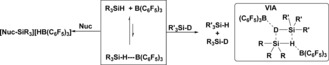
Silane activation with B(C_6_F_5_)_3_.

The use of hydridophilic main‐group Lewis acids, particularly B(C_6_F_5_)_3_, in Si−H bond activation,^1^ and more broadly in “frustrated Lewis pair” (FLP) chemistry,^2^ has generated significant recent breakthroughs.^3^ This includes their use as versatile catalysts for dehydrosilylation and hydrosilylation reactions.^4^ Generally, the Lewis acids employed are tri(fluoroaryl)boranes which have sufficient Lewis acidity towards hydride to heterolytically cleave Si−H bonds (in combination with an appropriate Lewis base) and generate borohydrides that are able subsequently to reduce electrophilic substrates. Mechanistic studies revealed that Si−H heterolysis is an S_N_2 type process proceeding via a “partially” activated Si−H bond, which can be viewed as a “Si‐H‐B” 3c–2e interaction,^5^, ^6^ with one example recently crystallographically characterised.^7^ In the absence of an appropriate nucleophile no silylium ions are formed from combining B(C_6_F_5_)_3_ and R_3_SiH, although silane H/D scrambling still proceeds via a four‐membered transition state (inset Scheme 1).^7^

In more recent studies, weaker boron Lewis acids, such as BPh_3_, also have been shown also to be effective in Si−H bond activation.^8^ Nevertheless, the high oxophilicity of boron Lewis acids requires rigorously dried conditions (or an excess of hydride)^9^ and leads to substrate scope limitations.^10^ Consequently, the development of new Lewis acids that have low oxophilicity but retain sufficient Lewis acidity towards hydride to activate E−H (E=H or R_3_Si) bonds is desirable.^11^ Lewis acids in which carbon is the locus of electrophilic character have significant potential in this area as the higher electronegativity of carbon (relative to boron) results in a reduction in “hard” Lewis acidity.^12^ Trityl salts are amongst the most widely utilised carbon Lewis acids including in catalytic applications. However, these catalytic transformations generally proceed by activation of the substrate by coordination to the electrophilic carbon centre in trityl and not by an FLP‐type mechanism.^12^, ^13^ The use of trityl salts and other carbon Lewis acids in FLP chemistry, including the activation of H_2_, has significantly less precedent, although a limited number of examples have been recently reported.^14^

**Scheme 2:**
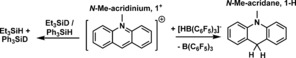
E−H bond activation using [*N*‐Me‐acridinium]^+^.

Carbocations including trityl, are well documented to irreversibly cleave Si−H bonds to form silylium cations and Ph_3_CH.^15^ In contrast, carbon Lewis acids that “partially” activate Si−H bonds (e.g., exhibit analogous reactivity to B(C_6_F_5_)_3_) are extremely rare to the best of our knowledge. One recent example from our group are *N*‐methyl‐acridinium salts which activate Si−H bonds (Scheme 2) as indicated by Si−H/Si−D scrambling experiments but no silylium cations are observed in solution.^16^ However, the Lewis acidity of the *N*−Me‐acridinium cation (**1** ^+^) towards hydride is greater than that of B(C_6_F_5_)_3._ 17 This makes the conjugate organic hydride, *N*‐Me‐acridane (**1‐H**), formed for example on Si‐H heterolysis by **1** ^+^ and an appropriate nucleophile, a poor reductant. This fact combined with the propensity of *N*−Me‐acridinium salts to initiate photoactivated radical reactivity^18^ led us to search for other carbon Lewis acids able to “partially” activate Si−H bonds but that are weaker Lewis acids towards hydride than **1** ^+^.

**Scheme 3:**
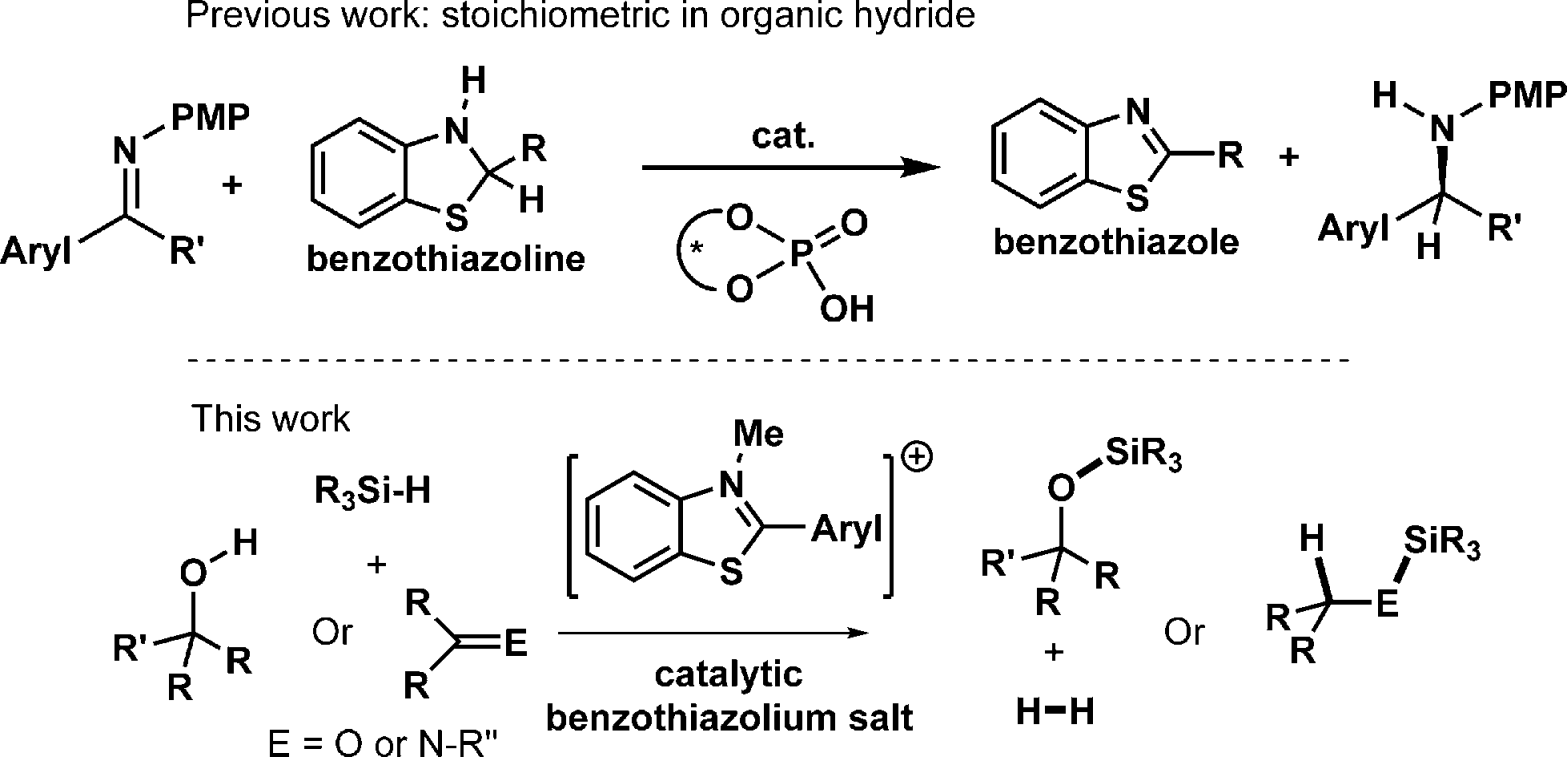
Top, Brønsted acid catalyzed transfer hydrogenation of imines using stoichiometric benzothiazoline. Bottom, benzothiazolium cation catalyzed hydrosilylation and dehydrosilylation using stoichiometric silane.

Previous work has shown that C2 substituted benzothiazolines are highly effective organic hydrides for the reduction of imines catalyzed by phosphoric acids (Scheme 3, top).^19^ Based on this precedence, we targeted the oxidised form, the benzothiazole, as a potential carbon Lewis acid after methylation at nitrogen to increase the electrophilicity at the C2 position. *N*‐Me‐2‐R‐benzothiazolium cations are attractive Lewis acids as they are simple to make using established routes and can be readily fine‐tuned e.g., by altering the C2 substituent.^19^ Furthermore, they represent a hitherto underexplored class of Lewis acid in FLP‐type catalysis, namely a Lewis acid based on an iminium cation, a moiety which is generally considered as a substrate for reduction and not as a catalyst. Herein we demonstrate that *N*‐Me‐2‐aryl‐benzothiazolium salts are able to activate Si−H bonds and are effective as catalysts for dehydrosilylation and hydrosilylation reactions.

## Results and Discussion

**Scheme 4:**
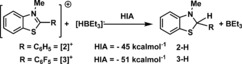
Relative (to BEt_3_) HIA values of **[2]** ^+^ and **[3]** ^+^.

Initially, the hydride ion affinity (HIA)^20^ of the *N*‐methyl‐2‐phenylbenzothiazolium cation (**2** ^+^) relative to BEt_3_ was computationally determined and found to be −45 kcal mol^−1^ (Scheme 4). This is comparable to that calculated previously for B(C_6_F_5_)_3_ (−41, kcal mol^−1^ M06‐2X/6–311G(d,p) with dichloromethane (DCM) solvation (Polarizable Continuum model, PCM). Furthermore, this HIA value is less than that found for **1** ^+^ (−53 kcal mol^−1^)^15^ suggesting **[2]** ^+^ is a more appropriate Lewis acid for use in catalytic imine reductions as its conjugate organic hydride will be more reducing than **1‐H**. The C2‐pentafluorophenyl analogue, **[3]** ^+^_,_ also was calculated and found to have an HIA of −51 kcal mol^−1^ indicating it is less suitable for use in catalytic reductions (as its conjugate hydride, **3‐H**, will be a poorer reductant).

**Figure 1:**
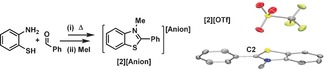
Synthesis and X‐ray structure of **[2][Anion]**. Red=oxygen, yellow=sulfur, grey=carbon, blue=nitrogen, light green=fluorine.

Due to its suitable calculated HIA value *N*‐methyl‐2‐phenyl‐benzothiazolium iodide was synthesized and metathesized with AgOTf, NaBPh_4_ and NaBArCl (BArCl=[B(3,5‐Cl_2_‐C_6_H_3_)_4_]^−^) to afford **[2][Anion]** (Anion=OTf, BPh_4_, BArCl, respectively) in moderate to good yields in each case. Single crystals of the triflate salt were obtained from MeCN/ *ortho*‐dichlorobenzene (*o*‐DCB) which revealed a non‐planar cation with an angle of 47.8° between the plane of the phenyl ring and that of the thiazole ring, with the *N*−Me group preventing a co‐planar arrangement (Figure 1). The closest cation/anion contact involving the electrophilic C2 position is long and involves a triflate oxygen located at 3.324 Å, a distance that is significantly greater than the combined covalent radii of carbon and oxygen.^21^ The low oxophilicity of **[2]** ^+^ indicated by this structure was confirmed by the lack of any evidence for binding of H_2_O to **[2][BArCl]**; furthermore, no O−H heterolytic cleavage was observed on addition of 2,6‐lutidine and H_2_O to a DCM solution of **[2][BArCl]** (in contrast to what is observed with B(C_6_F_5_)_3_ and other Lewis acidic boranes).^22^ The combination of one equivalent of Et_3_PO and three equivalents of **[2][BArCl]** only led to a small downfield shift in the ^31^P{^1^H} resonance (Δ*δ*=4.4 ppm) confirming the weak Lewis acidity of **[2]^+^** towards hard Lewis bases (for comparison B(C_6_F_5_)_3_ gives a Δ*δ* of 33.7 ppm).^23^

**Scheme 5:**
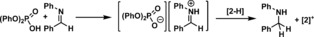
Phosphoric acid initiated *N*‐benzylidine‐aniline reduction with **2‐H**.

Seeking to experimentally assess the relative Lewis acidity of **[2]** ^+^ towards hydride, compound **2‐H** and B(C_6_F_5_)_3_ were combined in DCM. This led to rapid (<5 minutes) hydride transfer and formation of **[2][HB(C_6_F_5_)_3_]**, thus B(C_6_F_5_)_3_ is a stronger Lewis acid towards hydride than **[2]** ^+^. In contrast, combining **[2‐H]** and BPh_3_ led to no hydride transfer, suggesting the Lewis acidity of **[2]** ^+^ towards hydride lies between that of BPh_3_ and B(C_6_F_5_)_3_. Both these boranes can activate H_2_ and Si−H bonds,^1^, 8b, 24 thus analogous bond activations using **[2][Anion]** should be thermodynamically viable. The activation of H−H or Si−H is only the initial step in a putative catalytic cycle, with subsequent hydride transfer to the substrate also required. Recent studies have highlighted the importance of correctly balancing the electrophilicity of the substrate (e.g., an iminium cation) and the reducing power of the organic hydride to achieve successful transfer hydrogenation.^25^ Thus, we investigated the diphenylphosphoric acid initiated reduction of *N*‐benzylidene–aniline, employing **[2‐H]** as the hydride source (Scheme 5). Using equimolar ratios this produced *N*‐benzylaniline in around 50 % conversion, with conversion limited due to the amine product deprotonating the iminium cation (full imine reduction can be achieved by using >2 equivalent of the phosphoric acid). These reactions confirm that **2‐H** is a more accessible source of hydride for reductions than **1‐H**.

### E−H bond activation studies

Compounds **[2][BPh_4_]** and **[2][BArCl]** (5 mol %) both induced H/D scrambling between Et_3_SiD and PhMe_2_SiH, albeit only at raised temperatures (80 °C in MeCN for the former and at 60 °C in DCM for the latter in sealed tubes). In contrast, no scrambling was observed between Ph_3_SiH and Et_3_SiD using **[2][BArCl]** (at 60 °C in DCM for 24 h) presumably due to the greater steric bulk of Ph_3_SiH. This is consistent with the less bulky silane Ph_2_MeSiH, that has a similar kinetic nucleophilicity to Ph_3_SiH (*N* parameter values of 2.72 and 2.65, respectively),^26^ undergoing H/D exchange with Et_3_SiD in the presence of **[2][BArCl]**. Triarylboranes are also competent at activating Si−H bonds,^1^ therefore control reactions were performed to preclude the possibility that a Lewis acidic borane, potentially formed in situ by anion decomposition by trace protic impurities,^27^ is leading to Si−H activation. Notably, utilising the identical batch of NaBPh_4_ used to prepare **[2][BPh_4_]** no H/D scrambling between Et_3_SiD and PhMe_2_SiH was observed under identical conditions (at 5 mol % NaBPh_4_ loading for 20 h at 80 °C in MeCN), thus silane H/D scrambling is being mediated by the carbon Lewis acid **[2]** ^+^. This is further confirmed by **[2][OTf]** also resulting in H/D scrambling between PhMe_2_SiH and Et_3_SiD (at 80 °C in MeCN) albeit more slowly than observed with both borate anions. The requirement for raised temperatures for H/D scrambling with **[2]** ^+^ may in part be attributed to strong anion cation interactions which need to be overcome before silane activation occurs. Indeed the diffusion coefficients (by diffusion ordered spectroscopy (DOSY) experiments) are identical for the cationic and anionic components of **[2][BArCl]** in DCM and in acetone, consistent with the existence of intimate ion pairs in these solvents.^28^

**Scheme 6:**
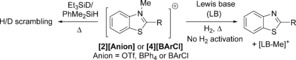
Activation of E−H bonds using benzothiazolium salts. LB for example=P*t*Bu_3_.

FLPs were generated by combining **[2][BArCl]** with equimolar 2,6‐lutidine, 1,8‐bis(dimethylamino)naphthalene and 4‐DMAP. The absence of any observable Lewis adduct between **[2]** ^+^ and 4‐DMAP is notable and in contrast to the reactivity of both **[1]** ^+^ and B(C_6_F_5_)_3_ towards 4‐DMAP,^16^, ^29^ further indicating the low Lewis acidity of **[2]** ^+^ towards hard Lewis bases. A modified benzothiazolium salt containing a *para*‐*t*Bu‐phenyl C2‐substituent, **[4][BArCl]**, was also explored in FLPs with these three amine Lewis bases in an attempt to use the steric bulk provided by a *t*Bu substituent to weaken cation–anion interactions. However, combinations of **[2][BArCl]** or **[4][BArCl]** and these amines resulted in no H_2_ activation (100 °C in *o*‐DCB for 16 h, ca. 4 atm. of H_2_). FLPs containing **[4]** ^+^ and stronger Lewis bases, such as P*t*Bu_3_ and Verkade's base also showed no propensity to activate H_2_ (ca. 4 atm. at 60 °C or 100 °C). In these cases slow demethylation of the benzothiazolium salt proceeded to form, for example, [Me‐P*t*Bu_3_]^+^ (Scheme 6).

### Catalytic studies

Three different benzothiazolium cations containing phenyl (**[2]** ^+^), *p‐t*Bu‐phenyl (**[4]** ^+^) and 1‐naphthyl (**[5]** ^+^) C2‐substituents were evaluated as catalysts in dehydrosilylation and hydrosilylation reactions. Cations **[4]** ^+^ and **[5]** ^+^ were selected to alter steric bulk distal (in the case of **[4]** ^+^) and proximal (**[5]** ^+^) to the C2 electrophilic centre relative to that in **[2]** ^+^. It should be noted that the incorporation of a *para*‐*t*Bu substituent will also affect the electrophilicity at the C2 position, an effect confirmed by HIA calculations which revealed that **[4]** ^+^ is 1 kcal mol^−1^ less Lewis acidic towards hydride relative to **[2]** ^+^. The initial reaction studied was the dehydrosilylation of benzyl alcohol using PhMe_2_SiH.^30^ Although the parent iodide salts (e.g. **[2][I]**) resulted in no dehydrosilylation using the [OTf] and [BPh_4_] salts dehydrosilylation occurred in DCM but was extremely slow (Table 1, entries 1–3). Dehydrosilylation could be accelerated to some extent for the [BPh_4_] salts by using a more polar solvent (e.g., MeCN, Table 1, entries 4, 5). The [BArCl] salts were significantly more active catalysts and thus only salts containing this anion were studied hereon. The three benzothiazolium [BArCl] salts showed minimal differences as catalysts for benzyl alcohol (BnOH) dehydrosilylation (entries 6–9) which proceeded rapidly at 20 °C in DCM in each case.

**Table 1:** Dehydrosilylation of benzyl alcohol with benzothiazolium salts.

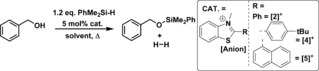					
Entry	Catalyst	Solvent	Time [h]	Temp [°C]	Yield [%]^[a]^
1	**[2][BPh_4_]**	DCM	40	60	8
2	**[5][BPh_4_]**	DCM	40	60	13
3	**[5][OTf]**	DCM	45	60	12
4	**[2][BPh_4_]**	MeCN	24	80	76
5	**[5][BPh_4_]**	MeCN	24	80	72
6	**[2][BArCl]**	DCM	1	20	94
7	**[2][BArCl]**	MTBE	1	20	94
8	**[5][BArCl]**	DCM	1	20	86
9	**[4][BArCl]**	DCM	1	20	84
10	**[4][BArCl]**	MeCN	24	20	60

[a] Conversion by NMR spectroscopy versus mesitylene as an internal standard.

A number of control reactions were performed to ensure that protonolysis of the borate anion is not leading to an active borane catalyst under these conditions. Anion decomposition has been previously reported on heating NaBPh_4_ in wet solvents which led to the formation BPh_3_.^27^, ^31^ Using 5 mol % Na[BPh_4_] (at 80 °C for 24 h in MeCN) or 5 mol % Na[BArCl] (20 h at 20 °C or 5 h at 80 °C in MeCN) no dehydrosilylation of BnOH was observed. In contrast, under identical conditions both **[2][BPh_4_]** and **[4][BArCl]** lead to significant dehydrosilylation (entries 4 and 10). Moreover, BPh_3_ was confirmed to be an active catalyst for the dehydrosilylation of BnOH in MeCN under these conditions (40 % in 20 h at 80 °C), consistent with Okuda's original observation of EtOH dehydrosilylation catalyzed by BPh_3_.8b Thus the complete absence of BnOH dehydrosilylation using Na[B(Aryl)_4_] in MeCN indicates that B(Aryl)_3_ (or any other borane able to initiate dehydrosilylation) is not being formed by anion decomposition. These results combined confirm that under these conditions catalytic BnOH dehydrosilylation is initiated by the benzothiazolium salts and not by Lewis acidic boranes derived from anion decomposition.^32^

**[5][BArCl]** also catalyzes the dehydrosilylation of BnOH in DCM with Ph_3_SiH with 57 % conversion at 60 °C after 24 h. This is slower than with B(C_6_F_5_)_3_ 30 using the same silane/alcohol which proceeds at room temperature. This indicates a significantly greater kinetic barrier using the benzothiazolium salt as catalyst. Phenol also underwent dehydrosilylation catalyzed by **[4][BArCl]** (76 % conversion after 2 h at 20 °C in DCM) precluding an alcohol dehydrogenation/ carbonyl hydrosilylation mechanism. Benzothiazolium salts also dehydrosilylate water to form the respective siloxane, thus alcohol dehydrosilylation proceeds using non‐purified solvents (using excess silane) and also in more environmentally friendly solvents such as methyl *tert*‐butyl ether (MTBE).

Another established B(C_6_F_5_)_3_ catalyzed reaction is the hydrosilylation of carbonyls reported by Piers and co‐workers.^5^ Using the three benzothiazolium[BArCl] salts for carbonyl hydrosilylation led to differences in the rate of benzaldehyde (Table 2 entries 1–3) and acetophenone (Table 2 entries 4–6) hydrosilylation. With both substrates the C2‐(1‐naphthyl) substituted catalyst **[5][BArCl]** results in the slowest rate of hydrosilylation suggesting that either greater steric hindrance around the C2 centre or stronger cation–anion interactions are retarding the rate of hydrosilylation using this catalyst. HIA calculations on **[5]** ^+^ revealed it has an effectively identical Lewis acidity towards hydride as found for **[2]** ^+^ (−45.5 and −45.4 kcal mol^−1^, respectively) precluding the reactivity disparity originating from different degrees of Lewis acidity to hydride (which in turn would lead to differing degrees of silane activation and differing reducing powers of **5‐H** and **2‐H**).

**Table 2:** Hydrosilylation of benzaldehyde and acetophenone.

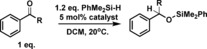				
Entry	Catalyst	R	Time [min]	Yield [%]^[a]^
1	**[2][BArCl]**	H	60	92
2	**[5][BArCl]**	H	60	10
3	**[4][BArCl]**	H	60	90
4	**[2][BArCl]**	Me	15	>99
5	**[5][BArCl]**	Me	15	40
6	**[4][BArCl]**	Me	15	>99

[a] Conversion by NMR spectroscopy versus mesitylene as an internal standard.

Other features of carbonyl hydrosilylation using benzothiazolium salts are comparable to that reported for B(C_6_F_5_)_3_. For example, an equimolar mixture of acetophenone/benzaldehyde/PhMe_2_SiH led to hydrosilylation of the more nucleophilic substrate, benzaldehyde, preferentially in the presence of 5 mol % **[4][BArCl]**. Moreover, attempts to selectively hydrosilylate ethyl benzoate using 1.2 equivalents of PhMe_2_SiH in the presence of **[4][BArCl]** led to mixtures of products consistent with the silyl acetal product undergoing further reduction competitively to ester hydrosilylation, again analogous to that observed using B(C_6_F_5_)_3_.^5^ Finally, a control reaction using 5 mol % NaBArCl in the hydrosilylation of acetophenone resulted in no reaction (after 60 minutes at 20 °C in DCM) indicating that catalytic activity is again due to the benzothiazolium salt.

Benzothiazolium[BArCl] salts were also effective catalysts for the hydrosilylation of imines, albeit at raised temperatures. Whilst all three benzothiazolium catalysts reduced *N*‐benzylidene aniline at 100 °C in *o*‐DCB (Table 3) considerable disparities in catalyst activity were observed in the hydrosilylation of the less electrophilic imine *N*‐benzylidene‐*tert*‐butylamine. Both **[2][BArCl]** and **[5][BArCl]** were inactive for the hydrosilylation of *N*‐benzylidene‐*tert*‐butylamine with PhMe_2_SiH at 60 °C, whilst catalysis did proceed at 100 °C it was extremely slow with both salts. In contrast, **[4][BArCl]** was able to catalyse the hydrosilylation of both of these imines, including *N*‐benzylidene‐*tert*‐butylamine at 60 °C in DCM. The disparities in activity between the benzothiazolium salts are attributed to the lower HIA of **[4]^+^** relative to **[2]** ^+^ and **[5]^+^**. This results in the conjugate hydride of **[4]^+^**, **4‐H**, being a stronger reducing agent thus more effective at reducing the silylated iminium cation derived from *N*‐benzylidene‐*tert*‐butylamine. The effectiveness of benzothiazolium salts in catalytic imine hydrosilylation is therefore dependent on the difference in electrophilicity between the silylated iminium cation and the benzothiazolium cation.

**Table 3:** Imine hydrosilylation using **[*n*][BArCl]** as catalyst.

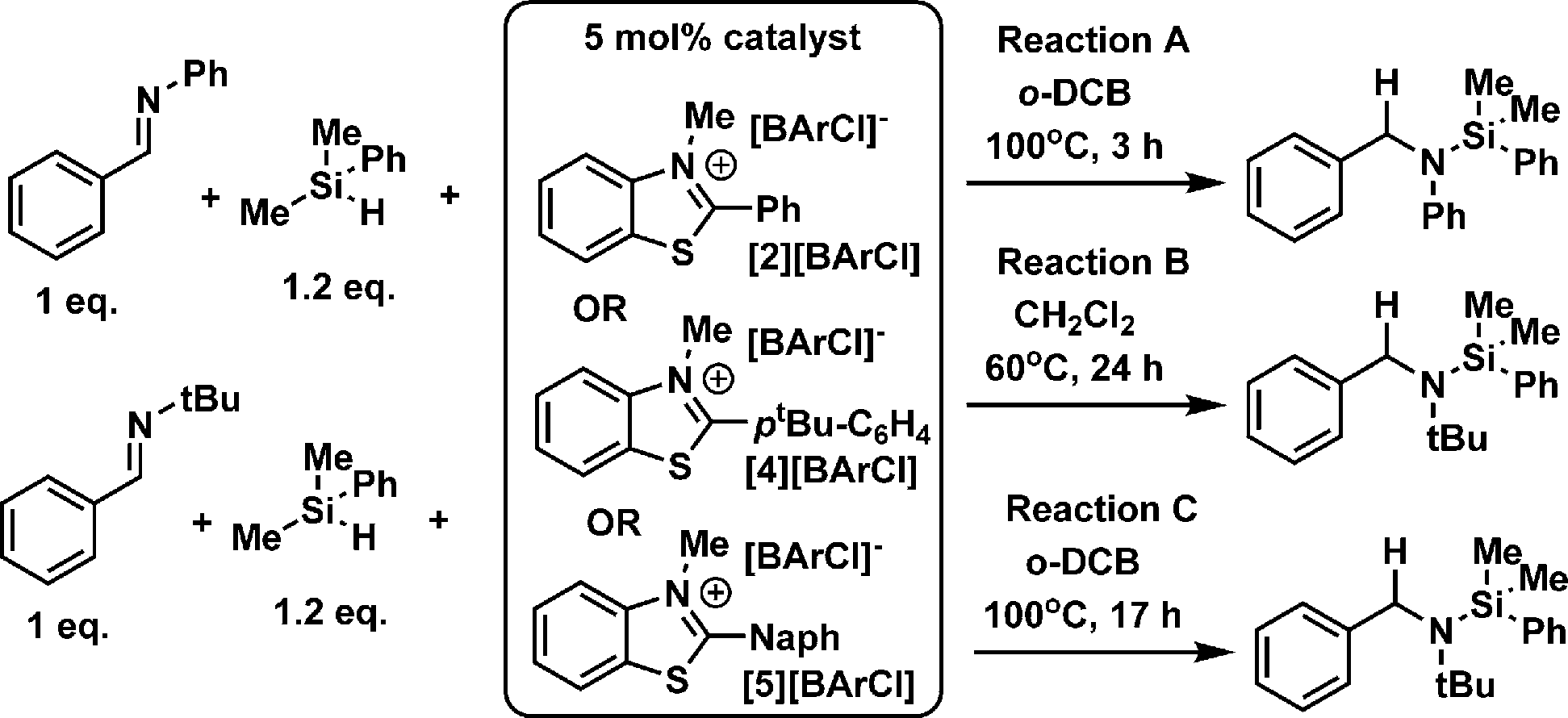				
Catalyst	HIA [kcal mol^−1^]	Yield [%]^[a]^		
		Reaction A	Reaction B	Reaction C
**[2][BArCl]**	−45.4	76	0	24
**[4][BArCl]**	−44.4	60	97	76
**[5][BArCl]**	−45.5	54	0	20

[a] Conversion by NMR spectroscopy versus mesitylene as an internal standard.

**Scheme 7:**
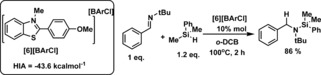
Imine hydrosilylation using **[6][BArCl]**.

A decrease in the HIA of the benzothiazolium cation (relative to **[4]** ^+^) was explored targeting more rapid iminium cation reduction by increasing the reducing power of the conjugate hydride.^26^ Thus **[6][BArCl]** (Scheme 7, left), containing a MeO group in the *para* position of the C2‐phenyl substituent was synthesized using standard procedures. HIA calculations on **[6]** ^+^ confirmed a reduced HIA value, with **[6]** ^+^ being 0.8 kcal mol^−1^ less Lewis acidic towards hydride than **[4]** ^+^, indicating the conjugate hydride **6‐H** should be more reducing than **4‐H**. Utilizing 5 mol % of [**6][BArCl]** the hydrosilylation of *N*‐benzylidene‐*tert*‐butylamine led to more rapid hydrosilylation relative to **[4][BArCl]**, with around 50 % conversion after 3 h at 100 °C. However, heating this reaction for longer did not lead to any further imine hydrosilylation. This is attributed to a catalyst deactivation process as the use of 10 mol % of **[6][BArCl]** led to 86 % hydrosilylation of *N*‐benzylidene‐*tert‐*butylamine in 2 hours (at 100 °C in *o*‐DCB).

With **[4][BArCl]** identified as the more robust catalyst (relative to **[6][BArCl]**) a brief substrate scope exploration was performed. This revealed that using the less bulky silane PhMeSiH_2_ in place of PhMe_2_SiH led to more rapid imine hydrosilylation (Table 4, entries 1 and 2), whilst the bulkier silane Ph_2_MeSiH significantly retarded the rate of hydrosilylation (entries 3, 4). The hydrosilylation of the less hindered imines *N*‐benzylidene‐benzylamine and *N*‐benzylidene‐methylamine were both extremely slow using **[4][BArCl]** (entry 5 and 6). The ^1^H NMR spectra for **[4][BArCl]** revealed no significant changes to the resonances for **[4][BArCl]** before and after addition of *N*‐benzylidene‐methylamine, even with 20 equivalents of *N*‐benzylidene‐methylamine, precluding any appreciable Lewis adduct formation. In contrast, mixtures of B(C_6_F_5_)_3_ and this imine form a strong Lewis adduct.^10^ However, when [**4][BArCl]** was combined with 1 or 5 equivalents of this imine the *N*−Me ^1^H NMR resonance was significantly broadened suggesting a non‐covalent interaction (e.g., H‐bonding or π‐stacking) between the imine and **[4]^+^** which maybe impacting its rate of hydrosilylation. Although **[4][BArCl]** catalyses the hydrosilylation of carbonyls and imines it does not catalyse the hydrosilylation of alkynes (e.g., phenylacetylene or 1‐phenyl‐1‐propyne) with PhMe_2_SiH (even at 100 °C), consistent with the lower Lewis acidity of the **[4]^+^** towards hydride (relative to B(C_6_F_5_)_3_) leading to a lower degree of silane activation and thus a weaker silicon electrophile.8a

**Table 4:** Imine hydrosilylation using **[4][BArCl]** as catalyst.

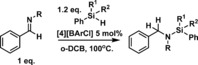					
Entry	R	R^1^	R^2^	Time [h]	Yield [%]^[a]^
1	*t*Bu	Me	Me	17	76
2	*t*Bu	Me	H	17	98
3	Ph	Me	Me	3	60
4	Ph	Ph	Me	44	73
5	CH_2_Ph	Me	Me	72	43^[b]^
6	Me	Me	Me	48	22^[b]^

[a] % Conversion by NMR spectroscopy versus mesitylene as an internal standard. [b] Imine consumption.

**Scheme 8:**
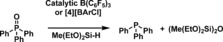
Ph_3_PO reduction using **[4][BArCl]**.

To compare the catalytic activity of **[4][BArCl]** in another reaction involving a nucleophile that forms a Lewis adduct with B(C_6_F_5_)_3_ the reduction of a phosphine oxide was investigated. Oestreich, Stephan and co‐workers have recently utilized B(C_6_F_5_)_3_ to reduce phosphine oxides with silanes.^33^ Replacing B(C_6_F_5_)_3_ with **[4][BArCl]** under identical conditions led to the quantitative reduction of Ph_3_PO to Ph_3_P (Scheme 8), although at a slower rate relative to that catalyzed by B(C_6_F_5_)_3_. This is despite the absence of any observable Lewis adduct on combining equimolar **[4]** ^+^ and Ph_3_PO. A control reaction using 5 mol % NaBArCl resulted in no significant phosphine oxide reduction under identical conditions again indicating catalytic activity is initiated by **[4]^+^**.

### Benzoxazolium and benzimidazolium cations as Lewis acids

**Scheme 9:**
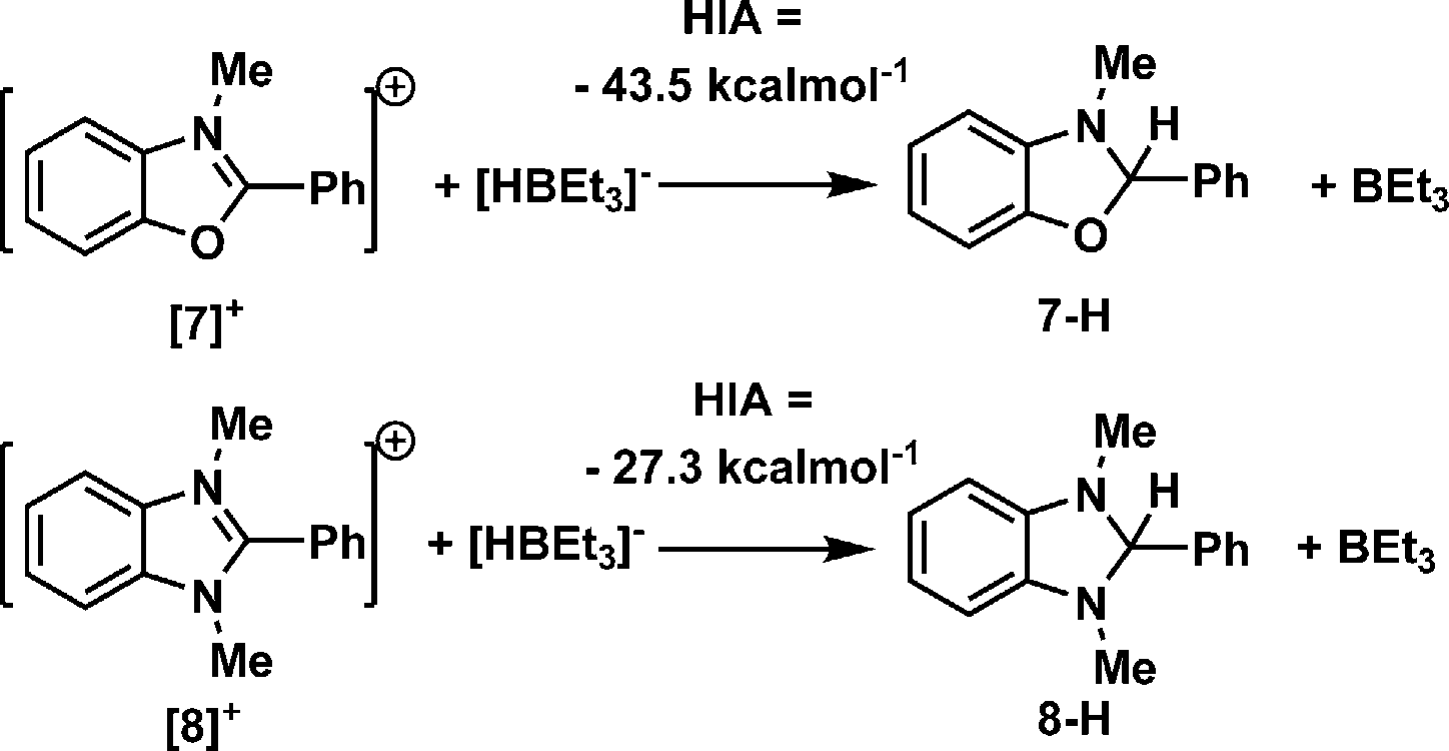
Calculated (relative to BEt_3_) hydride ion affinity values of **[7]^+^**/ **[8]^+^**.

With an understanding of the catalytic ability of *N*‐Me‐benzothiazolium[BArCl] salts in hand the related cations, *N*‐Me‐2‐phenyl‐benzoxazolium, **[7]** ^+^, and *N*,*N*‐Me_2_‐2‐Ph‐benzimidazolium, **[8]** ^+^ were investigated. From previous calorimetry studies *N*‐Me‐2‐Ph‐benzoxazoline, **7‐H**, is reported to have a significantly lower, and *N*,*N*‐Me_2_‐2‐Ph‐benzimidazoline, **8‐H**, a significantly higher hydride donating ability relative to **2‐H**.^34^ The latter was consistent with HIA calculations (Scheme 9), however, **[8]** ^+^ and **[2]** ^+^ were calculated to have HIA values within 2 kcal mol^−1^ of each other. In fact, the only major significant calculated difference between **[2]** ^+^ and **[7]** ^+^ is related to the charge distribution, with a more polarized σ‐bonding framework in **[7]** ^+^ leading to a greater positive NBO charge localized at C2 in **[7]** ^+^ relative to **[2]** ^+^ (+0.56 and +0.20, respectively).

Salts **[7][I]** and **[8][I]** were obtained by methylating the neutral precursors with MeI. Subsequent metathesis with NaBArCl provided **[7][BArCl]** and **[8][BArCl]**. Confirmation of the greater hydride donating ability of **8‐H** relative to **2‐H** and **7‐H** was confirmed by the combination of **8‐H** with **[2][BArCl]** (or **[7][BArCl]**). This led to complete consumption of **8‐H** and the formation of **[8][BArCl]** and **2‐H** (or **7‐H**), after heating to 60 °C for 2 h in DCM (Scheme 9). Despite the increased magnitude of positive charge at C2 in **[7][BArCl]** there is no evidence for binding of H_2_O or one equivalent of Et_3_PO (by NMR spectroscopy) indicating **[7]** ^+^ is a weak Lewis acid toward hard Lewis bases. Consistent with this the combination of **[7][BArCl]** with 2,6‐lutidine, 4‐DMAP and P*t*Bu_3_ resulted in FLP formation with no evidence for any coordination to **[7]^+^**. However, no H_2_ activation was observed for any of these FLP combinations. In contrast, both **[7][BArCl]** and **[8][BArCl]** were effective for the activation of silanes, with H/D exchange observed between Et_3_SiD and PhMe_2_SiH. To assess the catalytic activity of **[7][BArCl]** and **[8][BArCl]** relative to **[2][BArCl]** (the latter selected as it has an identical C2‐substitutent) the hydrosilylation of *N*‐benzylidene‐*tert*‐butylamine was explored (Table 5). This was selected as it is a reaction that was found to be sensitive to variation in the C2‐substituents of the benzothiazolium salts. Both **[7][BArCl]** and **[8][BArCl]** were active for the hydrosilylation of imines confirming that they are also effective Lewis acid catalysts, with conversions similar to that observed for **[2][BArCl]**. The small differences in relative conversions are attributed to different degrees of silane activation (lower with the less Lewis acidic **[8]^+^**) and reducing powers of the conjugate hydride (higher in **[8‐H]**).

**Table 5:** Imine hydrosilylation using **[*n*][BArCl]** as catalyst.

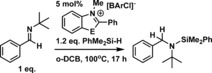		
Entry	E (Catalyst)	Conversion [%]^[a]^
1	S (**[2][BArCl]**)	24
2	O (**[7][BArCl]**)	25
3	NMe (**[8][BArCl]**)	38

[a] conversion by NMR spectroscopy versus mesitylene as an internal standard.

## Conclusion

*N*‐Me‐C2‐Aryl‐benzothiazolium cations represent a new family of readily tuned Lewis acids that show activity in frustrated Lewis pair (FLP) chemistry. They are based on cationic iminium moieties containing an electrophilic carbon centre that has a Lewis acidity towards hydride comparable to the triarylboranes widely used in FLP reactivity. However, in contrast to the triarylboranes these cations show little propensity to bind hard Lewis bases such as, H_2_O, Et_3_PO and 4‐DMAP. A range of benzothiazolium salts “partially” activate the Si−H bond of silanes, as indicated by H/D scrambling. Furthermore, they are effective catalysts^35^ in a range of established FLP‐type (de)hydrosilylation reactions with rational tuning of the C2‐aryl substituent enhancing catalytic activity. The ability of a cationic iminium moiety to initiate catalytic (de)hydrosilylation reactions by activation of silane σ‐bonds is notable as these moieties are viewed generally as substrates for reduction in FLP chemistry and not as catalysts themselves.

## Experimental Section

Supporting Information for this article includes experimental details, spectra, computational and crystallographic data. CCDC 1501133 contains the supplementary crystallographic data for this paper. These data are provided free of charge by The Cambridge Crystallographic Data Centre.

## Supporting information

As a service to our authors and readers, this journal provides supporting information supplied by the authors. Such materials are peer reviewed and may be re‐organized for online delivery, but are not copy‐edited or typeset. Technical support issues arising from supporting information (other than missing files) should be addressed to the authors.

Supplementary
