# Supplementary material for: N‐Methyl‐Benzothiazolium Salts as Carbon Lewis Acids for Si−H σ‐Bond Activation and Catalytic (De)hydrosilylation
Source: Chemistry. 2016 Nov 22;23(1):187–93. doi: 10.1002/chem.201604613 (PMC5396135; doi:10.1002/chem.201604613)
Supplement: Supplementary file 1 — Supplementary [file CHEM-23-187-s001.pdf]

CHEMISTRY
A European Journal

Supporting Information

N-Methyl-Benzothiazolium Salts as Carbon Lewis Acids for Si–H σ-Bond Activation and Catalytic (De)hydrosilylation

Valerio Fasano, James E. Radcliffe, Liam D. Curless, and Michael J. Ingleson*[a]

chem_201604613_sm_miscellaneous_information.pdf

Table of Contents

1. General Remarks 2
2. Synthesis of benzothiazolium salts 2
2.1 General procedure for the synthesis of benzothiazole 2
2.2 General procedure for the synthesis of benzothiazolium iodide salts 2
2.3 General procedure for the anion metathesis 3
2.4 Benzothiazolium salts data 4
3. Hydride abstraction from *N*-methyl-2-phenyl-benzothiazoline by $B(C_6F_5)_3$ 7
4. Binding interaction study between [2][BArCl] and 2,6-lutidine/water 8
5. Gutmann-Beckett method measurements 9
6. Attempted H_2 activation experiments 10
6.1 General procedure for H_2 activation experiments 10
6.2 [2][BArCl] (or [4][BArCl]) with nitrogen based Lewis bases 10
6.3 [4][BArCl] with phosphorus based Lewis base 10
7. Acid initiated stepwise reduction of *N*-benzylidene-aniline with *N*-methyl-2-phenyl-benzothiazoline 12
8. Silane activation: H/D scrambling 13
8.1 Control experiment using $NaBPh_4$ 16
9. Dehydrosilylation of benzyl alcohol 17
9.1 General procedure for the dehydrosilylation of benzyl alcohol 17
9.2 Benzyloxy-dimethylphenylsilanes data 17
9.3 Control experiment using NaBArCl, $NaBPh_4$ or BPh_3 22
9.4 Dehydrosilylation of phenol 25
10. Hydrosilylation of aldehydes and ketones 26
10.1 General procedure for hydrosilylation of benzaldehyde/acetophenone 26
10.2 Alkoxysilanes data 26
10.3 Control experiment using NaBArCl 28
11. Hydrosilylation of imines 28
11.1 General procedure for hydrosilylation of imines 28
11.2 *N*-silyl-amines data 29
11.3 Interaction study between $[4]^+$ and *N*-benzylidene-methylamine 35
12. Attempted hydrosilylation of alkynes 36
13. Deoxygenation of phosphine oxide 38
13.1 Control experiment using NaBArCl 39
14. Benzoxazolium and benzimidazolium catalysts 40
14.1 Synthesis of benzoxazolium and benzimidazolium salts 40
14.2 Hydride transfer reactions 41
14.3 Hydrosilylation of *N*-benzylidene-t-butylamine with [7][BArCl] and [8][BArCl] 44
15. X-Ray Crystallography 45
16. Carbon Lewis acid HIA calculation coordinates 46
17. References 58
18. NMR Spectra of all benzothiazolium salts 59

1. General Remarks

Unless otherwise indicated, all manipulations were conducted using standard Schlenk techniques or in an argon-filled MBraun glovebox (O_2 levels below 0.5 ppm). Solvents were purified by Innovative Technology PS-MD-5 solvent purification system or distilled from appropriate drying agents, degassed and stored over molecular sieves. All compounds were purchased from commercial sources and used as received. Solvents for column chromatography were of technical grade and used without further purification. Column chromatography was performed on silica gel (230-400 mesh). NMR spectra were recorded with a Bruker AV-400 spectrometer (400 MHz ^{1}H; 100 MHz ^{13}C; 128 MHz ^{11}B; 376 MHz ^{19}F; 79 MHz ^{29}Si, 81 MHz ^{31}P). ^{1}H-NMR chemical shifts are reported in ppm relative to *protio* impurities in the deuterated solvents and ^{13}C-NMR using the solvent resonances unless otherwise stated. ^{11}B-NMR spectra were referenced to external BF_3:Et_2O, ^{19}F to Cl_3CF, ^{29}Si to $Si(CH_3)_4$ and ^{31}P to 85% H_3PO_4. Coupling constants *J* are given in Hertz (Hz), while the multiplicity of the signals are indicated as "s", "d", "t" "pent", "sept" or "m" for singlet, doublet, triplet, pentet, septet or multiplet, respectively. Mesitylene (distilled from K) was used as an internal standard to determine the NMR yields. GC-MS analysis was performed on an Agilent Technologies 7890A GC system equipped with an Agilent Technologies 5975C inert XL EI/CI MSD with triple axis detector. The column employed was an Agilent J&W HP-5ms ((5%-Phenyl)-methylpolysiloxane) of dimensions: length, 30 m; internal diameter, 0.250 mm; film, 0.25 μm. Mass spectra were recorded on a Waters QTOF mass spectrometer.

2. Synthesis of benzothiazolium salts

2.1 General procedure for the synthesis of benzothiazole (A)

To a solution of 2-aminothiophenol (1.00 eq) in EtOH, the appropriate aldehyde ArCHO (1.00 eq.) was added. The solution was stirred for 2 days in an aerobic atmosphere with air bubbled through the solution, producing a white precipitate which was separated from the solution by filtration and dried under vacuum (2-Ar-benzothiazole). The spectroscopic data of the 2-Ar-benzothiazoles obtained were in accordance with those reported in the literature.[1]

2.2 General procedure for the synthesis of benzothiazolium iodide salts (B)

2-Ar-benzothiazole was dissolved in an excess of iodomethane (10.0 eq.), within a sealed ampule fitted with a J. Young's stopcock. The reaction vessel was heated at 60°C for 4 days and then iodomethane was removed under vacuum, leaving a dark yellow solid. The latter was washed with hexane affording *N*-methyl-2-Ar-benzothiazolium iodide as a yellow solid. The unreacted 2-Ar-benzothiazole could be recovered from the hexane phase.

Note: MeI is strong methylating agent (treat it in the fumehood at all times) and it is quenched using a NaOH solution.

2.3 General procedure for the anion metathesis (C)

To a solution of *N*-methyl-2-Ar-benzothiazolium iodide (1.00 eq) in DCM, the metathesis reagent* (1.30 eq.) was added (in case of AgOTf the reaction was carried out in the dark). The solution was stirred overnight at room temperature, producing a precipitate. After filtration, the solution was dried under vacuum, obtaining [*N*-methyl-2-Ar-benzothiazolium][counterion] as a solid.

$*AgOTf, NaBPh_4$ or NaBArCl (the latter was prepared according to the literature)[2]

2.4 Benzothiazolium salts data

2-phenyl-benzothiazole: Prepared according to general procedure A**.** 2-aminothiophenol (1.62 mL, 15.00 mmol, 1.00 eq.) and PhCHO (1.56 mL, 15.00 mmol, 1.00 eq.) in EtOH (30 mL) gave 2-phenyl-benzothiazole as a white solid (1.77 g, 8.40 mmol, 56%). The spectroscopic data of the 2-Ph-benzothiazole was in accordance with those reported in the literature.[1]

***N*-methyl-2-phenyl-benzothiazolium iodide ([2][I]):** Prepared according to general procedure B**.** 2-phenyl-benzothiazole (1.16 g, 5.50 mmol, 1.00 eq.) in excess of MeI (10.00 eq.) gave *N*-methyl-2-phenyl-benzothiazolium iodide as a yellow solid (0.72 g, 2.00 mmol, 37%). ^{1}H-NMR (400 MHz, $CDCl_3$) δ 8.40 (d, *J* = 8.2 Hz, 1H), 8.19 (d, *J* = 8.6 Hz, 1H), 8.05 (d, *J* = 7.3 Hz, 2H), 7.89 (t, *J* = 8.0 Hz, 1H), 7.83-7.68 (m, 4H) 4.53 (s, 3H) ppm. $^{13}C\{^1H\}$ NMR (100 MHz, $CDCl_3$) δ 173.8, 142.5, 134.2, 131.0, 130.5, 130.0, 129.6, 129.1, 124.8, 124.4, 117.9, 39.6 ppm. MS: m/z calcd for $C_{14}H_{12}NS^+$ (**[2]**$^+$) 226.1 Found ES$^+$ 226.1, m/z calcd for I^- (**[I]**$^-$) 126.9 Found ES$^-$ 126.9; Accurate mass for $C_{14}H_{12}NS^+$ (**[2]**$^+$) 226.0685 Found 226.0690.

***N*-methyl-2-phenyl-benzothiazolium triflate ([2][OTf]):** Prepared according to general procedure C**.** *N*-methyl-2-phenyl-benzothiazolium iodide (0.18 g, 0.50 mmol, 1.00 eq.) and AgOTf (0.17 g, 0.65 mmol, 1.30 eq.) in DCM (2 mL) gave *N*-methyl-2-phenyl-benzothiazolium triflate as a grey solid (0.18 g, 0.47 mmol, 94%). ^{1}H-NMR (400 MHz, $CDCl_3$): δ 8.18 (dd, *J* = 8.2 Hz, *J* = 3.7 Hz, 2H), 7.92-7.89 (m, 3H), 7.81-7.77 (m, 2H), 7.74-7.71 (m, 2H), 4.41 (s, 3H) ppm. $^{13}C\{^1H\}$ NMR (100 MHz, $CDCl_3$) δ 174.2, 142.4, 134.3, 130.5, 130.4, 130.1, 129.5, 129.3, 124.8, 123.7 (J_{CF} = 320.0 Hz), 123.4, 117.8, 38.3 ppm. ^{19}F-NMR (400 MHz, $CDCl_3$) δ – 78.30 ppm. MS: m/z calcd for $C_{14}H_{12}NS^+$ (**[2]**$^+$) 226.1 Found ES$^+$ 226.1, m/z calcd for $CF_3SO_3^-$ (**[OTf]**$^-$) 149.0 Found ES$^-$ 149.0.

***N*-methyl-2-phenyl-benzothiazolium tetraphenylborate ([2][BPh$_4$]):** Prepared according to general procedure C**.** *N*-methyl-2-phenyl-benzothiazolium iodide (0.05 g, 0.15 mmol, 1.00 eq.) and $NaBPh_4$ (0.07 g, 0.19 mmol, 1.30 eq.) in DCM (2 mL) gave *N*-methyl-2-phenyl-benzothiazolium tetraphenylborate as a white solid (0.04 g, 0.08 mmol, 50%). ^{1}H-NMR (400 MHz, $(CD_3)_2CO$): δ 8.54 (d, *J* = 8.2 Hz, 1H), 8.44 (d, *J* = 8.4 Hz, 1H), 8.04-8.07 (m, 3H), 7.91-7.99 (m, 2H), 7.85 (d, *J* = 7.8 Hz, 2H), 7.31-7.36 (m, 8H), 6.91 (t, *J* = 7.4 Hz, 8H), 6.77 (t, *J* = 7.2 Hz, 4H), 4.49 (s, 3H) ppm. $^{13}C\{^1H\}$ NMR (100 MHz, $(CD_3)_2CO$): δ 176.2, 165.0 (q, J_{B-C} = 48.9 Hz, BPh_4), 142.7, 137.1 (BPh_4, ArH), 135.1, 131.4, 131.3, 131.1, 131.0, 130.1,

126.5, 126.1 (q, $J_{B\text{-}C}$ = 2.1 Hz, BPh_4, ArH), 125.3, 122.3 (BPh_4, ArH), 118.5, 38.9 ppm. ^{11}B-NMR (128 MHz, $(CD_3)_2CO$): δ -6.55 ppm. MS: m/z calcd for $C_{14}H_{12}NS^+$ (**[2]**$^+$) 226.1 Found ES$^+$ 226.1, m/z calcd for $BC_{24}H_{20}$ (**[BPh4]**$^-$) 319.2 Found ES$^-$ 319.3.

***N*-methyl-2-phenyl-benzothiazolium tetra(3,5-dichlorophenyl)borate ([2][BArCl]):** Prepared according to general procedure C**.** *N*-methyl-2-phenyl-benzothiazolium iodide (0.18 g, 0.50 mmol, 1.00 eq.) and NaBArCl (0.40 g, 0.65 mmol, 1.30 eq.) in DCM (2 mL) gave *N*-methyl-2-phenyl-benzothiazolium tetra(3,5-dichlorophenyl)borate as a white solid after recrystallization from hot DCM (0.16 g, 0.19 mmol, 38%). ^{1}H-NMR (400 MHz, $CDCl_3$) δ 8.02-7.97 (m, 1H), 7.90-7.82 (m, 3H), 7.73 (t, *J* = 7.8 Hz, 2H), 7.55-7.51 (m, 2H), 7.51-7.47 (m, 1H), 6.99-7.04 (m, 8H), 6.83 (t, *J* = 2.0 Hz, 4H) 3.92 (s, 3H) ppm. ^{13}C{^{1}H} NMR (100 MHz, $CDCl_3$) δ 174.1, 164.8 (q, $J_{B\text{-}C}$ = 49.9 Hz, BArCl), 141.7, 135.1, 133.1 (BArCl, ArCl), 133.0 (q, $J_{B\text{-}C}$ = 3.9 Hz, BArCl, ArH), 131.2, 130.6, 130.0, 129.9, 129.1, 123.9, 123.7, 123.1 (BArCl, ArH), 116.8, 37.9 ppm. ^{11}B-NMR (128 MHz, $CDCl_3$): δ -6.95 ppm. MS: m/z calcd for $C_{14}H_{12}NS^+$ (**[2]**$^+$) 226.1 Found ES$^+$ 226.1, m/z calcd for $BC_{24}H_{12}Cl_8$ (**[BArCl]**$^-$) 594.8 Found ES$^-$ 595.0.

2-(1-naphthyl)-benzothiazole: Prepared according to general procedure A**.** 2-aminothiophenol (1.62 mL, 15.00 mmol, 1.00 eq.) and 1-naphthylaldehyde (2.14 mL, 15.00 mmol, 1.00 eq.) in EtOH (30 mL) gave 2-(1-naphthyl)-benzothiazole as a white solid (2.43 g, 9.30 mmol, 62%). The spectroscopic data of 2-(1-naphthyl)-benzothiazole was in accordance with those reported in the literature.[1]

***N*-methyl-2-(1-naphthyl)-benzothiazolium iodide ([5][I]):** Prepared according to general procedure B**.** 2-(1-naphthyl)-benzothiazole (2.00 g, 7.60 mmol, 1.00 eq.) in excess of MeI (10.00 eq.) gave *N*-methyl-2-(1-naphthyl)-benzothiazolium iodide as a yellow solid (1.30 g, 3.20 mmol, 42%). ^{1}H-NMR (400 MHz, $CDCl_3$): δ 8.47 (d, *J* = 8.2 Hz, 1H), 8.42 (d, *J* = 8.6 Hz, 1H), 8.32 (d, *J* = 7.3 Hz, 1H), 8.25 (d, *J* = 8.3 Hz, 1H), 8.05-8.10 (m, 1H), 7.98 (t, *J* = 7.5 Hz, 1H), 7.87 (t, *J* = 7.5 Hz, 1H), 7.77 (t, *J* = 7.3 Hz, 1H), 7.66-7.72 (m, 2H), 7.50-7.55 (m, 1H), 4.29 (s, 3H) ppm. ^{13}C{^{1}H} NMR (100 MHz, $CDCl_3$): δ 172.8, 142.2, 134.3, 133.3, 132.4, 130.7, 130.5, 130.0, 129.5 (3C), 127.8, 125.6, 124.5, 123.3, 121.1, 118.1, 39.6 ppm. MS: m/z calcd for $C_{18}H_{14}NS^+$ (**[5]**$^+$) 276.4 Found ES$^+$ 276.5, m/z calcd for I^- (**[I]**$^-$) 126.9 Found ES$^-$ 126.9; Accurate mass for $C_{18}H_{14}NS^+$ (**[5]**$^+$) 276.0841 Found 276.0833.

***N*-methyl-2-(1-naphthyl)-benzothiazolium tetraphenylborate ([5][BPh4]):** Prepared according to general procedure C**.** *N*-methyl-2-(1-naphthyl)-benzothiazolium iodide (0.10 g, 0.25 mmol, 1.00 eq.) and $NaBPh_4$ (0.11 g, 0.32 mmol, 1.30 eq.) in DCM (2 mL) gave *N*-methyl-2-(1-naphthyl)-benzothiazolium tetraphenylborate as a white solid (0.13 g, 0.21 mmol, 86%). ^{1}H-NMR (400 MHz, $(CD_3)_2CO$): δ 8.58 (d, *J* = 7.6 Hz, 1H), 8.45 (d, *J* = 8.3 Hz, 2H), 8.24 (d, *J* = 7.3 Hz, 1H), 8.10 (t, *J* = 8.6 Hz, 2H), 8.01 (t, *J* = 8.3 Hz, 1H), 7.84-7.73 (m, 2H), 7.69-7.81 (m, 2H), 7.29-7.37 (m, 8H), 6.90 (*t*, *J* = 7.6 Hz, 8H), 6.76 (t, *J* = 7.1 Hz, 4H), 4.25 (s, 3H) ppm. ^{13}C{^{1}H} NMR (100 MHz, $(CD_3)_2CO$): δ 175.0, 165.0 (q, $J_{B\text{-}C}$ = 48.9 Hz, BPh_4), 143.7, 137.1 (BPh_4, ArH), 135.3, 134.6, 132.2, 132.0, 131.4, 131.3, 130.3, 130.2, 130.1, 128.8, 126.4, 126.1 (q, $J_{B\text{-}C}$ = 2.1 Hz, BPh_4, ArH), 125.3, 125.1, 122.9, 122.3 (BPh_4, ArH), 118.7, 38.8 ppm. ^{11}B-NMR (128 MHz, $(CD_3)_2CO$): δ -6.51 ppm. MS: m/z calcd for

$C_{18}H_{14}NS^+$ (**[5]**$^+$) 276.4 Found ES$^+$ 276.5, m/z calcd for $BC_{24}H_{20}$ (**[BPh$_4$]**$^-$) 319.2 Found ES$^-$ 319.3.

***N*-methyl-2-(1-naphthyl)-benzothiazolium tetra(3,5-dichlorophenyl)borate ([5][BArCl]):** Prepared according to general procedure C. *N*-methyl-2-(1-naphthyl)-benzothiazolium iodide (0.10 g, 0.25 mmol, 1.00 eq.) and NaBArCl (0.20 g, 0.32 mmol, 1.30 eq.) in DCM (2 mL) gave *N*-methyl-2-(1-naphthyl)-benzothiazolium tetra(3,5-dichlorophenyl)borate as a white solid after recrystallization from hot DCM (0.19 g, 0.22 mmol, 87%). ^{1}H-NMR (400 MHz, ($CDCl_3$): δ 8.30 (d, *J* = 7.6 Hz, 1H), 8.11 (d, *J* = 7.6 Hz, 1H), 7.94-8.00 (m, 1H), 7.80-7.89 (m, 2H), 7.51-7.75 (m, 6H), 6.99-7.04 (m, 8H), 6.80 (t, *J* = 2.0 Hz, 4H), 3.72 (s, 3H) ppm. ^{13}C{^{1}H} NMR (100 MHz, $CDCl_3$): δ 173.7, 164.5 (q, $J_{B\text{-}C}$ = 48.9 Hz, BArCl), 141.1, 135.4, 133.5, 133.1 (BArCl, ArCl), 132.9 (q, $J_{B\text{-}C}$ = 3.9 Hz, BArCl, ArH), 131.4, 130.9, 130.4, 130.0, 129.9, 129.7, 129.6, 128.4, 125.4, 123.7, 123.0 (BArCl, ArH), 122.5, 120.2, 116.5, 37.7 ppm. ^{11}B-NMR (128 MHz, $CDCl_3$): δ -6.95 ppm. MS: m/z calcd for $C_{18}H_{14}NS^+$ (**[5]**$^+$) 276.4 Found ES$^+$ 276.5, m/z calcd for $BC_{24}H_{12}Cl_8$ (**[BArCl]**$^-$) 594.8 Found ES$^-$ 595.0.

2-(4-*t*-butylphenyl)-benzothiazole: Prepared according to general procedure A. 2-aminothiophenol (1.62 mL, 15.00 mmol, 1.00 eq.) and 4-*t*-butylbenzaldehyde (2.59 mL, 15.00 mmol, 1.00 eq.) in EtOH (30 mL) gave 2-(4-*t*-butylphenyl)-benzothiazole as a white solid (2.20 g, 8.25 mmol, 55%). The spectroscopic data of 2-(4-t-butylphenyl)-benzothiazole was in accordance with those reported in the literature.[1]

***N*-methyl-2-(4-*t*-butylphenyl)benzothiazolium iodide ([4][I]):** Prepared according to general procedure B. 2-(4-*t*-butylphenyl)-benzothiazole (2.00 g, 7.50 mmol, 1.00 eq.) in excess of MeI (10.00 eq.) gave *N*-methyl-2-(4-*t*-butylphenyl)-benzothiazolium iodide as a yellow solid (1.16 g, 2.80 mmol, 38%). ^{1}H-NMR (400 MHz, $CDCl_3$): 8.42 (d, *J* = 8.2 Hz, 1H), 8.16 (d, *J* = 8.6 Hz, 1H), 7.94 (d, *J* = 8.3 Hz, 2H), 7.81 (t, *J* = 8.2 Hz, 1H), 7.63-7.73 (m, 3H), 4.49 (s, 3H), 1.38 (s, 9H) ppm. ^{13}C{^{1}H} NMR (100 MHz, $CDCl_3$): δ 173.7, 158.4, 142.4, 130.8, 130.3, 129.4, 128.8, 127.1, 124.6, 121.9, 117.7, 39.7, 35.4, 30.9 ppm. MS: m/z calcd for $C_{18}H_{20}NS^+$ (**[4]**$^+$) 282.4 Found ES$^+$ 282.1, m/z calcd for I^- (**[I]**$^-$) 126.9 Found ES$^-$ 126.8; Accurate mass for $C_{18}H_{20}NS^+$ (**[4]**$^+$) 282.1311 Found 282.1304.

***N*-methyl-2-(4-*t*-butylphenyl)benzothiazolium tetra(3,5-dichlorophenyl)borate ([4][BArCl]):** Prepared according to general procedure C. *N*-methyl-2-(4-*t*-butylphenyl)-benzothiazolium iodide (0.10 g, 0.25 mmol, 1.00 eq.) and NaBArCl (0.20 g, 0.32 mmol, 1.30 eq.) in DCM (2 mL) gave *N*-methyl-2-(4-*t*-butylphenyl)-benzothiazolium tetra(3,5-dichlorophenyl)borate as a white solid after recrystallization from hot DCM (0.09 g, 0.10 mmol, 42%). ^{1}H-NMR (400 MHz, ($CDCl_3$): δ 7.84-7.79 (m, 1H), 7.71-7.75 (m, 2H), 7.65 (d, *J* = 8.6 Hz, 2H), 7.33-7.40 (m, 3H), 7.06-7.08 (m, 8H), 6.83 (t, *J* = 2.0 Hz, 4H), 3.84 (s, 3H), 1.40 (s, 9H) ppm. ^{13}C{^{1}H} NMR (100 MHz, $CDCl_3$): δ 174.4, 164.4 (q, $J_{B\text{-}C}$ = 48.9 Hz, BArCl), 159.9, 141.6, 132.9-133.1 (BArCl, ArCl+ArH), 131.0, 129.8, 129.7, 128.7, 127.8, 123.4, 123.1 (BArCl, ArH), 120.9, 116.2, 37.4, 35.6, 30.8 ppm. ^{11}B-NMR (128 MHz, $CDCl_3$): δ -6.90 ppm. MS: m/z calcd for $C_{18}H_{20}NS^+$ (**[4]**$^+$) 282.4 Found ES$^+$ 282.1, m/z calcd for $BC_{24}H_{12}Cl_8$ (**[BArCl]**$^-$) 594.8 Found ES$^-$ 595.0.

2-(4-methoxyphenyl)-benzothiazole: Prepared according to general procedure A**.** 2-aminothiophenol (1.62 mL, 15.00 mmol, 1.00 eq.) and 4-methoxybenzaldehyde (1.86 mL, 15.00 mmol, 1.00 eq.) in EtOH (30 mL) gave 2-(4-methoxyphenyl)-benzothiazole as a white solid (1.90 g, 7.80 mmol, 52%). The spectroscopic data of 2-(4-methoxyphenyl)-benzothiazole was in accordance with those reported in the literature.[1]

***N*-methyl-2-(4-methoxyphenyl)-benzothiazolium iodide ([6][I]):** Prepared according to general procedure B**.** 2-(4-methoxyphenyl)-benzothiazole (0.49 g, 2.00 mmol, 1.00 eq.) in excess of MeI (10.00 eq.) gave *N*-methyl-2-(4-methoxyphenyl)-benzothiazolium iodide as a yellow solid (0.12 g, 0.30 mmol, 17%).^{1}H-NMR (400 MHz, $CDCl_3$): δ 8.29 (d, *J* = 8.2 Hz, 1H), 8.16 (d, *J* = 8.5 Hz, 1H), 8.06 (d, *J* = 8.5 Hz, 2H), 7.87 (t, *J* = 7.6 Hz, 1H), 7.75 (t, *J* = 7.9 Hz, 1H), 7.21 (d, *J* = 8.5 Hz, 2H), 4.56 (s, 3H), 3.96 (s, 3H) ppm. $^{13}C\{^1H\}$ NMR (100 MHz, $CDCl_3$): δ 173.8, 164.7, 142.6, 133.2, 130.4, 129.0, 128.9, 123.9, 117.7, 116.6, 115.8, 56.0, 39.8 ppm. MS: m/z calcd for $C_{15}H_{14}NOS^+$ (**[6]**$^+$) 256.1 Found ES^+ 256.0, m/z calcd for I^- (**[I]**$^-$) 126.9 Found ES^- 126.9; Accurate mass for $C_{15}H_{14}NOS^+$ (**[6]**$^+$) 256.0791 Found 256.0778.

***N*-methyl-2-(4-methoxyphenyl)-benzothiazolium tetra(3,5-dichlorophenyl)borate ([6][BArCl]):** Prepared according to general procedure C**.** *N*-methyl-2-(4-methoxyphenyl)-benzothiazolium iodide (0.12 g, 0.31 mmol, 1.00 eq.) and NaBArCl (0.25 g, 0.40 mmol, 1.30 eq.) in DCM (2 mL) gave *N*-methyl-2-(4-methoxyphenyl)-benzothiazolium tetra(3,5-dichlorophenyl)borate as a white solid after recrystallization from hot DCM (0.05 g, 0.14 mmol, 45%). ^{1}H-NMR (400 MHz, $CDCl_3$) δ 7.95 (d, *J* = 7.8 Hz, 1H), 7.77-7.84 (m, 2H), 7.46-7.51 (m, 1H), 7.42 (d, *J* = 8.5 Hz, 2H), 7.10 (d, *J* = 8.5 Hz, 2H), 7.04-7.09 (m ,8H), 6.86 (t, *J* = 1.4 Hz, 4H), 3.96 (s, 3H), 3.89 (s, 3H) ppm. $^{13}C\{^1H\}$ NMR (100 MHz, $CDCl_3$) δ 174.0, 165.2, 164.3 (q, $J_{B\text{-}C}$ = 49.9 Hz, BArCl), 141.7, 133.0 (BArCl, ArCl), 132.9 (q, $J_{B\text{-}C}$ = 3.9 Hz, BArCl, ArH), 132.0, 131.7, 130.8, 129.6, 128.3, 123.4, 123.1 (BArCl, ArH), 116.2, 115.6, 56.1, 37.5 ppm. ^{11}B-NMR (128 MHz, $CDCl_3$): δ -6.91 ppm. MS: m/z calcd for $C_{15}H_{14}NOS^+$ (**[6]**$^+$) 256.0 Found ES^+ 256.4, m/z calcd for $BC_{24}H_{12}Cl_8$ (**[BArCl]**$^-$) 594.8 Found ES^- 594.9.

3. Hydride abstraction from *N*-methyl-2-phenyl-benzothiazoline by $B(C_6F_5)_3$

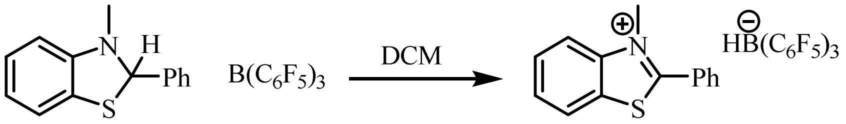

A J. Youngs NMR tube equipped with a DMSO-d_6 capillary was loaded with *N*-methyl-2-phenyl-benzothiazoline (23.0 mg, 0.100 mmol, 1.00 eq.) and dichloromethane (0.5 mL). Then, $B(C_6F_5)_3$ (51 mg, 0.100 mmol, 1.00 eq.) was added to the solution. After stirring at room temperature for 5 min, quantitative formation of **[2][HB(C₆F₅)₃]** was observed by multinuclear NMR spectroscopy. Diagnostic peaks: ^{1}H-NMR (400 MHz, DCM) 4.32 ppm (s, 3H, N^+*Me*). ^{13}C{^{1}H} -NMR (100 MHz, DCM) δ 38.5 ppm (1C, N^+*Me*). ^{11}B-NMR (128 MHz, DCM) δ -25.4 ppm (d, J = 86.8 Hz, H-*B*). ^{19}F-NMR (400 MHz, DCM): -133.96 (6F), -164.36 (3F), -167.35 (6F) ppm.

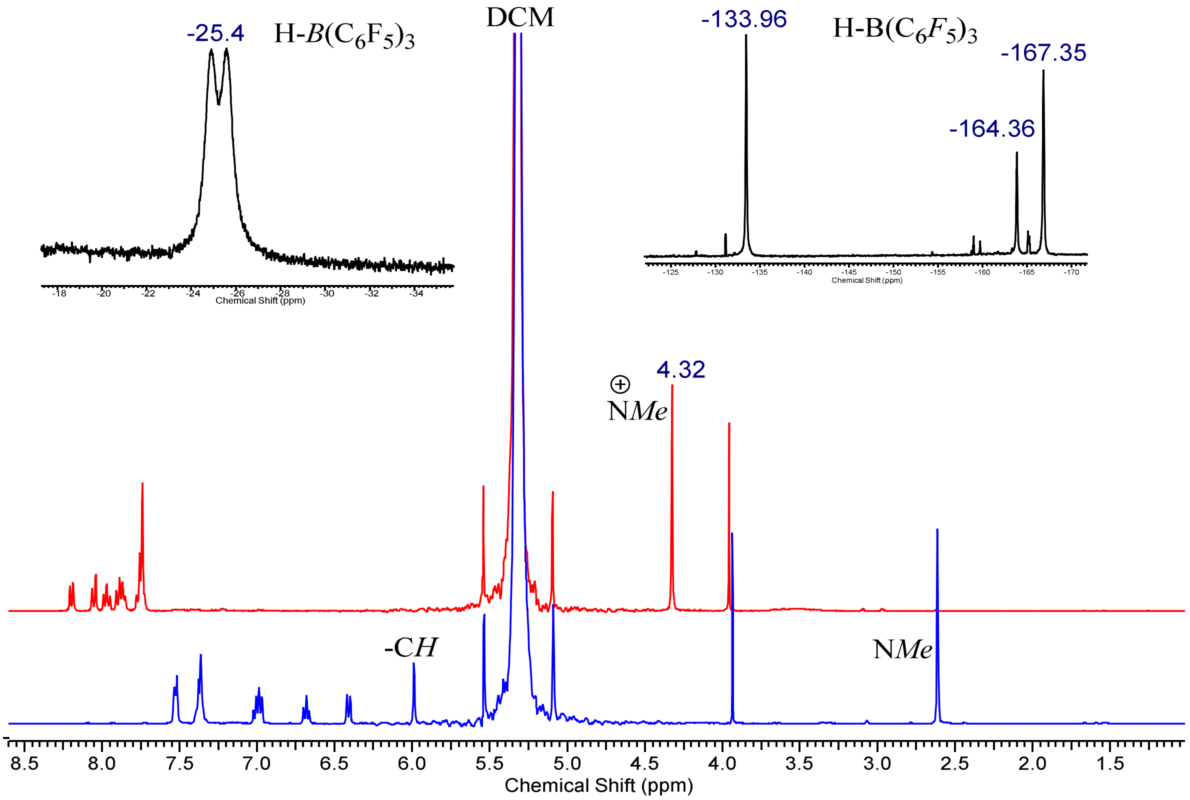

Figure S1. *In situ* ^{1}H, ^{11}B and ^{19}F-NMR spectra of hydride abstraction from *N*-methyl-2-phenyl-benzothiazoline by $B(C_6F_5)_3$ (with capillary inserted containing wet d_6-DMSO): *N*-methyl-2-phenyl-benzothiazoline (blue) and after 5 minutes from $B(C_6F_5)_3$ addition (red and black).

4. Binding interaction study between [2][BArCl] and 2,6-lutidine/water

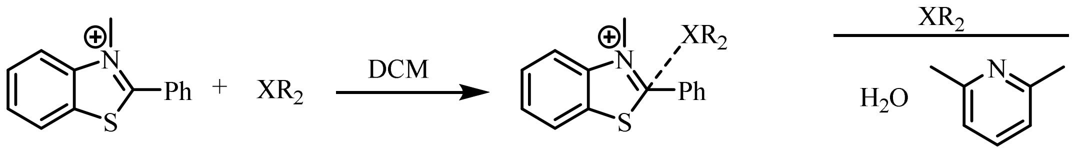

A J. Youngs NMR tube equipped with a DMSO-d_6 capillary was loaded with **[2][BArCl]** (20.0 mg, 0.025 mmol, 1.00 eq.) and dichloromethane (0.5 mL). Then, 2,6-lutidine (3 µL, 0.025 mmol, 1.00 eq.) was added to the solution. Multinuclear NMR spectroscopy revealed no binding interaction. Then, water (1 µL, 0.050 mmol, 2.00 eq.) was added to the solution. Also in this case, no binding interaction or heterolytic O-H cleavage was observed, including after heating the solution at 60°C overnight.

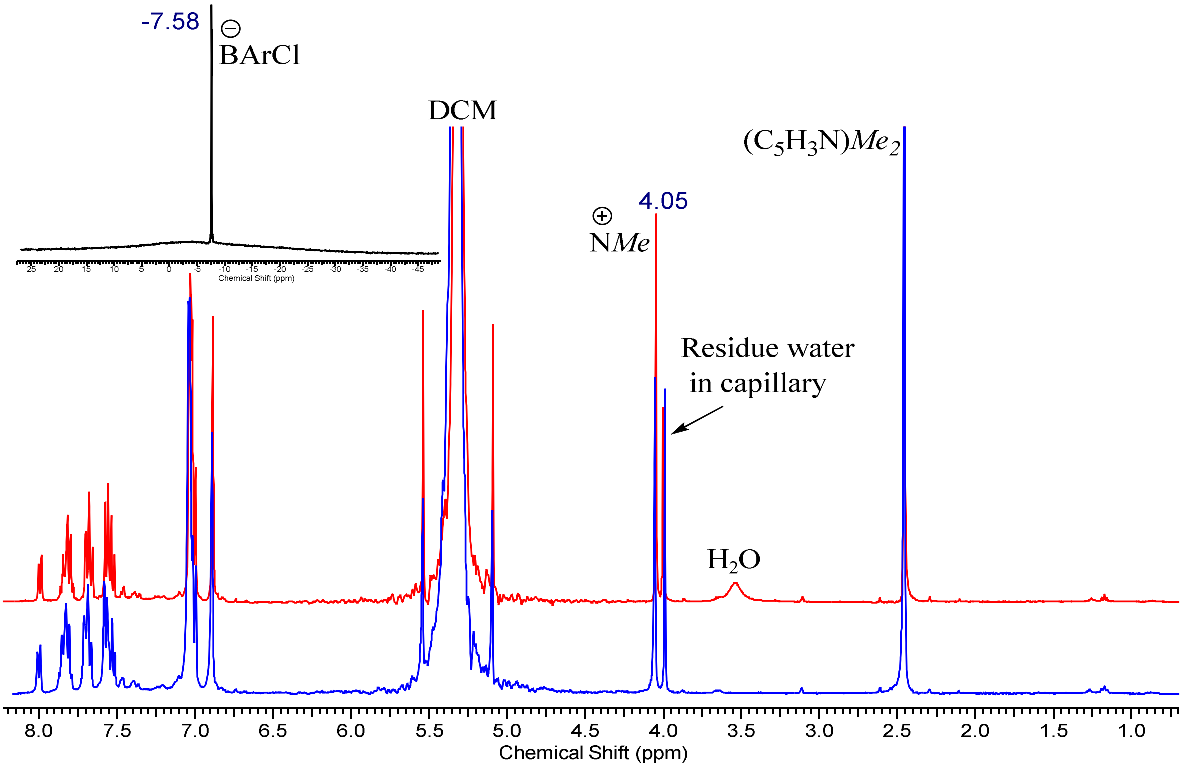

Figure S2. *In situ* ^{1}H and ^{11}B-NMR of binding interaction study (with capillary inserted containing wet d_6-DMSO): **[2][BArCl]** and 2,6-lutidine (blue) and after 20 hours at 60°C after water addition (red and black).

5. Gutmann-Beckett method measurements

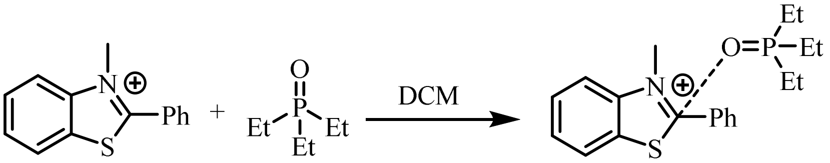

A J. Youngs NMR tube equipped with a DMSO-d_6 capillary was loaded with triethylphosphine oxide (3.0 mg, 0.022 mmol, 1.00 eq.) in dichloromethane (0.5 mL). After initial multinuclear NMR acquisition, **[2][BArCl]** (53.4 mg, 0.065 mmol, 3.00 eq.) was added. The ^{31}P-NMR spectrum of the mixture showed clear broadening and down-field shift of the phosphorus resonance (from 50.4 to 54.8 ppm). The peak at 77.8 ppm is a trace impurity present in the phosphine oxide made more apparent due to the massive decrease in signal-to-noise ratio resulting from the dynamic Lewis acid/base interaction. The overall Δδ was determined to be 4.4 ppm. The ^{1}H-NMR spectrum confirms this weak, reversible interaction, with a small downfield shift of the Et_3PO methylene resonances to δ1.66 ppm (cf. δ1.63 ppm for free Et_3PO in DCM).

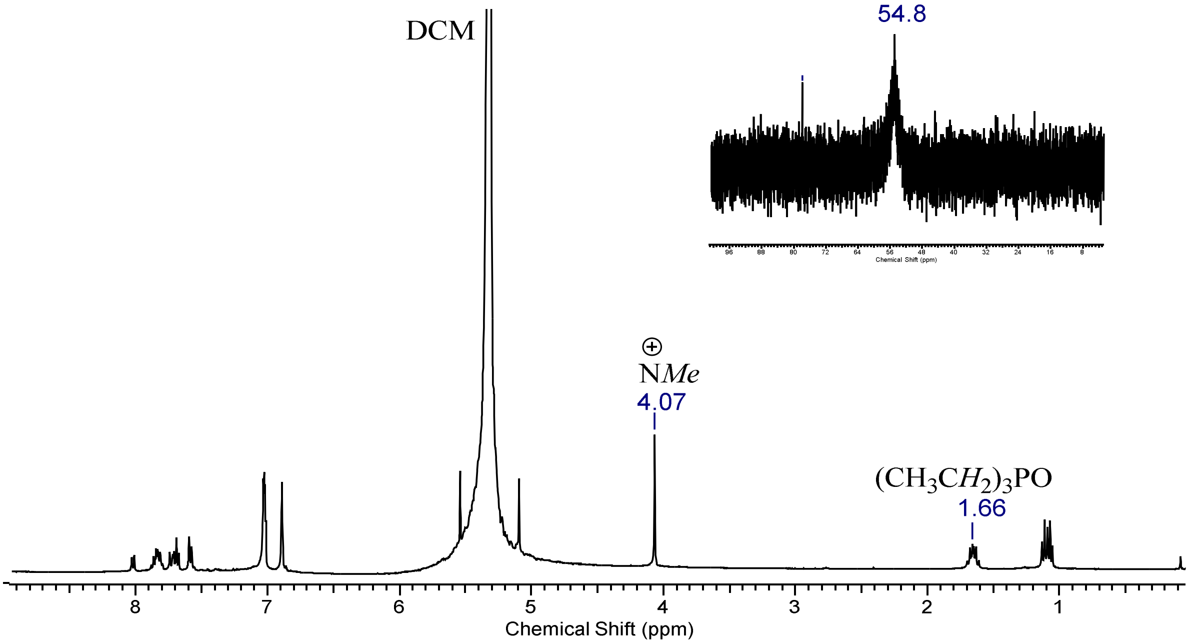

Figure S3. *In situ* ^{1}H and ^{31}P-NMR of Gutmann-Beckett method between Et_3PO and **[2][BArCl]** (with capillary inserted containing wet d_6-DMSO).

6. Acid initiated stepwise reduction of *N*-benzylidene-aniline with *N*-methyl-2-phenyl-benzothiazoline

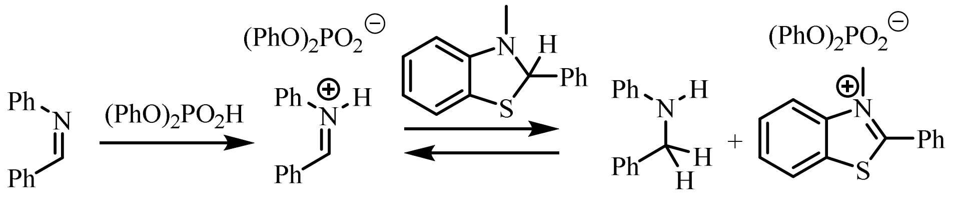

A J. Youngs NMR tube equipped with a DMSO-d_6 capillary was loaded with *N*-benzylidene-aniline (11.0 mg, 0.060 mmol, 1.00 eq) and diphenylphosphoric acid (38.0 mg, 0.150 mmol, 2.50 eq.) in dichloromethane (0.5 mL). After monitoring the initial reaction mixture by multinuclear NMR spectroscopy, *N*-methyl-2-phenyl-benzothiazoline (13.7 mg, 0.060 mmol, 1.00 eq.) was added. The reaction mixture was heated at 60°C for 48 hours and periodically analysed by ^{1}H-NMR spectroscopy. Diagnostic peaks: ^{1}H-NMR (400 MHz, DCM): δ 4.16 (s, 3H, N$^+$*Me*) and 4.18 (s, 2H, PhCH_2N) ppm. GC-MS: m/z calculated for $C_{13}H_{13}N$, 183.1; found 183.1. GC-MS retention times of analyte: 10.50 minutes. MS: m/z calcd for $C_{14}H_{12}NS^+$ 226.3 Found ES$^+$ 226.4; m/z calcd for $C_{12}H_{10}O_4P^-$ 249.2 Found ES$^+$ 249.0

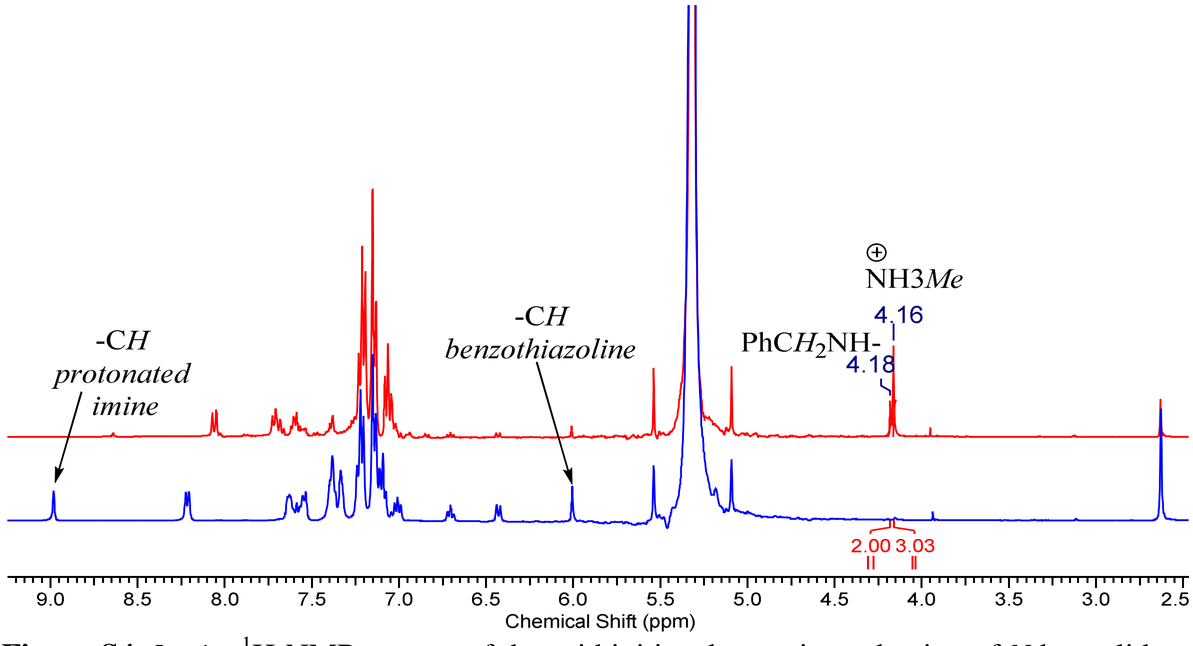

Figure S4. *In situ* ^{1}H-NMR spectra of the acid initiated stepwise reduction of *N*-benzylidene-aniline with *N*-methyl-2-phenyl-benzothiazoline in DCM (with capillary inserted containing wet d_6-DMSO): t=5 min at r.t. (blue), 48 hours at 60°C (red).

7. Attempted H_2 activation experiments

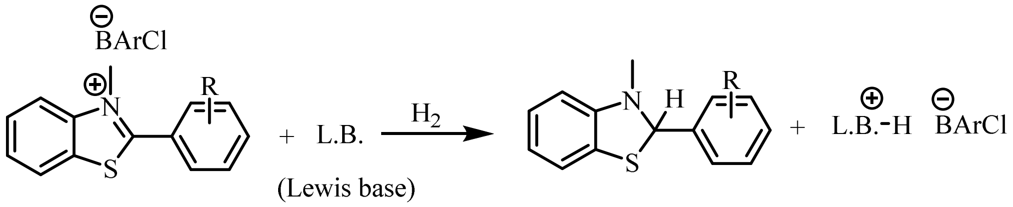

7.1 General procedure for H_2 activation experiments

A J. Young's NMR tube was equipped with a d_6-DMSO capillary, before equimolar amounts of carbon Lewis acid and hindered Lewis base were added and dissolved in dry *o*-DCB (0.5 mL) under an atmosphere of nitrogen. The solution was degassed by freeze-pump-thaw cycles, before being backfilled with H_2 at –196°C (~ 4 bar H_2). The tube was sealed and heated, with the progress of the reaction being periodically checked by multinuclear NMR spectroscopy.

7.2 [2][BArCl] (or [4][BArCl]) with nitrogen based Lewis bases

A selection of nitrogen-based hindered Lewis bases (2,6-lutidine, Proton Sponge® (1,8-bis(dimethylamino)naphthalene, 4-DMAP) were tested with **[2][BArCl]** and **[4][BArCl]** utilising the above experimental protocol, but after heating at 100°C for 16 hours no reaction was observed by NMR spectroscopy.

7.3 [4][BArCl] with phosphorus based Lewis base

(^{t}Bu)$_3$P as Lewis base: Following the general H_2 activation protocol, **[4][BArCl]** (20 mg, 0.023 mmol, 1.00 eq.) and (^{t}Bu)$_3$P (5 mg, 0.023 mmol, 1.00 eq.) were combined before *o*-DCB was added (0.5 mL). The sample was heated at 60 °C for 24 hours, after which no reaction was observed by NMR spectroscopy. After heating the reaction for 24 hours at 100°C analysis by ^{1}H-NMR spectroscopy indicated demethylation of the benzothiazolium had occurred. ^{31}P-NMR spectroscopy displayed three new ^{31}P-resonances (^{31}P-NMR (202 MHz, *o*-DCB/ d_6-DMSO) δ 60.4 (d, J = 160 Hz), 57.9, 48.1), of which two can be assigned as [H-P(^{t}Bu)$_3$]$^+$ (60.4 ppm)[3] and [Me-P(^{t}Bu)$_3$]$^+$ (48.1 ppm).[4] The presence of these two phosphine species was confirmed by mass spectrometry. MS (ESI+) m/z: 203.1 ([H-P(^{t}Bu)$_3$]$^+$), 217.2 ([Me-P(^{t}Bu)$_3$]$^+$). No further products of the reaction were isolated or characterised.

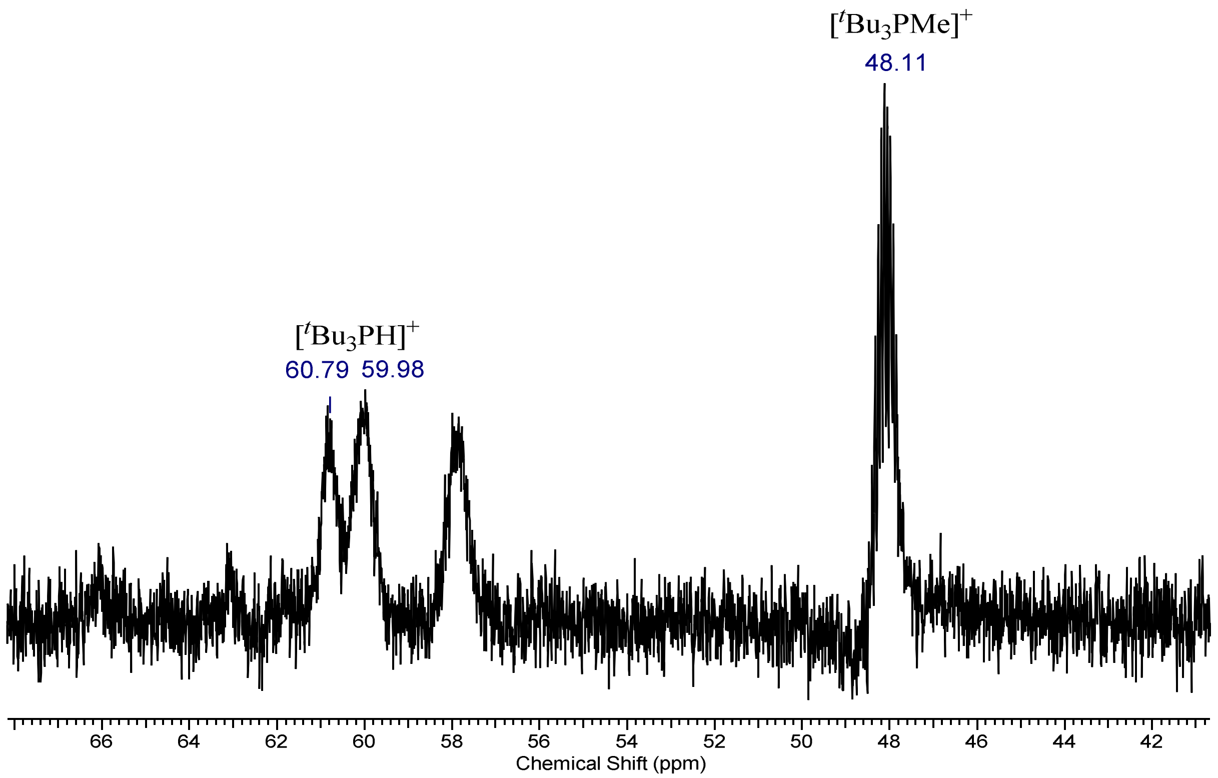

Figure S5. *In situ* ^{31}P-NMR of reaction between **[4][BArCl]** and (^{t}Bu)$_3$P, after heating at 100°C for 24 hours (with capillary inserted containing wet d_6-DMSO).

8. Silane activation: H/D scrambling

H/D scrambling using [2][BPh_4]: $Et_3SiD/PhMe_2SiH$

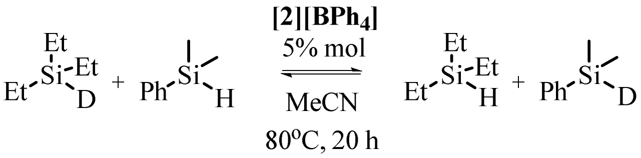

A J. Youngs NMR tube equipped with a DMSO-d_6 capillary was loaded with **[2][BPh_4]** (5.8 mg, 0.011 mmol, 0.05 eq) and acetonitrile (0.5 mL). To the obtained solution, dimethylphenylsilane (34 µL, 0.213 mmol, 1.00 eq.) and Et_3SiD (35 µL, 0.213 mmol, 1.00 eq.) were added. After monitoring the initial reaction mixture by multinuclear NMR spectroscopy, the J. Youngs NMR tube was heated at 80°C for 20 hours. The reaction mixture was then monitored again by ^{1}H, ^{2}D and ^{29}Si{^{1}H}-NMR spectroscopy which revealed the formation of Et_3SiH and $PhMe_2SiD$. Et_3SiH : $PhMe_2SiH$ = 38 : 62. Diagnostic peaks (Et_3SiH): ^{1}H-NMR (400 MHz, MeCN): δ 3.62 (sept, *J* = 3.0 Hz, 1H, Si-*H*) ppm. ^{29}Si{^{1}H}-NMR (81 MHz, MeCN): δ 0.64 (s, *Si*-H) ppm. Diagnostic peaks ($PhMe_2SiD$): ^{2}D-NMR (61 MHz, MeCN): δ 4.63 (s, 1D, Si-*D*) ppm. ^{29}Si{^{1}H}-NMR (81 MHz, MeCN): δ -17.15 (t, *J* = 28.4 Hz, *Si*-D) ppm. The data were in accordance with those reported in the literature.[5] Under identical conditions, the reaction catalysed by **[2][OTf]** gave $PhMe_2SiD$. Et_3SiH : $PhMe_2SiH$ = 10 : 90.

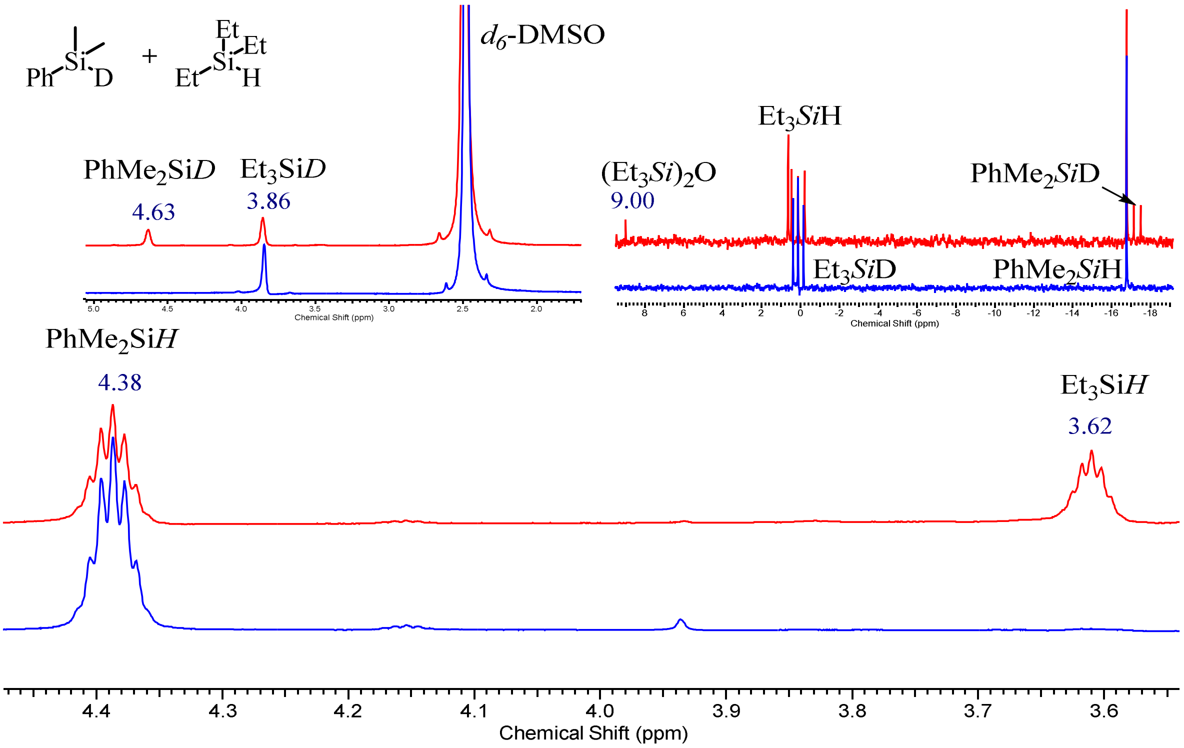

Figure S6. *In situ* ^{1}H, ^{2}D and ^{29}Si{^{1}H}-NMR spectra of the Si-H Si-D scrambling (Et_3SiD: $PhMe_2SiH$) using **[2][BPh_4]** in MeCN (with capillary inserted containing wet d_6-DMSO). t = 5 min at r.t. (blue) *vs* t = 20 hours at 80°C (red).

H/D scrambling using [2][BArCl]: $Et_3SiD/PhMe_2SiH$

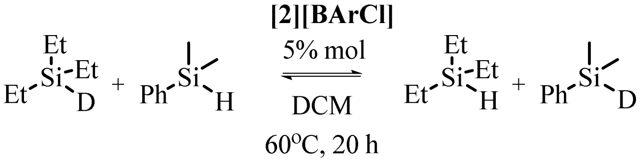

A J. Youngs NMR tube equipped with a DMSO-d_6 capillary was loaded with **[2][BArCl]** (8.8 mg, 0.011 mmol, 0.05 eq) and dichloromethane (0.5 mL). To the obtained solution, dimethylphenylsilane (34 µL, 0.213 mmol, 1.00 eq.) and Et_3SiD (35 µL, 0.213 mmol, 1.00 eq.) were added. After monitoring the initial reaction mixture by multinuclear NMR spectroscopy, the J. Youngs NMR tube was heated at 60°C for 20 hours. The reaction mixture was then monitored again by ^{1}H, ^{2}D and ^{29}Si{^{1}H}-NMR spectroscopy which revealed the formation of Et_3SiH and $PhMe_2SiD$ (trace water from reactants caused the formation of traces of siloxane). Et_3SiH : $PhMe_2SiH$ = 51 : 49. Diagnostic peaks (Et_3SiH): ^{1}H-NMR (400 MHz, DCM): δ 3.64 (sept, J = 3.6 Hz, 1H, Si-*H*) ppm. ^{29}Si{^{1}H}-NMR (81 MHz, DCM): δ -0.16 (s, *Si*-H) ppm. Diagnostic peaks ($PhMe_2SiD$): ^{2}D-NMR (61 MHz, DCM): δ 3.91 (s, 1D, Si-*D*) ppm. ^{29}Si{^{1}H}-NMR (81 MHz, DCM): δ -17.97 (t, J = 28.4 Hz, *Si*-D) ppm. The data were in accordance with those reported in the literature.[5]

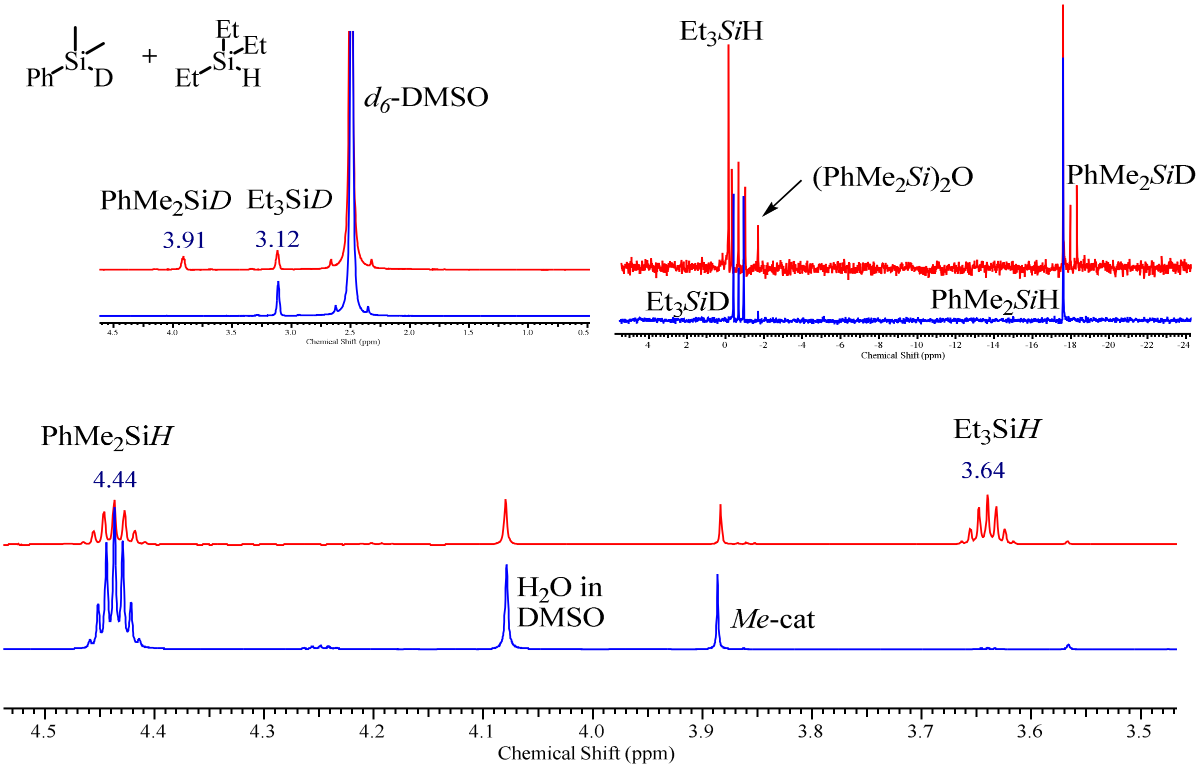

Figure S7. *In situ* ^{1}H, ^{2}D and ^{29}Si{^{1}H}-NMR spectra of the Si-H Si-D scrambling (Et_3SiD: $PhMe_2SiH$) using **[2][BArCl]** in DCM (with capillary inserted containing wet d_6-DMSO). t = 5 min at r.t. (blue) *vs* t = 20 hours at 60°C (red).

H/D scrambling using [2][BArCl]: Et_3SiD/Ph_2MeSiH

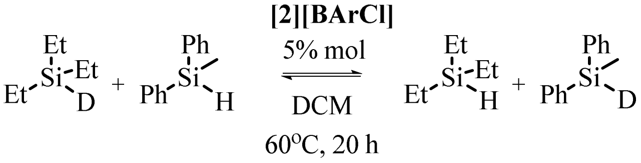

A J. Youngs NMR tube equipped with a DMSO-d_6 capillary was loaded with **[2][BArCl]** (8.8 mg, 0.011 mmol, 0.05 eq) and dichloromethane (0.5 mL). To the obtained solution, methyldiphenylsilane (44 µL, 0.213 mmol, 1.00 eq.) and Et_3SiD (35 µL, 0.213 mmol, 1.00 eq.) were added. After monitoring the initial reaction mixture by multinuclear NMR spectroscopy, the J. Youngs NMR tube was heated at 60°C for 20 hours. The reaction mixture was then monitored again by ^{1}H, ^{2}D and ^{29}Si{^{1}H}-NMR spectroscopy which revealed the formation of Et_3SiH and Ph_2MeSiD. Et_3SiH : Ph_2MeSiH = 14 : 86. Diagnostic peaks (Et_3SiH): ^{1}H-NMR (400 MHz, DCM): δ 3.64 (sept, J = 3.6 Hz, 1H, Si-*H*) ppm. ^{29}Si{^{1}H}-NMR (81 MHz, DCM): δ -0.16 (s, *Si*-H) ppm. Diagnostic peaks (Ph_2MeSiD): ^{2}D-NMR (61 MHz, DCM): δ 4.40 (s, 1D, Si-*D*) ppm. ^{29}Si{^{1}H}-NMR (81 MHz, DCM): δ -18.50 (t, J = 29.3 Hz, *Si*-D) ppm. The data were in accordance with those reported in the literature.[5]

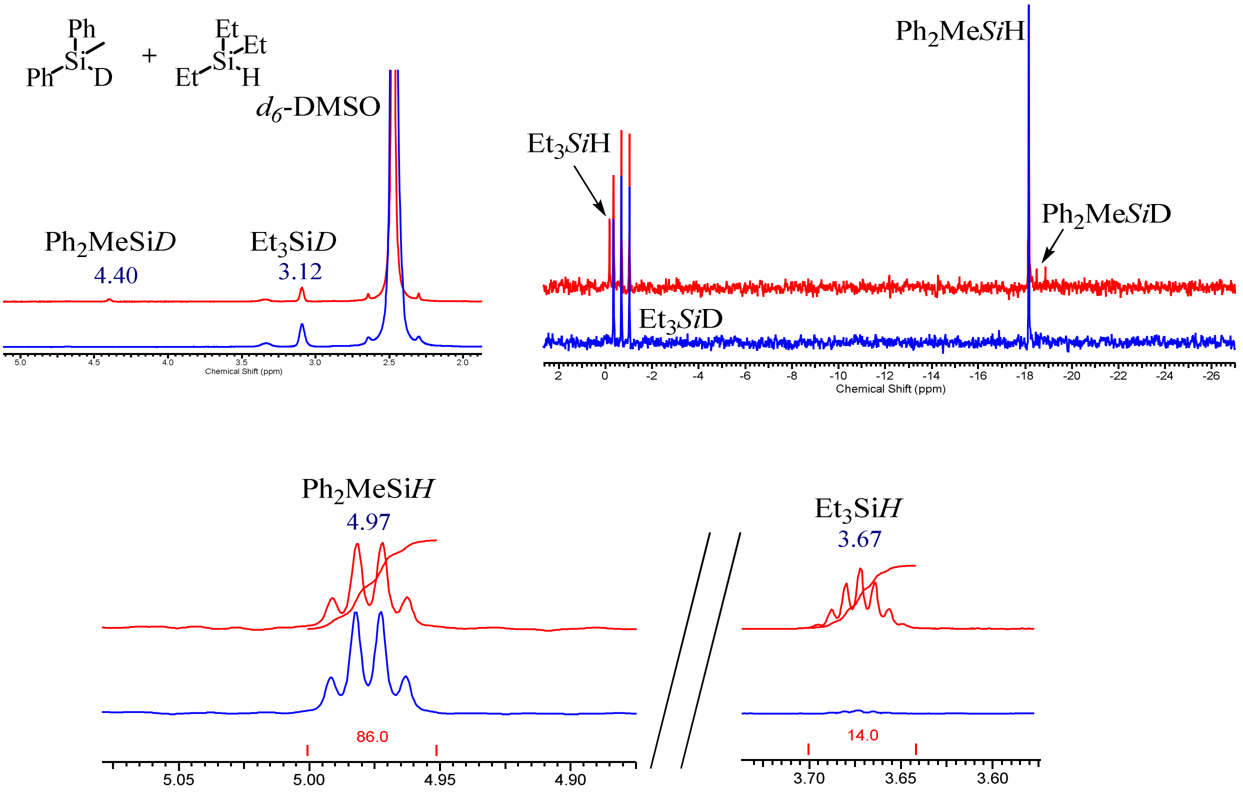

Figure S8. *In situ* ^{1}H, ^{2}D and ^{29}Si{^{1}H}-NMR spectra of the Si-H Si-D scrambling (Et_3SiD: Ph_2MeSiH) using **[2][BArCl]** in DCM (with capillary inserted containing wet d_6-DMSO). t = 5 min at r.t. (blue) *vs* t = 20 hours at 60°C (red).

8.1 Control experiment using $NaBPh_4$

As a control experiment, the H/D scrambling between Et_3SiD and $PhMe_2SiH$ (following the general procedure) was performed with $NaBPh_4$ (5 mol %) in MeCN (20 hours at 80°C), but no reactivity was observed.

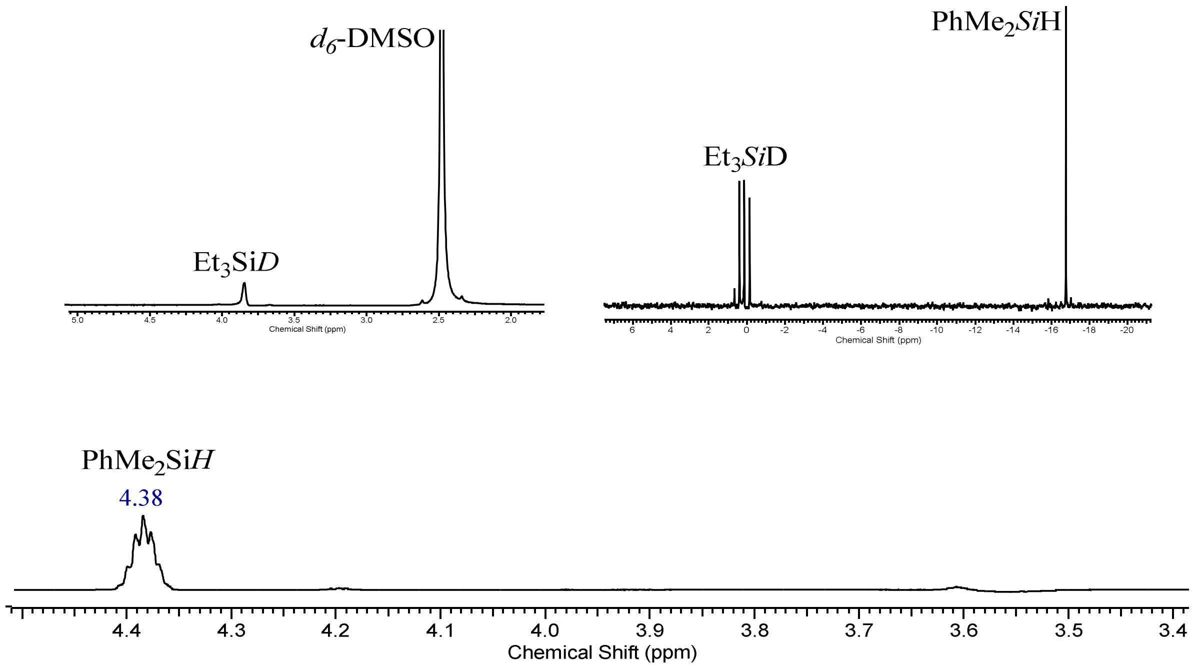

Figure S9. *In situ* ^1H, ^2D and $^{29}Si\{^1H\}$-NMR spectra of the Si-H Si-D scrambling using $NaBPh_4$ in MeCN (with capillary inserted containing wet d_6-DMSO). after 20 hours at 80°C (red).

9. Dehydrosilylation of benzyl alcohol

9.1 General procedure for the dehydrosilylation of benzyl alcohol

A J. Youngs NMR tube equipped with a DMSO-d_6 capillary was loaded with catalyst (0.05 eq.) and the appropriate solvent (0.5 mL). To the obtained solution, benzyl alcohol (1.00 eq.) and dimethylphenylsilane (1.20 eq.) were added. After monitoring the initial reaction mixture by multinuclear NMR spectroscopy, the J. Youngs NMR tube was kept at room temperature for 1 hour. The reaction mixture was then analysed by ^{1}H and ^{29}Si{^{1}H}-NMR spectroscopy. Subsequent addition of mesitylene (25 μL) to the reaction mixture allowed the determination of the NMR yield based on the relative integral of the benzylic protons of the product.

9.2 Benzyloxy-dimethylphenylsilane data

Benzyloxy-dimethylphenylsilane in dichloromethane

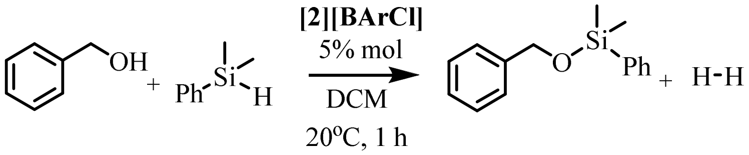

[2][BArCl] (9.8 mg, 0.012 mmol, 0.05 eq.), benzyl alcohol (25 μL, 0.238 mmol, 1.00 eq.) and dimethylphenylsilane (44 μL, 0.286 mmol, 1.20 eq.) in DCM. NMR yield = 94%. Diagnostic peaks: ^{1}H-NMR (400 MHz, DCM): δ 4.72 (s, 2H, -CH_2-), 0.45 (s, 6H, SiMe_2) ppm. ^{29}Si{^{1}H}-NMR (81 MHz, DCM): δ 8.04 ppm. GC-MS: *m/z* calculated for $C_{15}H_{18}OSi$, 242.1; found 242.1. GC-MS retention times of analyte: 12.69 minutes. The data were in accordance with those reported in the literature.[5]

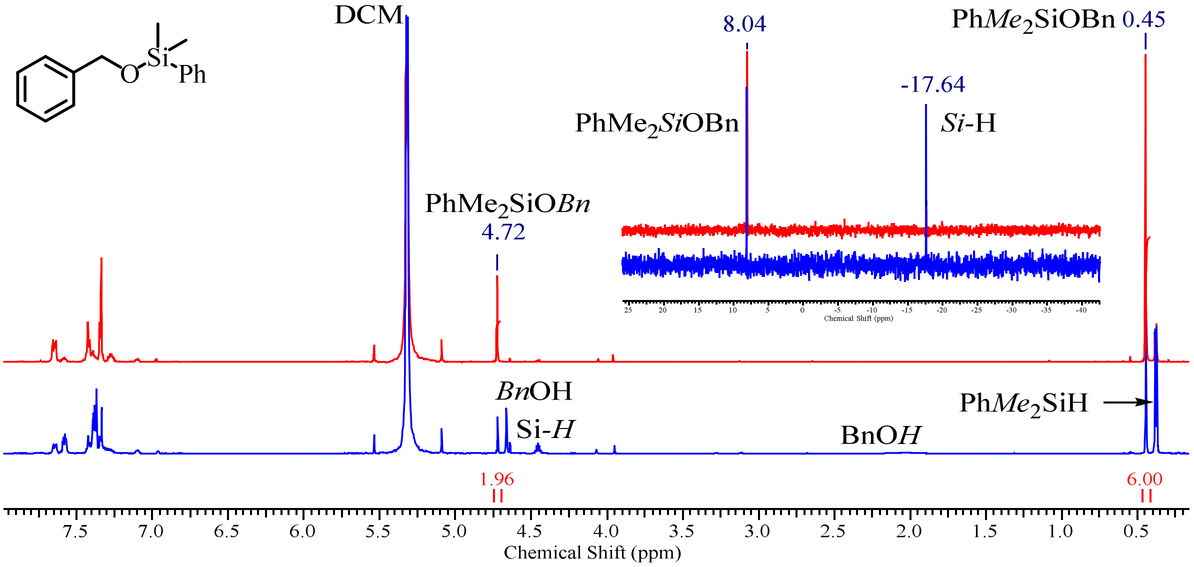

Figure S10. *In situ* ^{1}H and ^{29}Si{^{1}H}-NMR spectra of the dehydrosilylation of benzyl alcohol in DCM using **[2][BArCl]**, before the addition of mesitylene (with capillary inserted containing wet d_6-DMSO). t = 5 min at r.t. (blue) *vs* t = 60 min at r.t. (red).

Benzyloxy-dimethylphenylsilane in acetonitrile

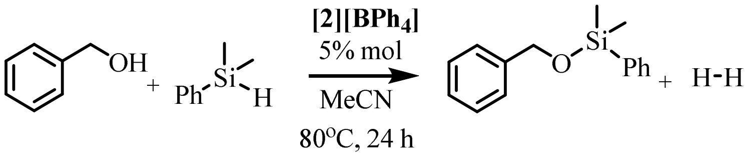

[2][BPh$_4$] (6.5 mg, 0.012 mmol, 0.05 eq), benzyl alcohol (25 μL, 0.238 mmol, 1.00 eq.) and dimethylphenylsilane (44 μL, 0.286 mmol, 1.20 eq.) in MeCN. NMR yield = 76%. Diagnostic peaks: ^{1}H-NMR (400 MHz, MeCN): δ 4.68 (s, 2H, -C*H*$_2$-), 0.38 (s, 6H, Si*Me*$_2$) ppm. ^{29}Si{^{1}H}-NMR(81 MHz, MeCN): δ 8.72 ppm.

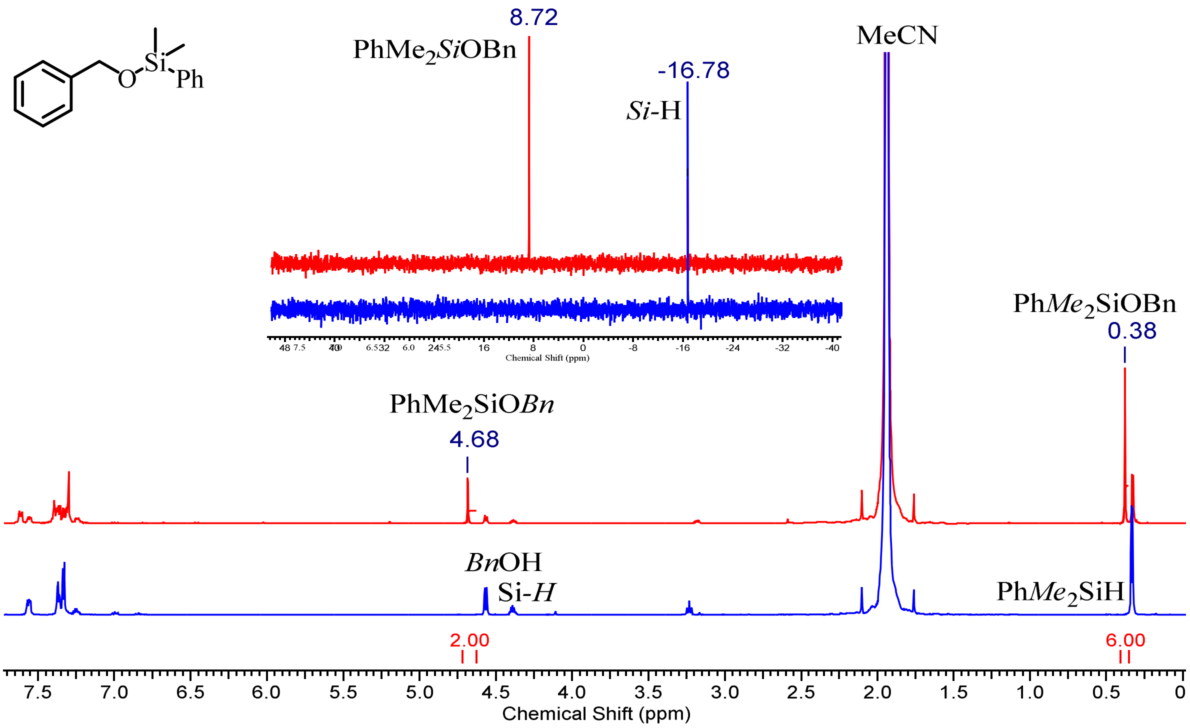

Figure S11. *In situ* ^{1}H and ^{29}Si{^{1}H}-NMR spectra of the dehydrosilylation of benzyl alcohol in MeCN using **[2][BPh$_4$]**, before the addition of mesitylene (with capillary inserted containing wet d_6-DMSO). t = 5 min at r.t. (blue) *vs* t = 24 hours at 80°C (red).

Benzyloxy-dimethylphenylsilane in tetrahydrofuran

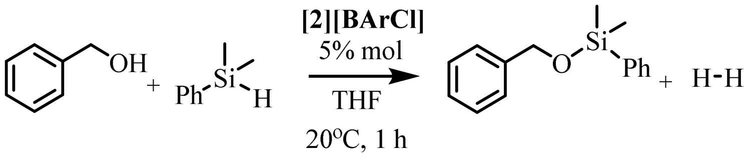

[2][BArCl] (9.8 mg, 0.012 mmol, 0.05 eq), benzyl alcohol (25 μL, 0.238 mmol, 1.00 eq.) and dimethylphenylsilane (35 μL, 0.226 mmol, 0.95 eq.) in THF. NMR yield = 86%. Diagnostic peaks: ^{1}H-NMR (400 MHz, THF): δ 4.67 (s, 2H, -CH_2-), 0.35 (s, 6H, SiMe_2) ppm. ^{29}Si{^{1}H}-NMR (81 MHz, THF): δ 7.57 ppm.

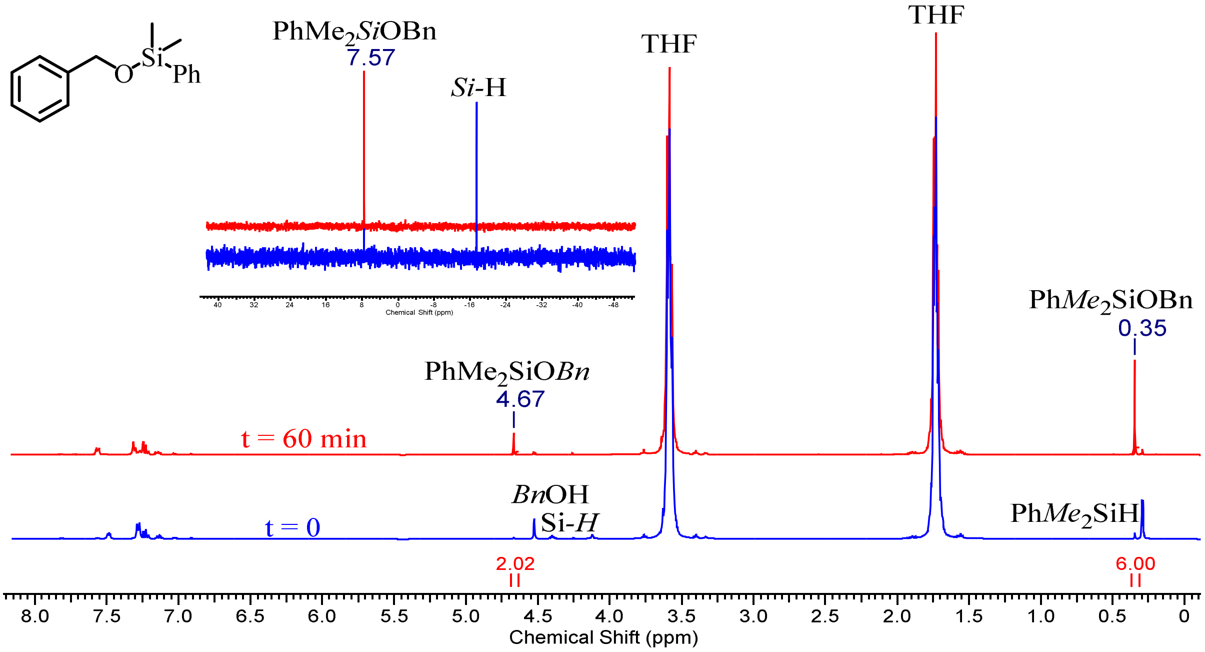

Figure S12. *In situ* ^{1}H and ^{29}Si{^{1}H}-NMR spectra of the dehydrosilylation of benzyl alcohol in THF using **[2][BArCl]**, before the addition of mesitylene (with capillary inserted containing wet d_6-DMSO). t = 5 min at r.t. (blue) *vs* t = 60 min at r.t. (red).

Benzyloxy-dimethylphenylsilane in methyl-*t*-Butyl-ether

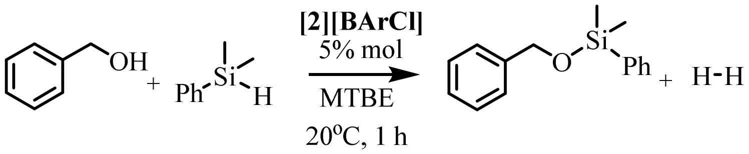

[2][BArCl] (9.8 mg, 0.012 mmol, 0.05 eq), benzyl alcohol (25 μL, 0.238 mmol, 1.00 eq.) and dimethylphenylsilane (44 μL, 0.286 mmol, 1.20 eq.) in MTBE. NMR yield = 94%. Diagnostic peaks: ^{1}H-NMR (400 MHz, MTBE): δ 4.72 (s, 2H, -CH_2-), 0.41 (s, 6H, SiMe_2) ppm. ^{29}Si{^{1}H}-NMR (81 MHz, MTBE): δ 7.50 ppm.

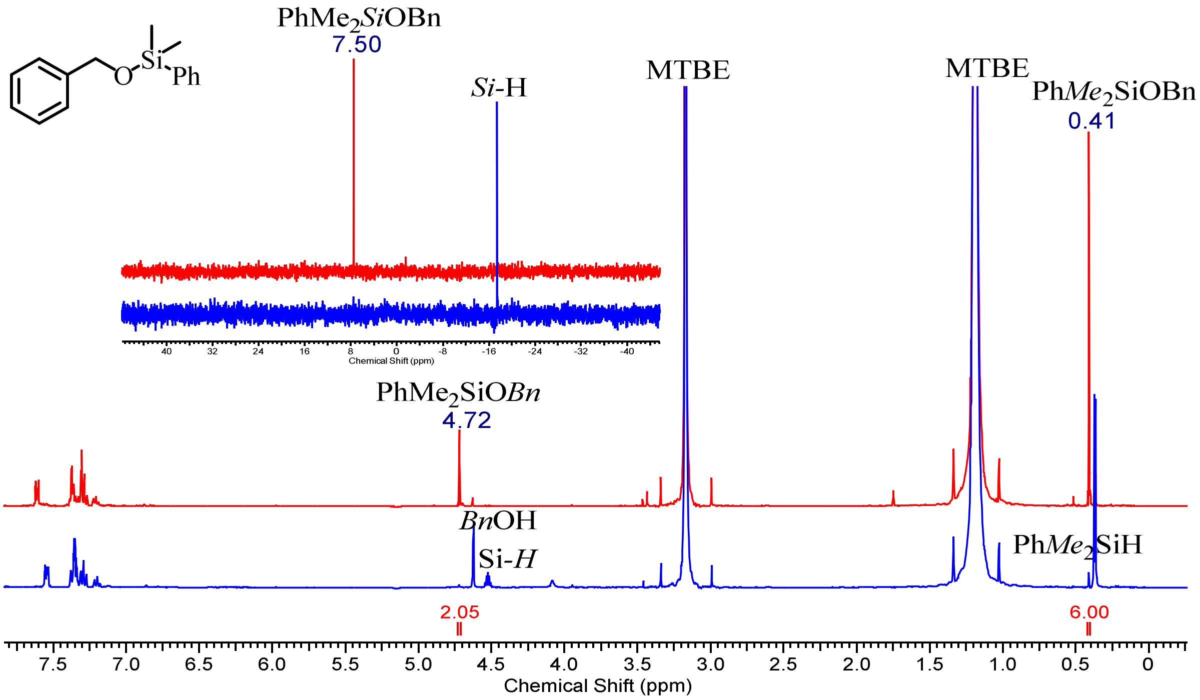

Figure S13. *In situ* ^{1}H and ^{29}Si{^{1}H}-NMR spectra of the dehydrosilylation of benzyl alcohol in MTBE using **[2][BArCl]**, before the addition of mesitylene (with capillary inserted containing wet d_6-DMSO). t = 5 min at r.t. (blue) *vs* t = 60 min at r.t. (red).

Benzyloxy-dimethylphenylsilane in acetonitrile

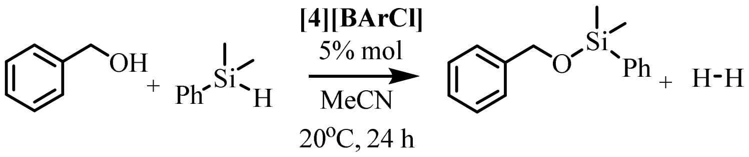

[4][BArCl] (10.4 mg, 0.012 mmol, 0.05 eq.), benzyl alcohol (25 μL, 0.238 mmol, 1.00 eq.) and dimethylphenylsilane (44 μL, 0.286 mmol, 1.20 eq.) in MeCN. NMR yield = 60%. Diagnostic peaks: ^{1}H-NMR (400 MHz, DCM): δ 4.69 (s, 2H, -CH_2-), 0.38 (s, 6H, SiMe_2) ppm. ^{29}Si{^{1}H}-NMR (81 MHz, DCM): δ 8.65 ppm. GC-MS: *m/z* calculated for $C_{15}H_{18}OSi$, 242.1; found 242.1. GC-MS retention times of analyte: 12.69 minutes.

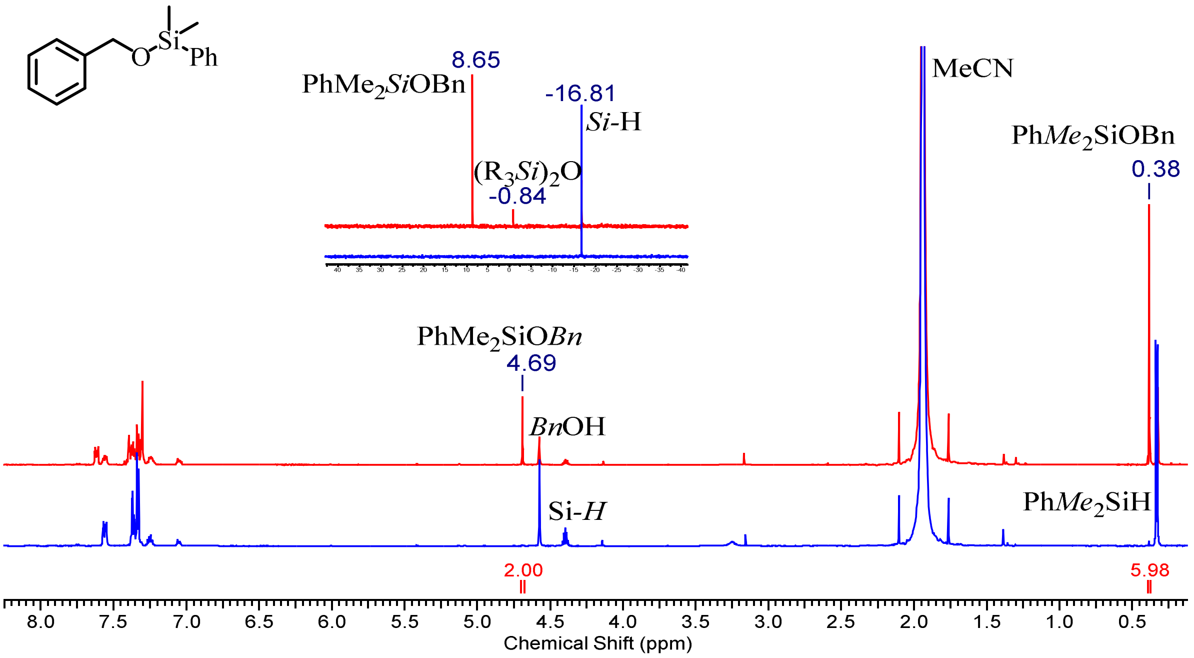

Figure S14. *In situ* ^{1}H and ^{29}Si{^{1}H}-NMR spectra of the dehydrosilylation of benzyl alcohol in DCM using **[2][BArCl]**, before the addition of mesitylene (with capillary inserted containing wet d_6-DMSO). t = 5 min at r.t. (blue) *vs* t = 60 min at r.t. (red).

Benzyloxy-triphenylsilane in dichloromethane

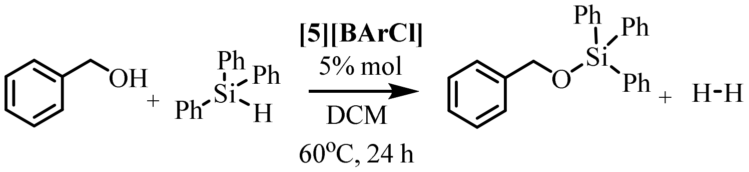

[5][BArCl] (10.4 mg, 0.012 mmol, 0.05 eq.), benzyl alcohol (25 µL, 0.238 mmol, 1.00 eq.) and triphenylsilane (75.9 mg, 0.286 mmol, 1.20 eq.) in DCM. NMR yield = 57%. Diagnostic peaks: ^{1}H-NMR (400 MHz, DCM): δ 4.95 ppm (s, 2H, -CH_2-). ^{29}Si{^{1}H}-NMR (81 MHz, DCM): δ -12.81 ppm. The data were in accordance with those reported in the literature.[5]

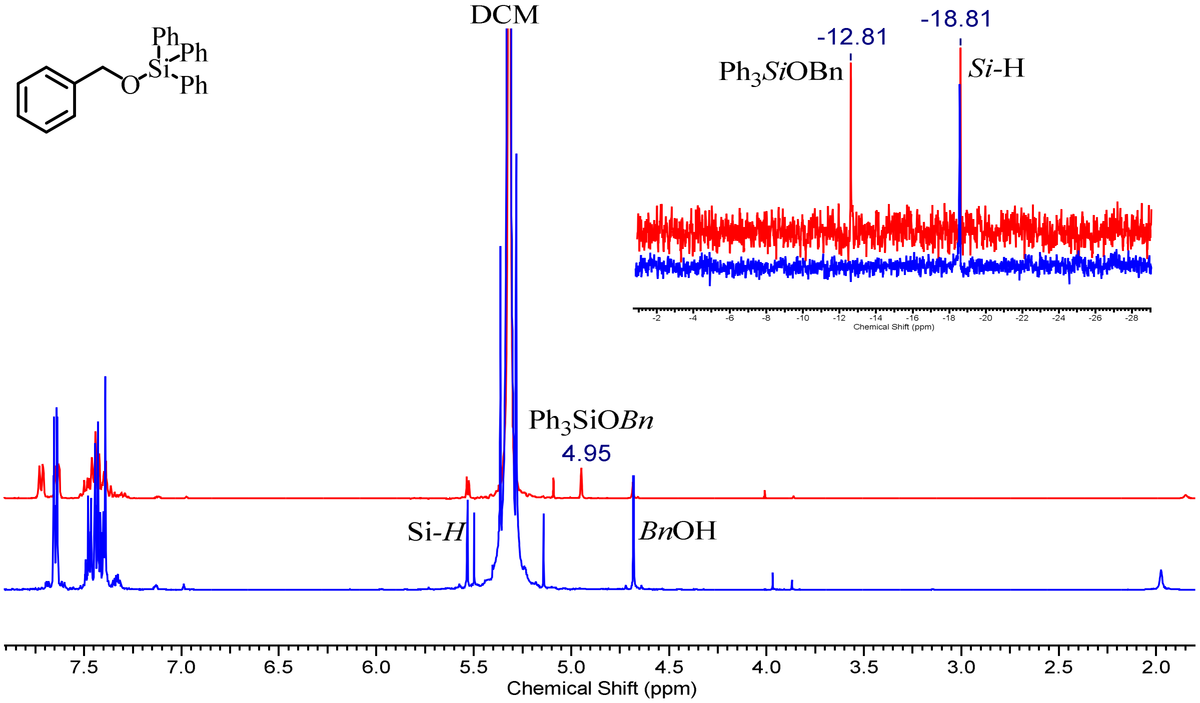

Figure S15. *In situ* ^{1}H and ^{29}Si{^{1}H}-NMR spectra of the dehydrosilylation of benzyl alcohol with Ph_3SiH in DCM using **[5][BArCl]**, before the addition of mesitylene (with capillary inserted containing wet d_6-DMSO). t = 5 min at r.t. (blue) *vs* t = 24 hours at 60°C. (red).

9.3 Control experiments using NaBArCl, $NaBPh_4$ or BPh_3

In the ^{1}H and ^{11}B-NMR spectra of NaBArCl (m/z calcd for $BC_{24}H_{12}Cl_8$ [BArCl] 594.8 Found ES^- 594.9), synthesized according to the literature,[2] very small impurities could be detected (chlorinated arene by-products detected by GC-MS such as 1-bromo-3,5-dichlorobenzene and tetrachlorinated biphenyls). $NaBPh_4$ was purchased from commercial sources and used as received.

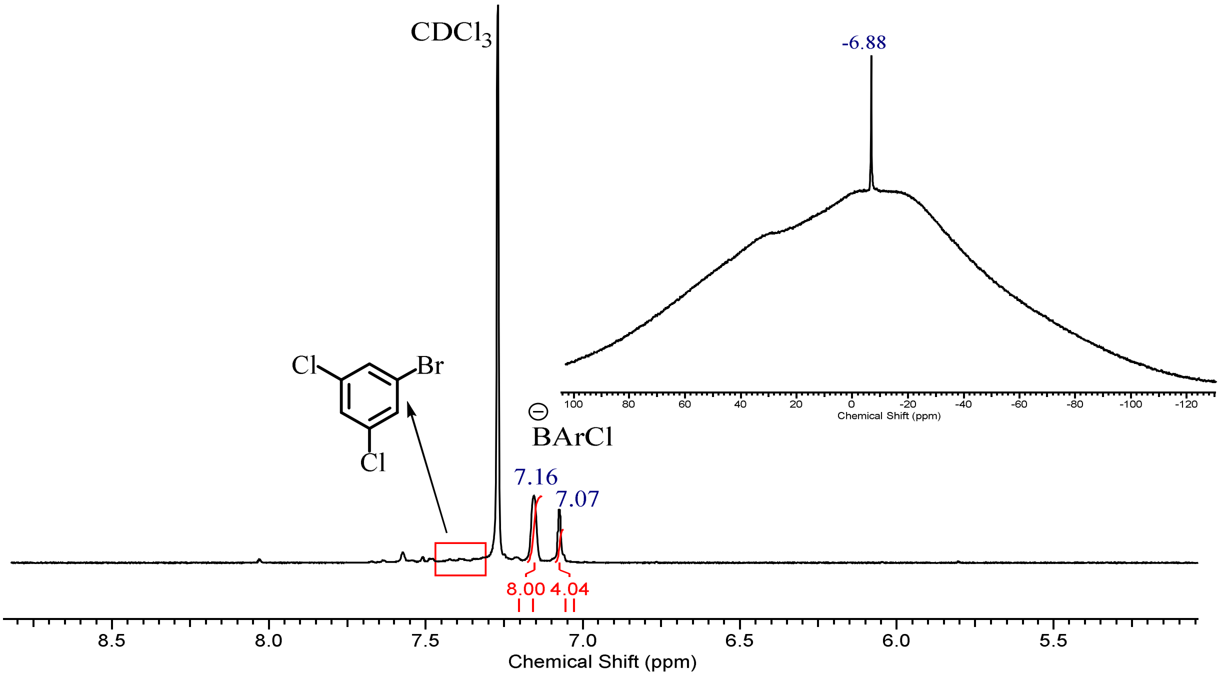

Figure S16. ^{1}H and ^{11}B-NMR spectra of NaBArCl in $CDCl_3$.

As a control experiment, the dehydrosilylation of benzyl alcohol with Ph_2MeSiH (following the general procedure) was performed with $NaBPh_4$ and NaBArCl as potential source of catalyst, but no reaction was observed.

$NaBPh_4$ (4.1 mg, 0.012 mmol, 0.05 eq.), benzyl alcohol (25 µL, 0.238 mmol, 1.00 eq.) and dimethylphenylsilane (44 µL, 0.286 mmol, 1.20 eq.) in MeCN.

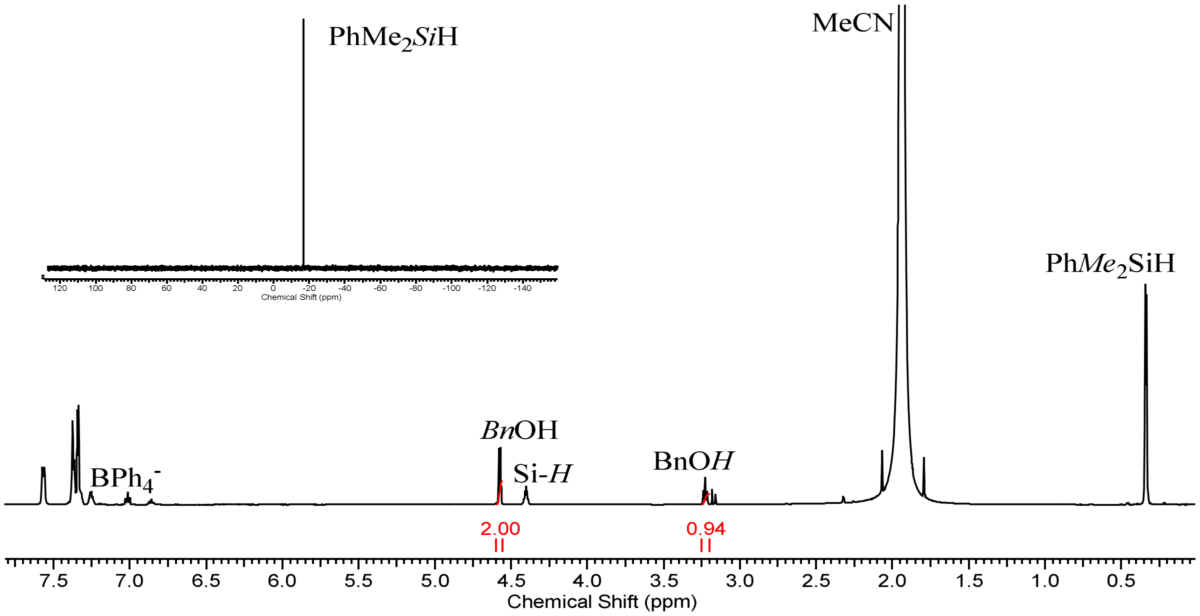

Figure S17. *In situ* ^{1}H and ^{29}Si{^{1}H}-NMR spectra of the attempted dehydrosilylation of benzyl alcohol with $PhMe_2SiH$ in MeCN using $NaBPh_4$, after 24 hours at 80°C (with capillary inserted containing wet d_6-DMSO).

NaBArCl (7.3 mg, 0.012 mmol, 0.05 eq.), benzyl alcohol (25 µL, 0.238 mmol, 1.00 eq.) and dimethylphenylsilane (44 µL, 0.286 mmol, 1.20 eq.) in MeCN.

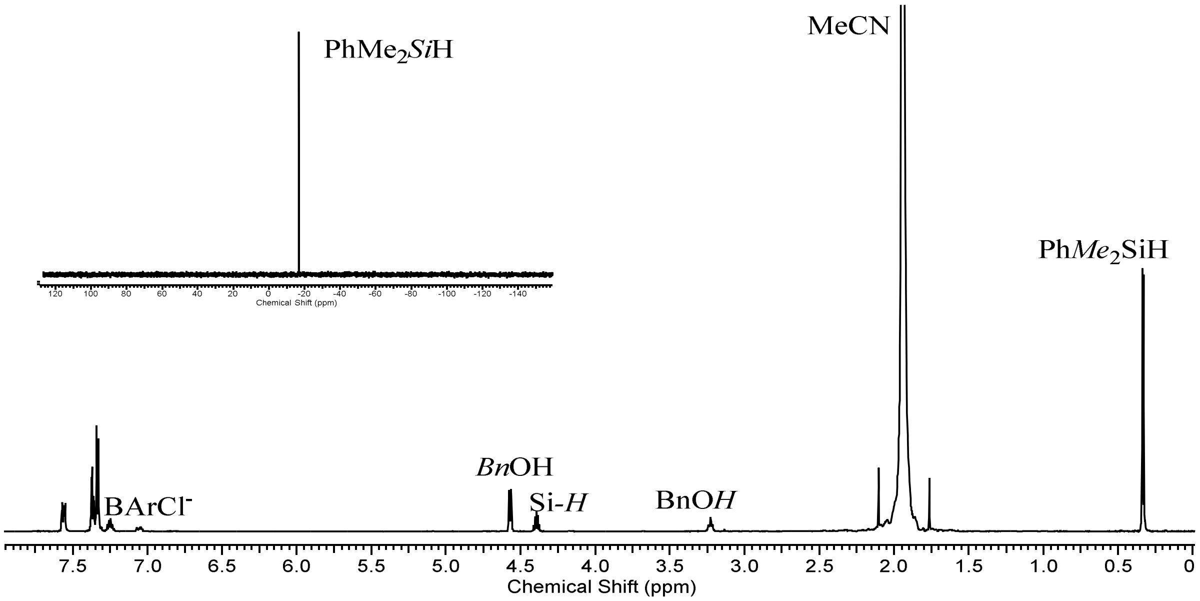

Figure S18. *In situ* ^{1}H and ^{29}Si{^{1}H}-NMR spectra of the attempted dehydrosilylation of benzyl alcohol with $PhMe_2SiH$ in MeCN using NaBArCl, after 24 hours at r.t. (with capillary inserted containing wet d_6-DMSO). No change was observed upon heating at 80°C for 6 hours.

To confirm that anion decomposition would lead to an active catalyst under these conditions, the same dehydrosilylation reaction was performed using BPh_3 as catalyst (40% conversion).

BPh_3 (3.0 mg, 0.012 mmol, 0.05 eq.), benzyl alcohol (25 µL, 0.238 mmol, 1.00 eq.) and dimethylphenylsilane (44 µL, 0.286 mmol, 1.20 eq.) in MeCN.

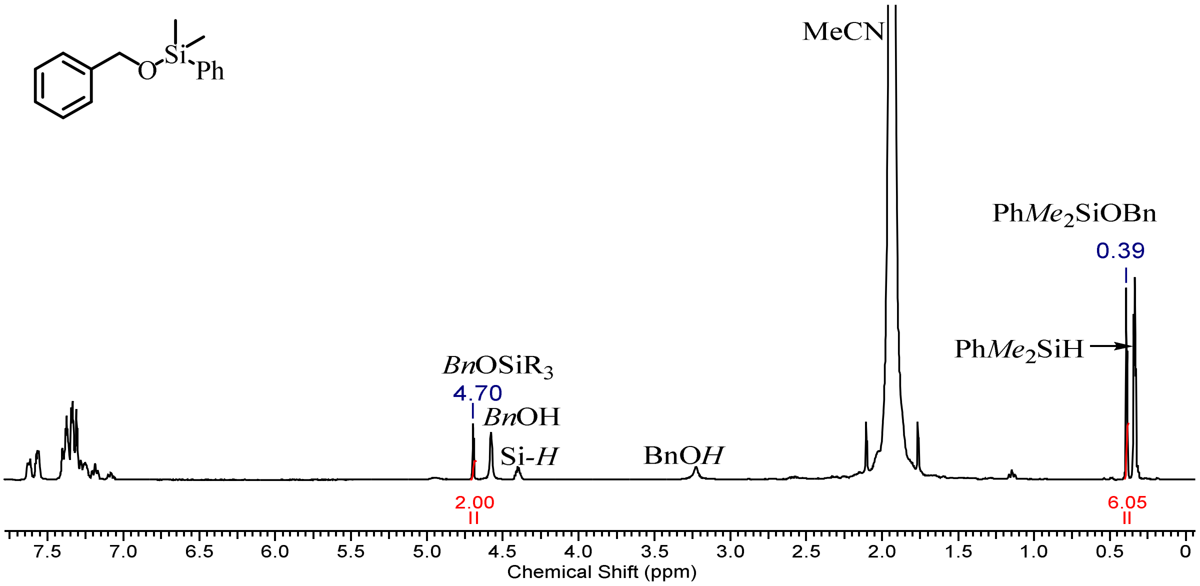

Figure S19. *In situ* ^{1}H-NMR spectrum of the dehydrosilylation of benzyl alcohol with $PhMe_2SiH$ in MeCN using BPh_3, after 24 hours at 80°C (with capillary inserted containing wet d_6-DMSO).

9.4. Dehydrosilylation of phenol

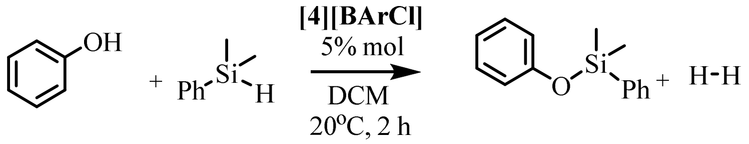

A J. Youngs NMR tube equipped with a DMSO-d_6 capillary was loaded with **[4][BArCl]** (11.1 mg, 0.013 mmol, 0.05 eq) and dichloromethane (0.5 mL). To this solution, phenol (25 mg, 0.255 mmol, 1.00 eq.) and dimethylphenylsilane (48 μL, 0.306 mmol, 1.20 eq.) were added. After monitoring the initial reaction mixture by multinuclear NMR spectroscopy, the J. Youngs NMR tube was kept at room temperature for 2 hours. The reaction mixture was then analysed by ^{1}H and $^{29}Si\{^{1}H\}$-NMR spectroscopy. Subsequent addition of mesitylene (25 μL) to the reaction mixture allowed the determination of the NMR yield based on the relative integral of the methyl protons of the product. NMR yield = 76%. Diagnostic peaks: ^{1}H-NMR (400 MHz, DCM): δ 0.54 (s, 6H, -Si*Me*$_2$) ppm. $^{29}Si\{^{1}H\}$-NMR (81 MHz, DCM): δ 7.67 ppm. The data were in accordance with those reported in the literature.[5]

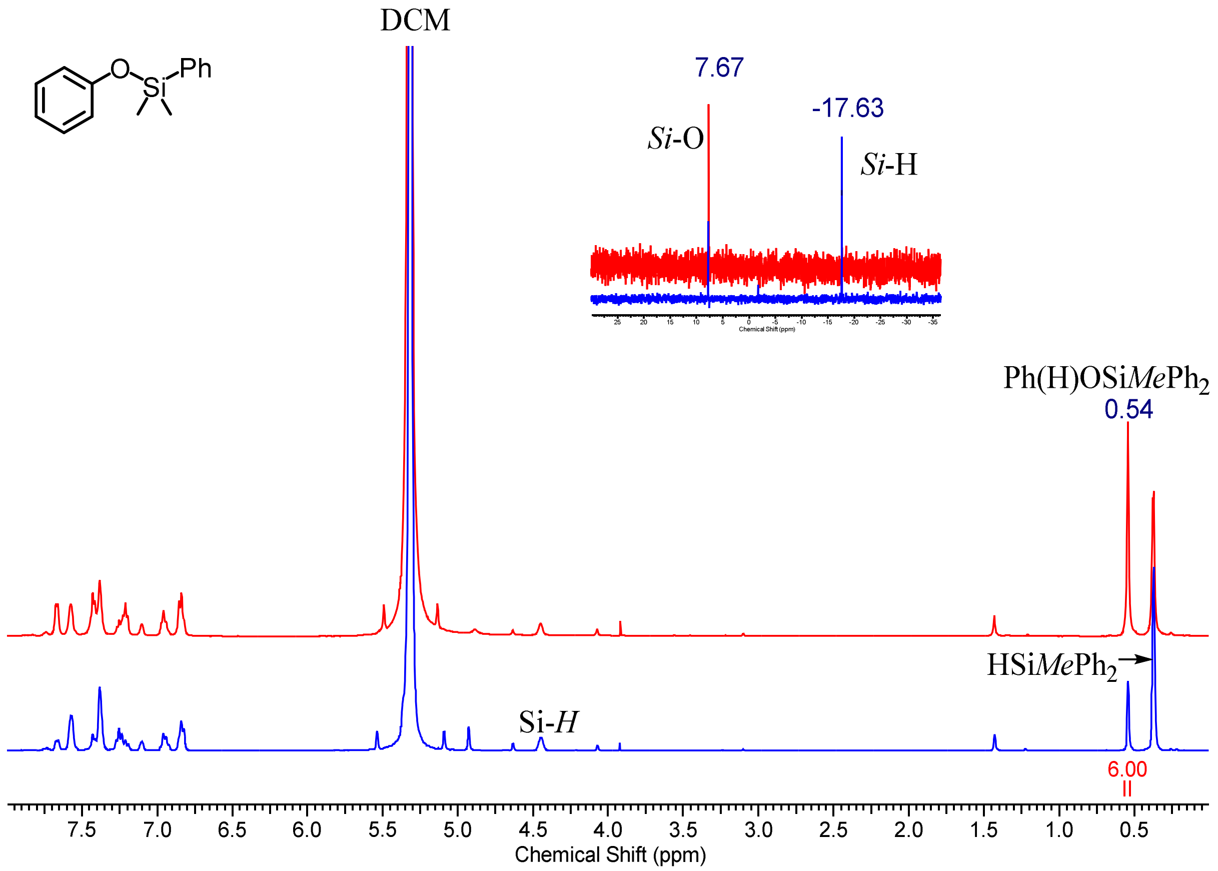

Figure S20. *In situ* ^{1}H and $^{29}Si\{^{1}H\}$-NMR spectra of the dehydrosilylation of phenol with $PhMe_2SiH$ in DCM, before the addition of mesitylene (with capillary inserted containing wet d_6-DMSO). t = 5 min at r.t. (blue) *vs* t = 2 hours at r.t. (red).

10. Hydrosilylation of aldehydes and ketones

10.1 General procedure for hydrosilylation of benzaldehyde/acetophenone

A J. Youngs NMR tube equipped with a DMSO-d_6 capillary was loaded with catalyst (0.05 eq) and DCM (0.5 mL). To this solution, benzaldehyde or acetophenone (1.00 eq.) and dimethylphenylsilane (1.20 eq.) were added. After monitoring the initial reaction mixture by multinuclear NMR spectroscopy, the J. Youngs NMR tube was kept at room temperature for 1 hour and the reaction mixture was then analysed by ^{1}H and ^{29}Si{^{1}H}-NMR spectroscopy. Subsequent addition of mesitylene (25 µL) to the reaction mixture allowed the determination of the NMR yields based on the relative integral of the benzylic protons of the product.

10.2 Alkoxysilane data

Benzyloxy-dimethylphenylsilane

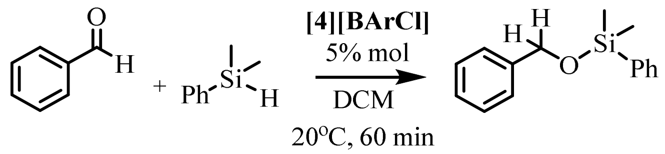

[4][BArCl] (10.5 mg, 0.012 mmol, 0.05 eq), benzaldehyde (25 µL, 0.241 mmol, 1.00 eq.) and dimethylphenylsilane (45 µL, 0.289 mmol, 1.20 eq.) in dichloromethane (0.5 mL). NMR yield = 90%. Diagnostic peaks: ^{1}H-NMR (400 MHz, DCM): δ 4.72 (s, 2H, -CH_2-), 0.44 (s, 6H, SiMe_2) ppm. ^{29}Si{^{1}H}-NMR (81 MHz, DCM): δ 8.08 ppm. The data were in accordance with those reported in the literature.[5]

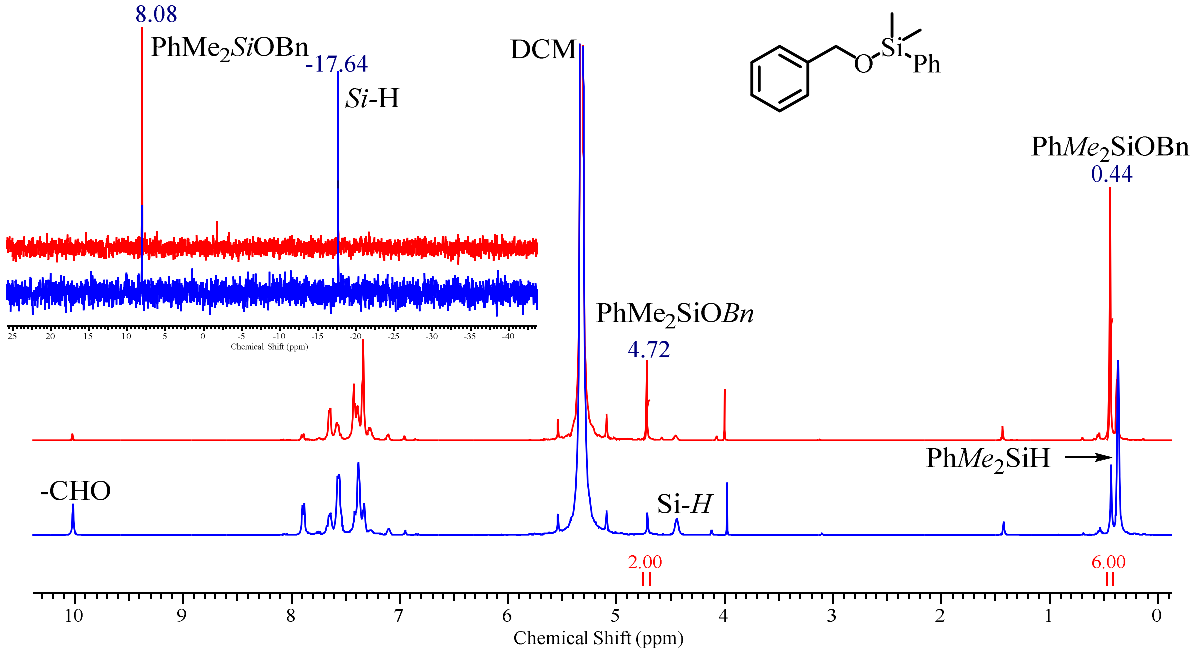

Figure S21. *In situ* ^{1}H and ^{29}Si{^{1}H}-NMR spectra of the hydrosilylation of benzaldehyde with **[4][BArCl]** in DCM, before the addition of mesitylene (with capillary inserted containing wet d_6-DMSO). t = 5 min at r.t. (blue) *vs* t = 60 min at r.t. (red).

(1-phenylethoxy)-dimethylphenylsilane

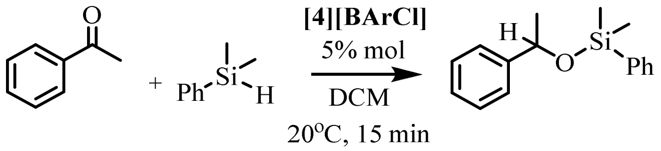

[4][BArCl] (9.2 mg, 0.010 mmol, 0.05 eq), acetophenone (25 µL, 0.210 mmol, 1.00 eq.) and dimethylphenylsilane (39 µL, 0.252 mmol, 1.20 eq.) in dichloromethane (0.5 mL). NMR yield = >99%. Diagnostic peaks: ^{1}H-NMR (400 MHz, DCM): δ 4.89 (q, J = 6.4 Hz, 1H, C*H*), 1.44 (d, J = 6.4 Hz, 3H, -CH-*Me),* 0.35 (s, 3H, -Si*Me*), 0.33 (s, 3H, -Si*Me'*) ppm. ^{29}Si{^{1}H}-NMR (81 MHz, DCM): δ 5.85 ppm. GC-MS: *m/z* calculated for $C_{16}H_{20}OSi$, 256.1; found 241.1 $[M-CH_3]^+$. GC-MS retention times of analyte: 10.18 minutes. The data were in accordance with those reported in the literature.[6]

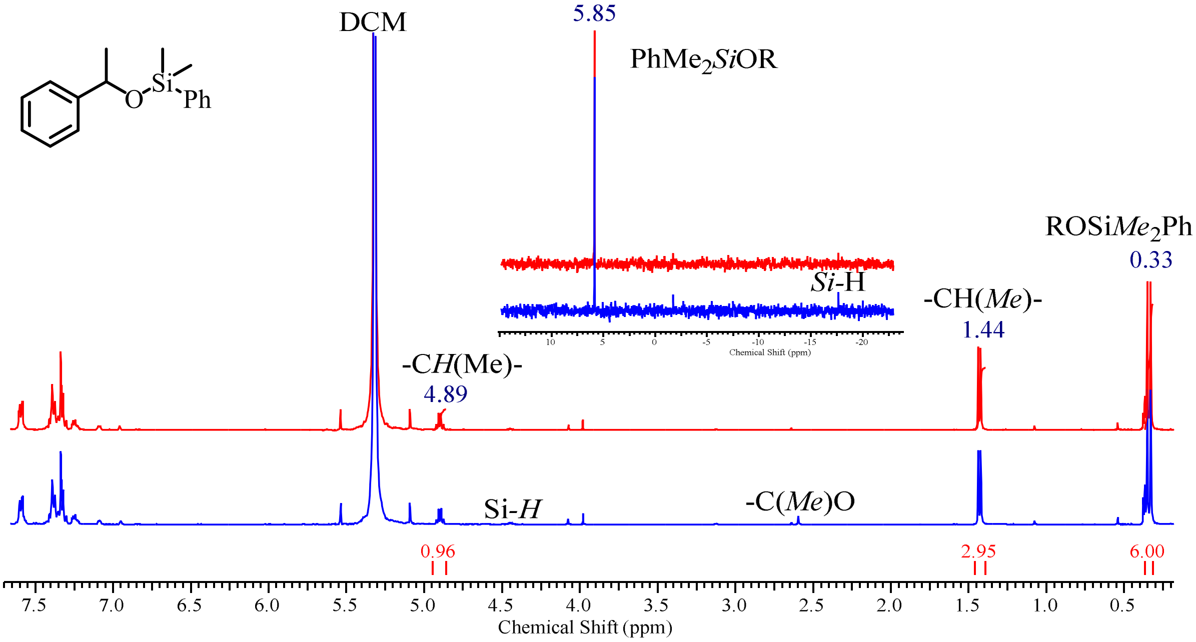

Figure S22. *In situ* ^{1}H and ^{29}Si{^{1}H}-NMR spectra of the hydrosilylation of acetophenone with **[4][BArCl]** in DCM, before the addition of mesitylene (with capillary inserted containing wet d_6-DMSO). t = 5 min at r.t. (blue) *vs* t = 15 min at r.t. (red).

10.3 Control experiment using NaBArCl

As a control experiment, the hydrosilylation of acetophenone with Ph_2MeSiH (following the general procedure) was performed with NaBArCl (5 mol %) in DCM (60 min at r.t.), but no reactivity was observed.

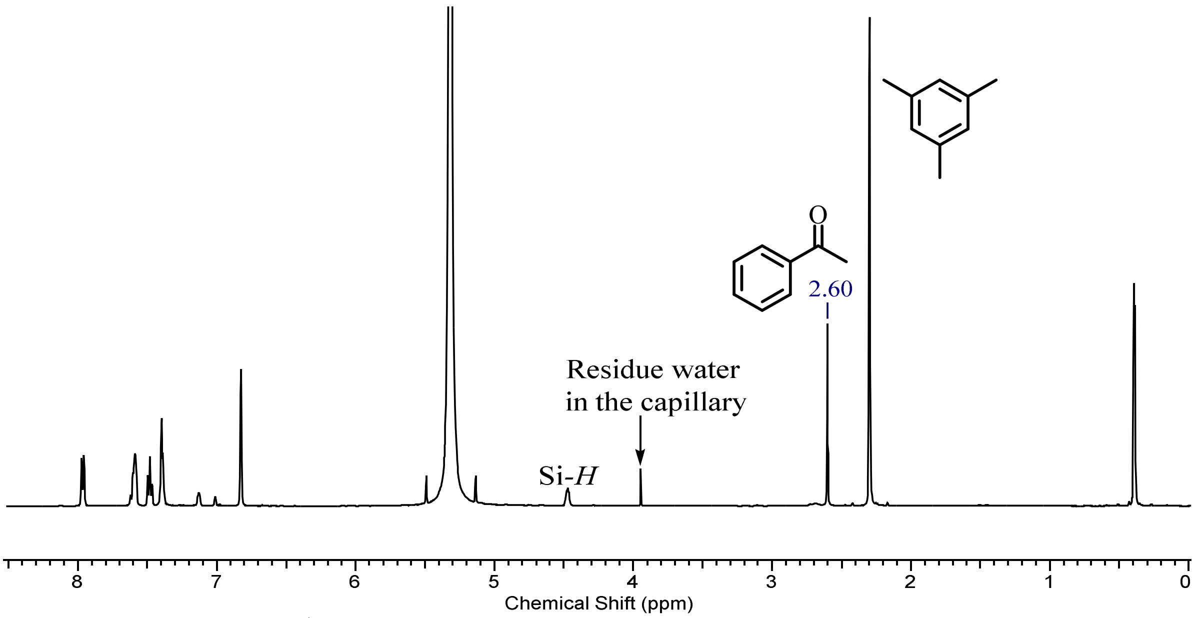

Figure S23. *In situ* ^{1}H-NMR spectrum of the hydrosilylation of acetophenone with Ph_2MeSiH in DCM using NaBArCl, after 60 min at r.t., after the addition of mesitylene (with capillary inserted containing wet d_6-DMSO).

11. Hydrosilylation of imines

11.1 General procedure for hydrosilylation of imines

A J. Youngs NMR tube equipped with a DMSO-d_6 capillary was loaded with **[4][BArCl]** (0.05 eq) and *o*-dichlorobenzene (0.5 mL). To this solution, the imine (1.00 eq.) and silane (1.20 eq.) were added. After monitoring the initial reaction mixture by multinuclear NMR spectroscopy, the reaction was heated at 100°C from 3 to 44 hours and monitored periodically by ^{1}H and ^{29}Si{^{1}H}-NMR spectroscopy. Subsequent addition of mesitylene (25 μL) to the reaction mixture allowed the determination of the NMR yield based on the relative integral of the benzylic proton of the product.

11.2 *N*-silyl-amine data

N-Benzyl-*N*-dimethylphenylsilyl-*t*-butylamine

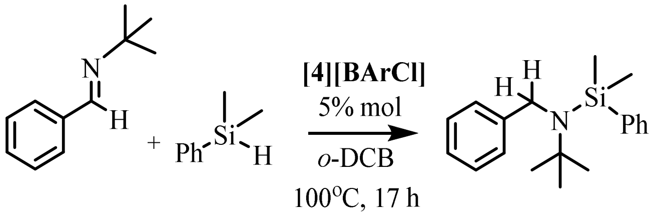

[4][BArCl] (7.0 mg, 0.008 mmol, 0.05 eq), *N*-benzylidene-*t*-butylamine (30 μL, 0.160 mmol, 1.00 eq.) and dimethylphenylsilane (30 μL, 0.193 mmol, 1.20 eq.) in *o*-dichlorobenzene (0.5 mL). NMR yield = 76%. Diagnostic peaks: ^{1}H-NMR (400 MHz, *o*-DCB): δ 4.44 (s, 2H, -CH_2-), 1.28 (s, 9H, -*tBu*), 0.60 (s, 6H, -SiMe_2) ppm. ^{29}Si{^{1}H}-NMR (81 MHz, *o*-DCB): δ -4.56 ppm. The data were in accordance with those reported in the literature.[5]

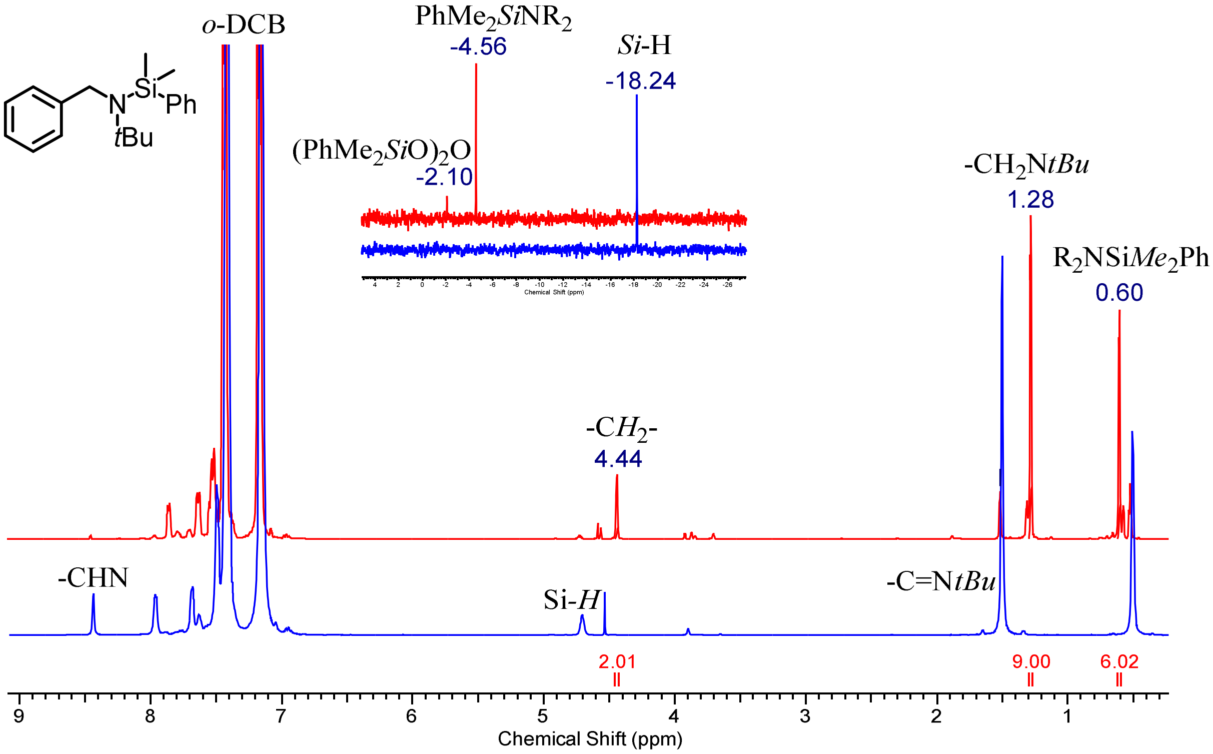

Figure S24. *In situ* ^{1}H and ^{29}Si{^{1}H}-NMR spectra of the hydrosilylation of *N*-benzylidene *t*-butylamine with PhMe$_2$SiH in *o*-DCB, before the addition of mesitylene (with capillary inserted containing wet d_6-DMSO). t = 5 min at r.t. (blue) *vs* t = 17 hours at 100°C (red).

N-Benzyl-*N*-methylphenylsilyl-*t*-butylamine

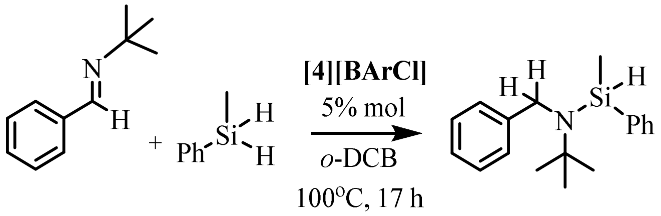

[4][BArCl] (10.8 mg, 0.012 mmol, 0.05 eq), *N*-benzylidene-*t*-butylamine (45 μL, 0.248 mmol, 1.00 eq.) and methylphenylsilane (42 μL, 0.297 mmol, 1.20 eq.) in *o*-dichlorobenzene (0.5 mL). NMR yield = 98%. Diagnostic peaks: ^{1}H-NMR (400 MHz, *o*-DCB): δ 5.40 (q, *J* = 3.6 Hz, 1H, -Si*H*), 4.38 (s, 2H, -CH_2-), 1.33 (s, 9H, -*tBu),* 0.62 (d, *J* = 3.6 Hz, 3H, -Si*Me*) ppm. ^{29}Si{^{1}H}-NMR (81 MHz, *o*-DCB): δ -15.29 ppm.

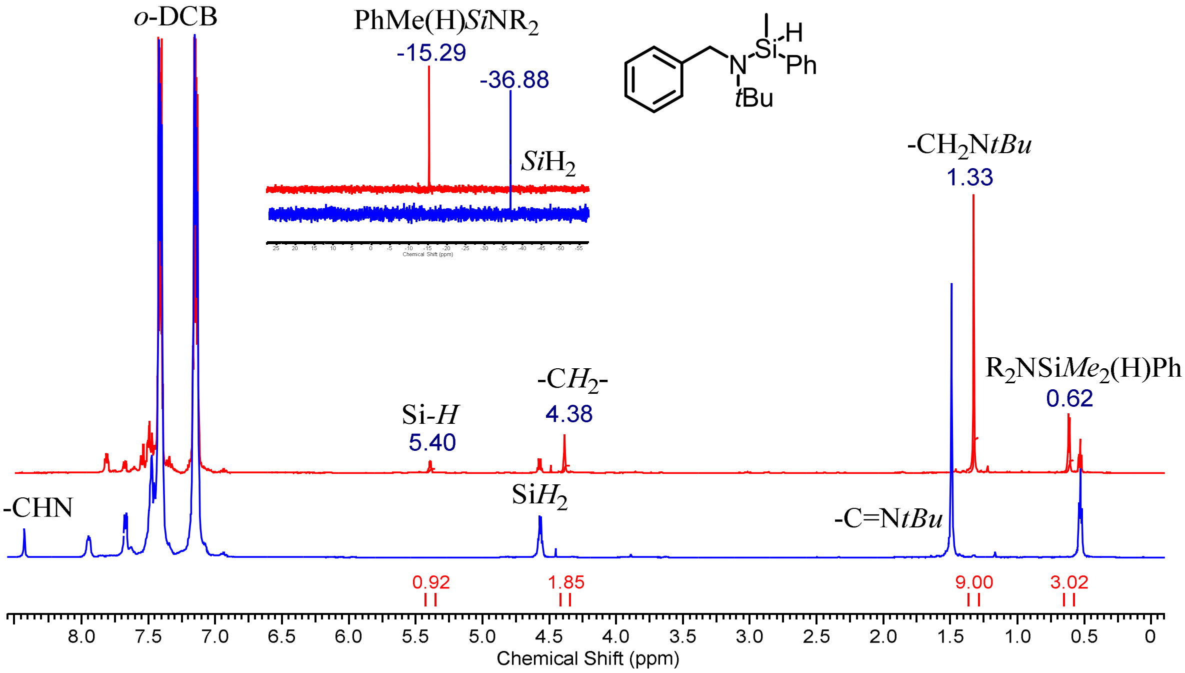

Figure S25. *In situ* ^{1}H and ^{29}Si{^{1}H}-NMR spectra of the hydrosilylation of *N*-benzylidene *t*-butylamine with $PhMeSiH_2$ in *o*-DCB, before the addition of mesitylene (with capillary inserted containing wet d_6-DMSO). t = 5 min at r.t. (blue) *vs* t = 17 hours at 100°C (red).

N-Dimethylphenylsilyl-dibenzylamine

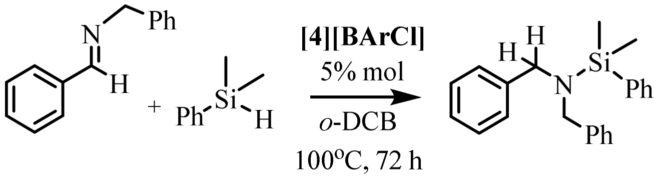

[4][BArCl] (11.5 mg, 0.013 mmol, 0.05 eq), *N*-benzylidene-benzylamine (50 µL, 0.263 mmol, 1.00 eq.) and dimethylphenylsilane (49 µL, 0.316 mmol, 1.20 eq.) in *o*-dichlorobenzene (0.5 mL). NMR conv. of imine = 43%. Diagnostic peaks: ^{1}H-NMR (400 MHz, *o*-DCB): δ 4.41 (s, 2H, -CH_2-). The assignment of the peaks was based on previous work.[5] The complex mixture of products is caused by the transimination of the intermediates. Traces of water from the reactants, consistent with siloxane formation, could have hydrolysed some products, increasing the number of products detected in the reaction mixture.

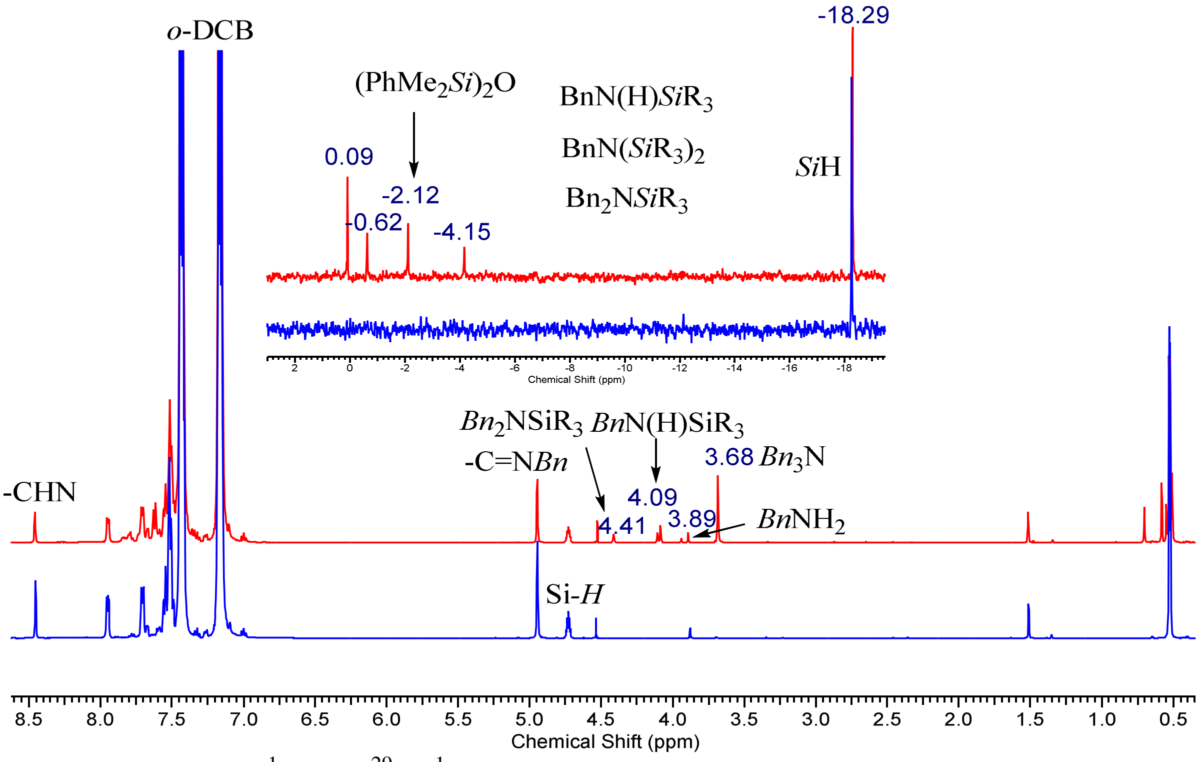

Figure S26. *In situ* ^{1}H and ^{29}Si{^{1}H}-NMR spectra of the hydrosilylation of *N*-benzylidene benzylamine with $PhMe_2SiH$ in *o*-DCB, before the addition of mesitylene (with capillary inserted containing wet d_6-DMSO). t = 5 min at r.t. (blue) *vs* t = 72 hours at 100°C (red).

N-Methyl-N-dimethylphenylsilyl-benzylamine

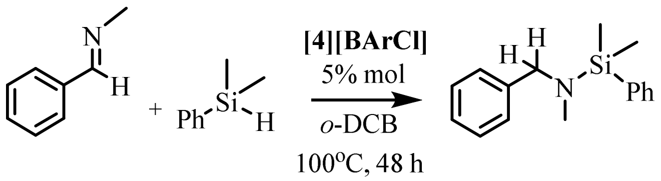

[4][BArCl] (8.6 mg, 0.010 mmol, 0.05 eq), *N*-benzylidene-methylamine (25 µL, 0.197 mmol, 1.00 eq.) and dimethylphenylsilane (37 µL, 0.236 mmol, 1.20 eq.) in *o*-dichlorobenzene (0.5 mL). NMR conv. of imine = 22%. Diagnostic peaks of Bn_2NMe (upon imine scrambling): ^{1}H-NMR (400 MHz, *o*-DCB): δ 3.65 (s, 4H, -CH_2-), 2.32 (s, 3H, Me) ppm. The assignment of the peaks was based on our previous work.[5] Also in this case the complex mixture of products was observed due to the transimination of the intermediates.

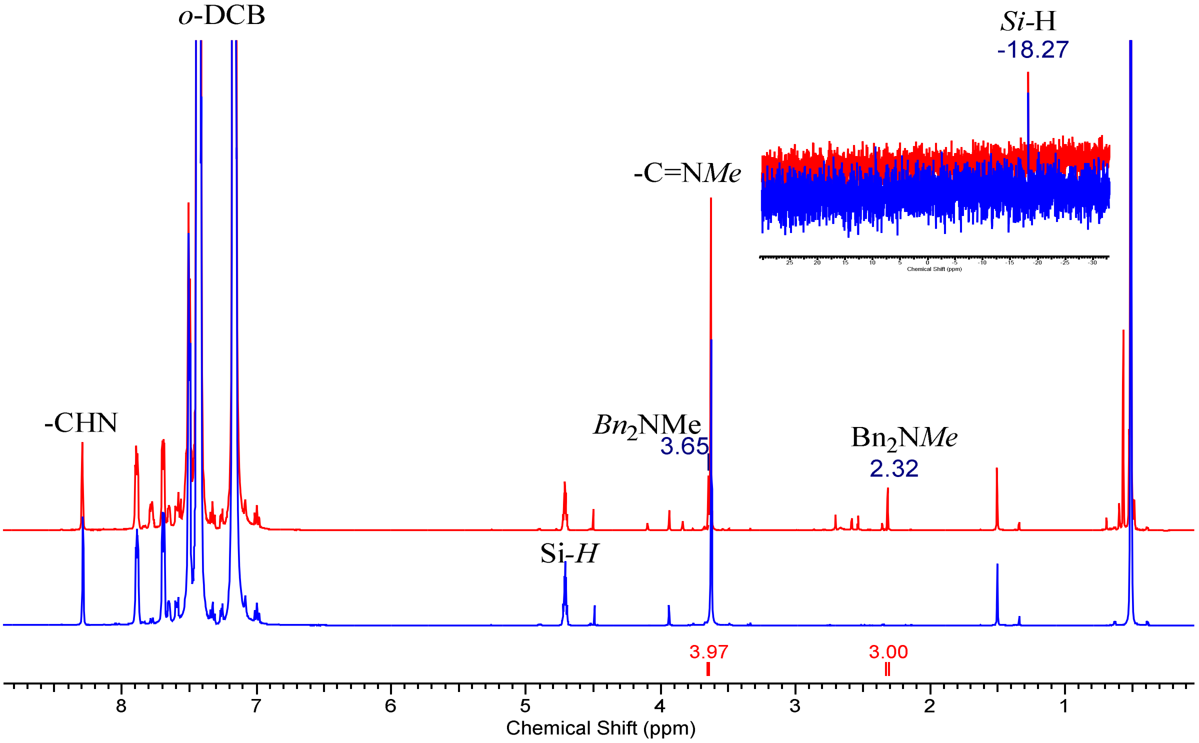

Figure S27. *In situ* ^{1}H and ^{29}Si{^{1}H}-NMR spectrum of the hydrosilylation of *N*-benzylidene methylamine with $PhMe_2SiH$ in *o*-DCB, before the addition of mesitylene (with capillary inserted containing wet d_6-DMSO). t = 5 min at r.t. (blue) *vs* t = 48 hours at 100°C (red).

N-Benzyl-N-dimethylphenylsilyl-aniline

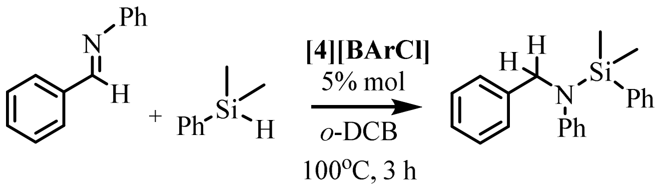

[4][BArCl] (10.7 mg, 0.012 mmol, 0.05 eq), N-benzylidene-aniline (45 mg, 0.246 mmol, 1.00 eq.) and dimethylphenylsilane (46 µL, 0.295 mmol, 1.20 eq.) in o-dichlorobenzene (0.5 mL). NMR yield = 60%. Diagnostic peaks: ^{1}H-NMR (400 MHz, o-DCB): δ 4.85 (s, 2H, -CH_2-), 0.69 (s, 6H, -SiMe_2) ppm. ^{29}Si{^{1}H}-NMR (81 MHz, o-DCB): δ -0.07 ppm. The data were in accordance with those reported in the literature.[5] The conversion was fast but also in this case the transimination of the intermediates led to the formation of Bn_2NPh and $PhN(SiMe_2Ph)_2$ in 19% of yield each.

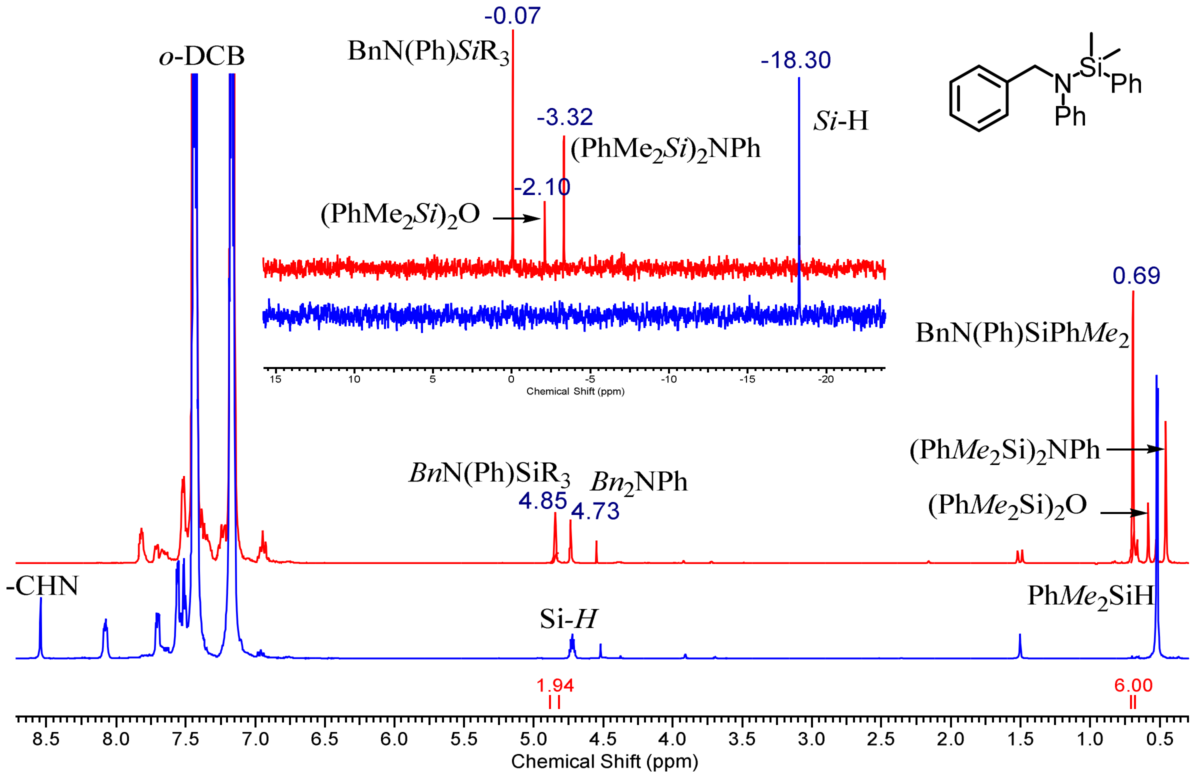

Figure S28. *In situ* ^{1}H and ^{29}Si{^{1}H}-NMR spectra of the hydrosilylation of N-benzylidene aniline with $PhMe_2SiH$ in o-DCB, before the addition of mesitylene (with capillary inserted containing wet d_6-DMSO). t = 5 min at r.t. (blue) *vs* t = 3 hours at 100°C (red).

***N*-Benzyl-*N*-methyldiphenylsilyl-aniline**

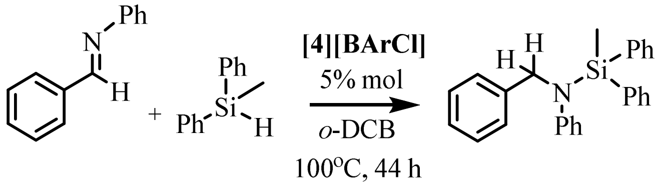

[4][BArCl] (10.7 mg, 0.012 mmol, 0.05 eq), *N*-benzylidene-aniline (45 mg, 0.246 mmol, 1.00 eq.) and methyldiphenylsilane (61 µL, 0.295 mmol, 1.20 eq.) in *o*-dichlorobenzene (0.5 mL). NMR yield = 73%. Diagnostic peaks: ^{1}H-NMR (400 MHz, *o*-DCB): δ 4.87 (s, 2H, -CH_2-), 0.97 (s, 3H, -Si*Me*) ppm. ^{29}Si{^{1}H}-NMR (81 MHz, *o*-DCB): δ -6.38 ppm. The hindrance of the silane made the reaction slower, but less transimination was observed (only 4% of Bn_2NPh).

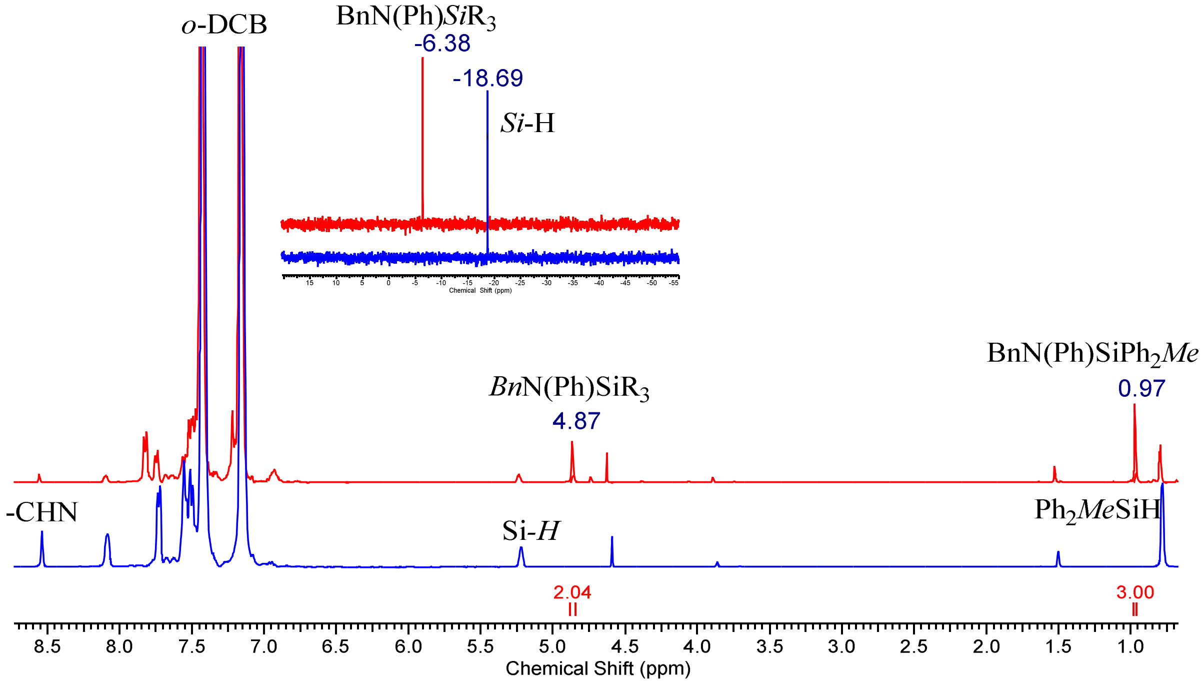

Figure S29. *In situ* ^{1}H and ^{29}Si{^{1}H}-NMR spectra of the hydrosilylation of *N*-benzylidene aniline with Ph_2MeSiH in *o*-DCB, before the addition of mesitylene (with capillary inserted containing wet *d*$_6$-DMSO). t = 5 min at r.t. (blue) *vs* t = 44 hours at 100°C (red).

11.3 Interaction study between [4]$^+$ and *N*-benzylidene-methylamine

A J. Youngs NMR tube equipped with a DMSO-d_6 capillary was loaded with **[4][BArCl]** (48.0 mg, 0.055 mmol, 1.00 eq) and *o*-dichlorobenzene (0.5 mL). To this solution, *N*-benzylidene-methylamine (7.0 µL, 0.055 mmol, 1.00 eq.) was added. After monitoring the initial reaction mixture by multinuclear ^{1}H-NMR spectroscopy, further equivalents of imine were added at r.t. to the reaction mixture which was analysed spectroscopically at each addition. The initial broadening of the methyl group of the imine disappeared upon the addition of 5 or more equivalents of the imine.

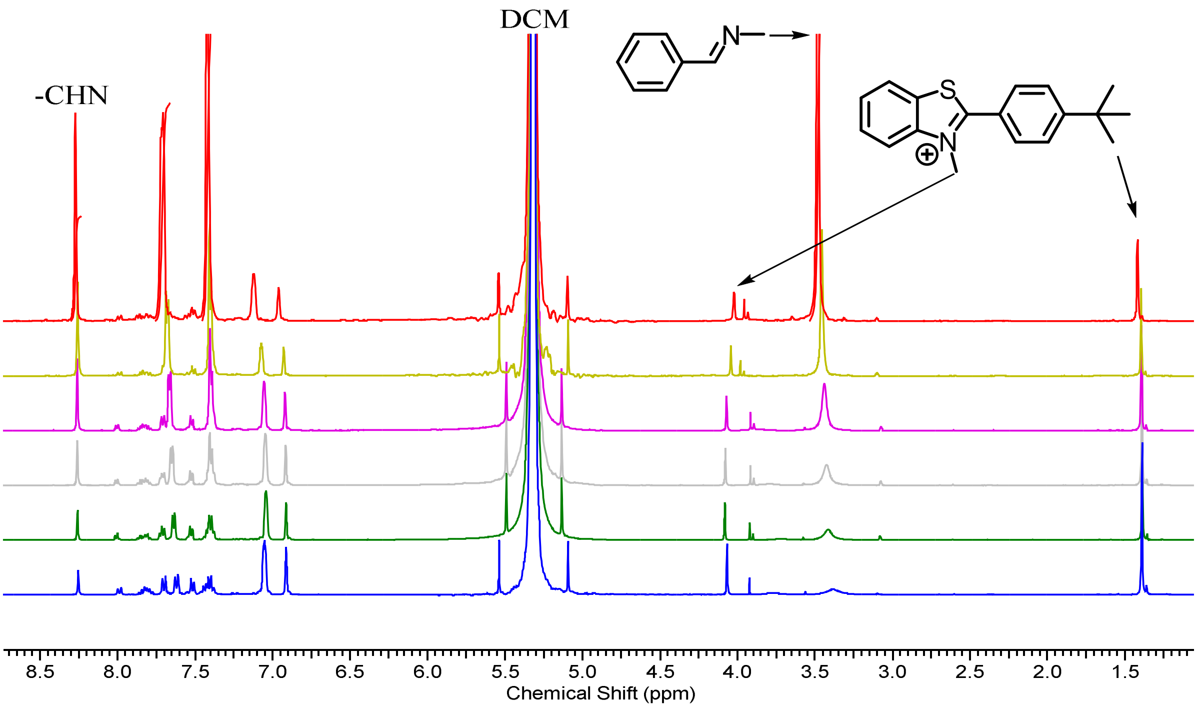

Figure S30. *In situ* ^{1}H-NMR spectra of the interaction study between [4][BArCl] and *N*-benzylidene-methylamine at r.t. (with capillary inserted containing wet d_6-DMSO). [4][BArCl] : imine = 1:1 (blue), 1:2 (green), 1:3 (grey), 1:5 (pink), 1:10 (yellow), 1:20 (red).

12. Attempted hydrosilylation of alkynes

Reaction using a terminal alkyne

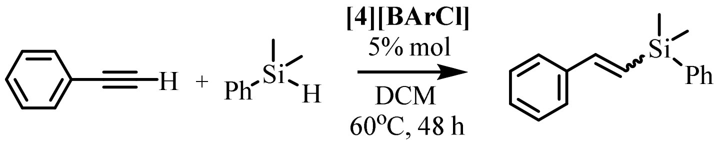

[4][BArCl] (9.8 mg, 0.011 mmol, 0.05 eq), phenylacetylene (25 µL, 0.223 mmol, 1.00 eq.) and dimethylphenylsilane (42 µL, 0.268 mmol, 1.20 eq.) in dichloromethane (0.5 mL). After stirring at 60°C for 48 hours, no significant reactivity was observed. Similar results were obtained using *o*-DCB as solvent and increasing the temperature up to 100°C for 24 hours.

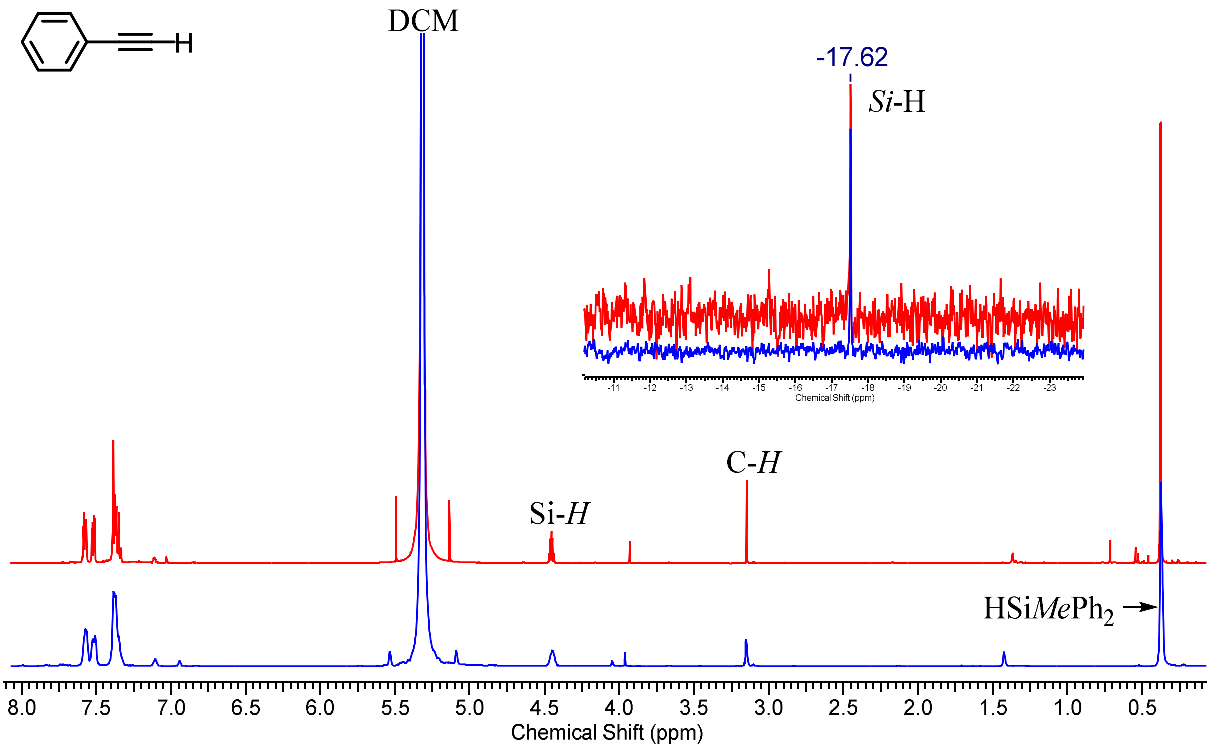

Figure S31. *In situ* ^{1}H and ^{29}Si{^{1}H}-NMR spectra of the attempted hydrosilylation of phenylacetylene with $PhMe_2SiH$ in DCM (with capillary inserted containing wet d_6-DMSO). t = 5 min at r.t. (blue) *vs* t = 48 hours at 60°C (red).

Reaction using an internal alkyne

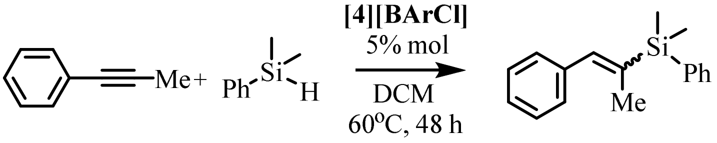

[4][BArCl] (10.4 mg, 0.012 mmol, 0.05 eq), 1-phenyl-1-propyne (30 μL, 0.237 mmol, 1.00 eq.) and dimethylphenylsilane (44 μL, 0.285 mmol, 1.20 eq.) in dichloromethane (0.5 mL). After stirring at 60ºC for 48 hours, no significant reactivity was observed. Similar results were obtained using *o*-DCB as solvent and increasing the temperature up to 100ºC for 24 hours.

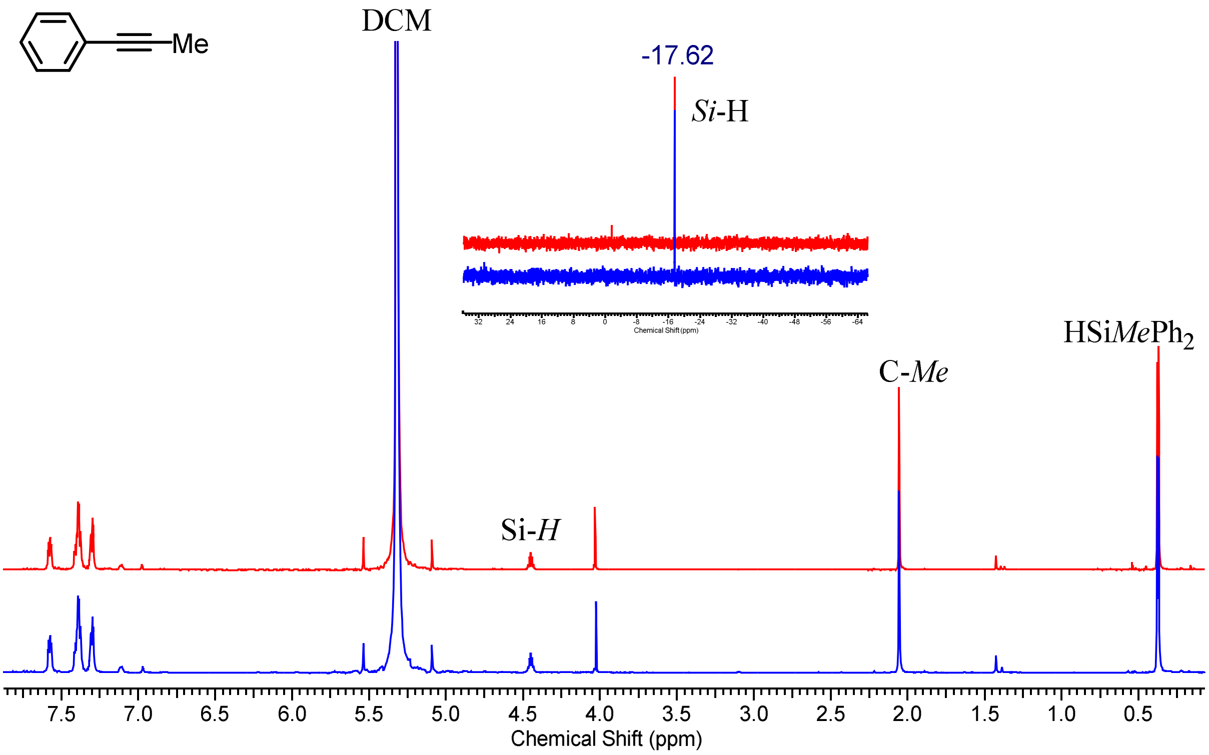

Figure S32. *In situ* ^{1}H and ^{29}Si{^{1}H}-NMR spectra of the attempted hydrosilylation of 1-phenyl-1-propyne with $PhMe_2SiH$ in DCM (with capillary inserted containing wet d_6-DMSO). t = 5 min at r.t. (blue) *vs* t = 48 hours at 60ºC (red).

13. Deoxygenation of phosphine oxide

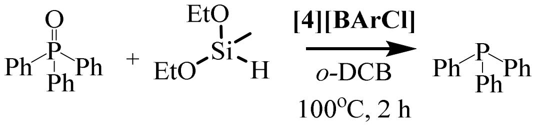

A J. Youngs NMR tube equipped with a DMSO-d_6 capillary was loaded with **[4][BArCl]** (6.5 mg, 0.007 mmol, 0.05 eq), triphenylphosphine oxide (42 mg, 0.148 mmol, 1.00 eq.) and *o*-dichlorobenzene (0.5 mL). To the obtained solution, $(EtO)_2MeSiH$ (86 µL, 0.518 mmol, 3.50 eq.) was added. After monitoring the initial reaction mixture by multinuclear NMR spectroscopy, the J. Youngs NMR tube was heated at 100°C for 2 hours. The reaction mixture was then analysed by ^{1}H, $^{29}Si\{^{1}H\}$ and $^{31}P\{^{1}H\}$-NMR spectroscopy. Subsequent addition of tris(2,4,6-trimethylphenyl)phosphine (11 mg) to the reaction mixture allowed the determination of the NMR yields based on the relative integral of the phopshorous of the product (NMR yield = >99%). Diagnostic peaks: $^{31}P\{^{1}H\}$-NMR (81 MHz, *o*-DCB): δ -6.23 ppm. The data were in accordance with those reported in the literature.[7]

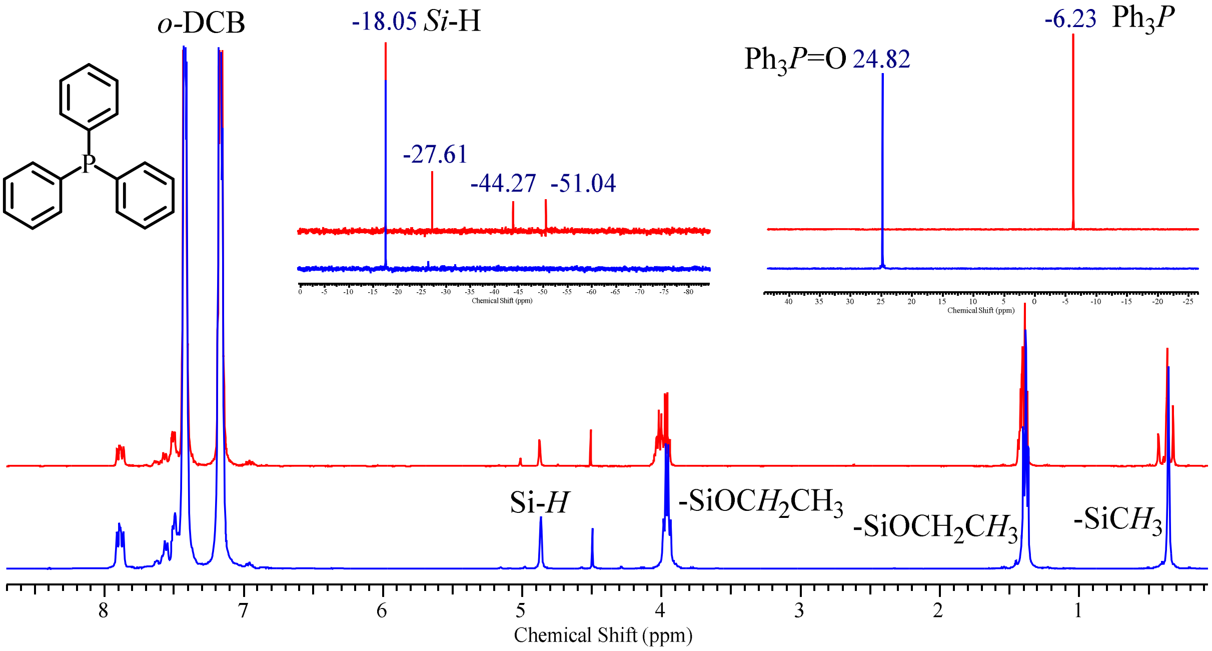

Figure S33. *In situ* ^{1}H, $^{29}Si\{^{1}H\}$ and $^{31}P\{^{1}H\}$-NMR spectra of the reduction of triphenylphosphine oxide with $(EtO)_2MeSiH$ using **[4][BArCl]** in *o*-DCB, before the addition of Mes_3P (with capillary inserted containing wet d_6-DMSO). t = 5 min at r.t. (blue) *vs* t = 2 hours at 100°C (red).

13.1 Control experiment using NaBArCl

As a control experiment, the deoxygenation of phosphine oxide with $(EtO)_2MeSiH$ (following the general procedure) was performed with NaBArCl (5 mol %) in *o*-DCB (2 hours at 100°C), but the product was detected only in traces.

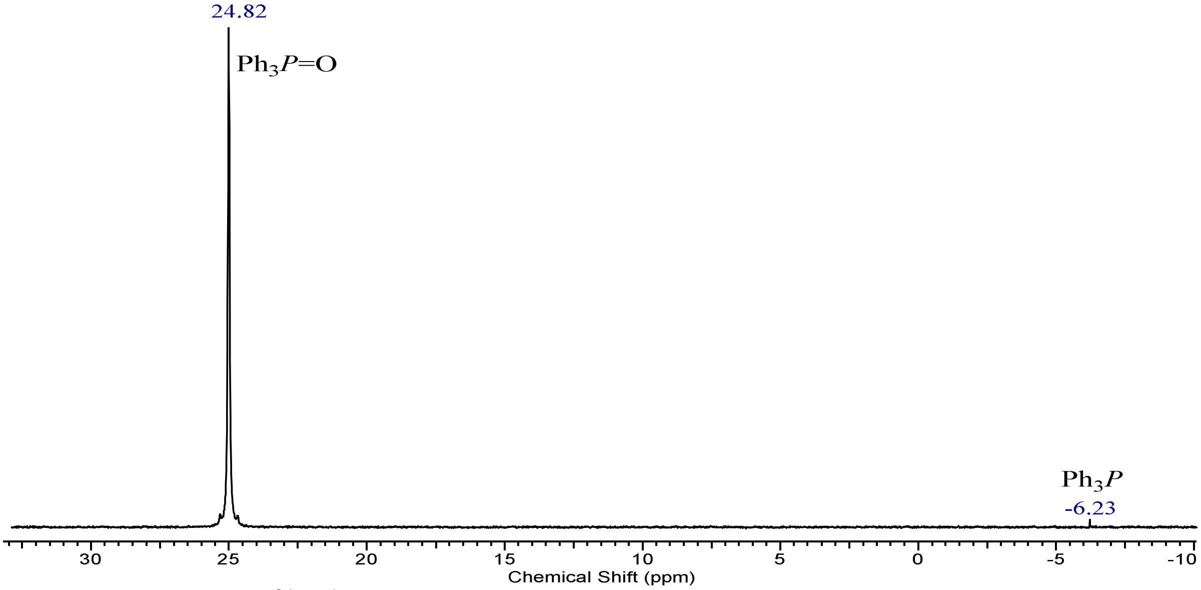

Figure S34. *In situ* $^{31}P\{^{1}H\}$-NMR spectrum of the reduction of triphenylphosphine oxide with $(EtO)_2MeSiH$ using NaBArCl in *o*-DCB, after 2 hours at 100°C (with capillary inserted containing wet d_6-DMSO).

14. Benzoxazolium and benzimidazolium catalysts

14.1 Synthesis of benzoxazolium and benzimidazolium salts

***N*-methyl-2-phenyl-benzoxazolium iodide ([7][I]):** Prepared according to general procedure B. 2-phenyl-benzoxazole (3.00 g, 15.40 mmol, 1.00 eq.) in excess of MeI (10.00 eq.) gave *N*-methyl-2-phenyl-benzoxazolium iodide as a yellow solid, after heating at 60°C for 1 week (1.04 g, 3.08 mmol, 20%). ^{1}H-NMR (400 MHz, $CDCl_3$): δ 8.37 (d, *J* = 8.2 Hz, 2H), 8.00-8.04 (m, 1H), 7.91-7.95 (m, 1H), 7.88 (t, *J* = 7.6 Hz, 1H), 7.75-7.84 (m, 4H), 4.58 (s, 3H) ppm. ^{13}C{^{1}H} NMR (100 MHz, $CDCl_3$): δ 163.7, 147.8, 135.9, 131.6, 130.1, 130.0, 128.9, 128.7, 119.5, 115.3, 113.2, 37.0 ppm. MS: m/z calcd for $C_{14}H_{12}NO^+$ (**[7]**$^+$) 210.1 Found ES$^+$ 210.4, m/z calcd for I^- (**[I]**$^-$)126.9 Found ES$^-$ 126.8; Accurate mass for $C_{14}H_{12}NO^+$ (**[7]**$^+$) 210.0913 Found 210.0910.

***N*-methyl-2-phenyl-benzoxazolium tetra(3,5-dichlorophenyl)borate ([7][BArCl]):** Prepared according to general procedure C. *N*-methyl-2-phenyl-benzoxazolium iodide (0.10 g, 0.30 mmol, 1.00 eq.) and NaBArCl (0.24 g, 0.33 mmol, 1.30 eq.) in DCM (2 mL) gave *N*-methyl-2-phenyl-benzoxazolium tetra(3,5-dichlorophenyl)borate as a white solid after recrystallization from hot DCM (0.22 g, 0.27 mmol, 89%). ^{1}H-NMR (400 MHz, ($CDCl_3$): δ 7.95 (t, *J* = 7.6 Hz, 1H), 7.81-7.86 (m, 4H), 7.71-7.77 (m, 3H), 7.51-7.60 (m, 1H), 7.02-7.07 (m, 8H), 6.85 (t, *J* = 2.0 Hz, 4H), 3.74 (s, 3H) ppm. ^{13}C{^{1}H} NMR (100 MHz, $CDCl_3$): δ 164.4 (q, $J_{B\text{-}C}$ = 48.9 Hz, BArCl), 163.1, 147.4, 137.3, 133.1 (BArCl, ArCl), 133.0 (q, $J_{B\text{-}C}$ = 3.9 Hz, BArCl, ArH), 131.7, 130.9, 130.8, 130.3, 129.6, 123.1 (BArCl, ArH), 118.0, 113.8, 113.2, 34.3 ppm. ^{11}B-NMR (128 MHz, $CDCl_3$): δ -6.93 ppm. MS: m/z calcd for $C_{14}H_{12}NO^+$ (**[7]**$^+$) 210.1 Found ES$^+$ 210.0, m/z calcd for $BC_{24}H_{12}Cl_8$ (**[BArCl]**$^-$) 594.8 Found ES$^-$ 595.0.

***N,N*-dimethyl-2-phenyl-benzimidazolium iodide ([8][I]):** Prepared according to the procedure reported in the literature.[8] To a solution of 2-phenyl-benzimidazole (1.00 g, 5.00 mmol, 1.00 eq.) in acetone (5 mL), iodomethane (0.94 mL, 15.00 mmol, 3.00 eq.) was added. The solution was stirred for 5 minutes and then a solution of NaOH (1.5 g) in water (1.5 mL) was added to it. The solution was then stirred overnight at room temperature, obtaining a brown solution with a precipitate. Then, to the reaction mixture, HCl conc. was added until neutralization, obtaining a yellow solution with a white precipitate. The solution was filtered and the solid was left under vacuum overnight, affording the *N,N*-dimethyl-2-phenyl-benzimidazolium iodide as an off-white solid (0.7 g). The spectroscopic data were in accordance with those reported in the literature.[8] ^{1}H-NMR (400 MHz, d_6-DMSO) δ 8.12-8.17 (m, 2H), 7.91 (d, *J* = 7.8 Hz, 2H), 7.82-7.87 (m, 1H), 7.80 (d, *J* = 7.3 Hz, 2H), 7.75-7.79 (m, 2H), 3.90 (s, 6H) ppm. ^{13}C{^{1}H}-NMR (100 MHz, d_6-DMSO) δ 150.3, 132.9, 131.7, 130.8, 129.5, 126.6, 121.0, 113.4, 32.8 ppm. MS: m/z calcd for $C_{15}H_{15}N_2^+$ (**[8]**$^+$) 223.3 Found ES$^+$ 223.4; Accurate mass for $C_{15}H_{15}N_2^+$ (**[8]**$^+$) 223.1230 Found 223.1219. *Due to the use of HCl in the work-up, some of the iodide of the product could have been exchanged with the chloride ion. For this reason, the yield and the ESI$^-$ of the product are not reported.*

***N,N*-dimethyl-2-phenyl-benzimidazolium tetra(3,5-dichlorophenyl)borate ([8][BArCl]):** Prepared according to the general procedure C**.** *N,N*-dimethyl-2-phenyl-benzimidazolium iodide (0.20 g, 0.57 mmol, 1.00 eq.) and NaBArCl (0.46 g, 0.74 mmol, 1.30 eq.) in DCM (2 mL) gave *N,N*-dimethyl-2-phenyl-benzimidazolium tetra(3,5-dichlorophenyl)borate as a white solid after recrystallization from hot DCM (0.37 g, 0.45 mmol, 79%).White solid (after recrystallization in hot DCM). ^{1}H-NMR (400 MHz, ($CDCl_3$): δ 7.84 (t, *J* = 7.6 Hz, 1H), 7.66-7.74 (m, 4H), 7.49-7.53 (m, 2H), 7.37 (d, *J* = 7.8 Hz, 2H), 6.97-7.01 (m, 8H), 6.81 (t, *J* = 2 Hz, 4H), 3.68 (s, 6H) ppm. ^{13}C{^{1}H}-NMR (100 MHz, $CDCl_3$) δ 164.2 (q, $J_{B\text{-}C}$ = 48.9 Hz, BArCl), 149.9, 134.2, 133.1 (BArCl, ArCl), 132.8 (q, $J_{B\text{-}C}$ = 3.9 Hz, BArCl, ArH), 131.3, 130.6, 129.6, 128.4, 123.0 (BArCl, ArH), 119.1, 112.6, 32.7 ppm. ^{11}B-NMR (128 MHz, $CDCl_3$): δ -6.98 ppm. MS: m/z calcd for $C_{15}H_{15}N_2^+$ (**[8]**$^+$) 223.3 Found ES$^+$ 223.0; m/z calcd for $BC_{24}H_{12}Cl_8$ (**[BArCl]**$^-$) 594.8 Found ES$^-$ 594.9.

14.2 Hydride transfer reactions

[2][BArCl] + *N,N*-dimethyl-2-phenyl-benzimidazoline

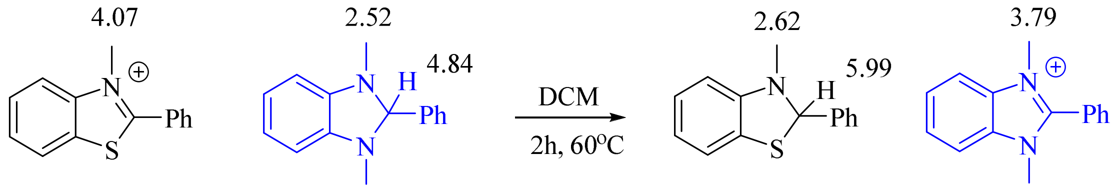

A J. Youngs NMR tube equipped with a DMSO-d_6 capillary was loaded with **[2][BArCl]** (16.2 mg, 0.020 mmol, 1.00 eq.), *N,N*-dimethyl-2-phenyl-benzimidazoline (4.5 mg, 0.020 mmol, 1.00 eq.) and dichloromethane (0.5 mL). After monitoring the initial reaction mixture by multinuclear NMR spectroscopy, the J. Youngs NMR tube was heated at 60°C for 2 hours. The reaction mixture was then analysed by ^{1}H-NMR spectroscopy. Diagnostic peaks: ^{1}H-NMR (400 MHz, DCM): δ 5.99 (s, 1H, C*H* benzothiazoline), 3.79 (s, 6H, N*Me* benzimidazolium), 2.62 (s, 3H, N*Me* benzothiazoline) ppm.

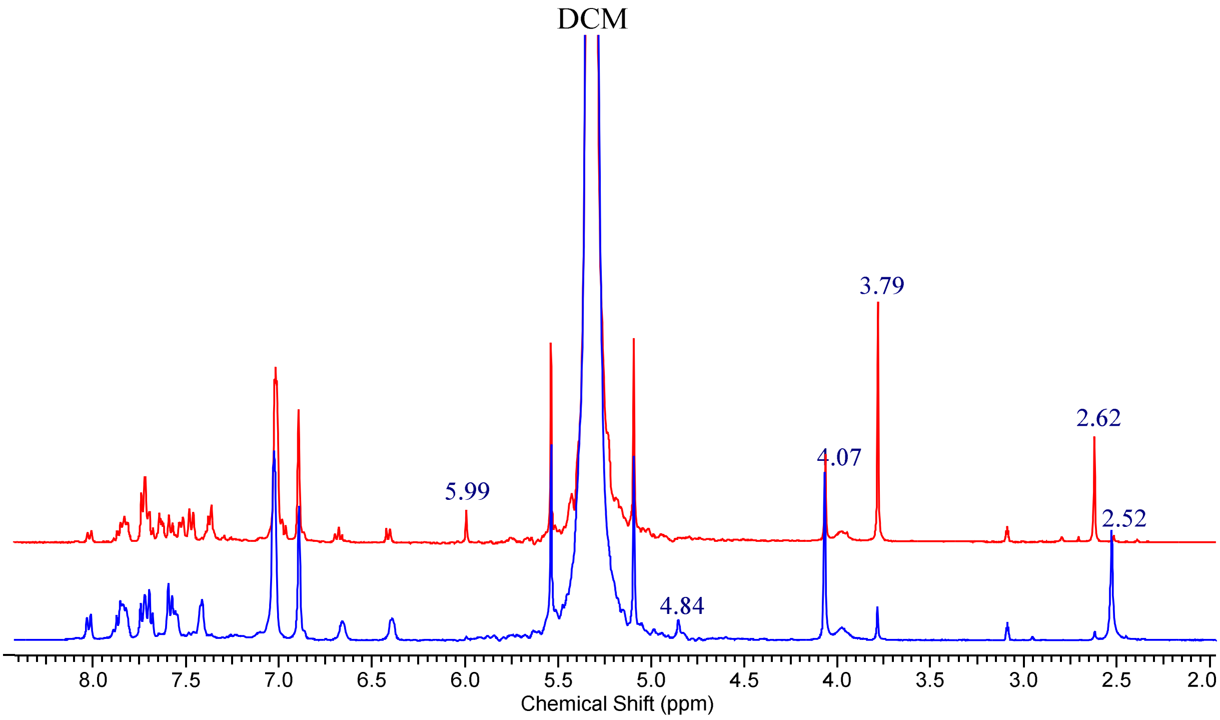

Figure S35. *In situ* ^{1}H-NMR spectra of the reaction between **[2][BArCl]** (1eq.) and benzimidazoline (1 eq.) in dichloromethane at t = 5 min at r.t. (blue) and after 2 hours at 60°C (red) (with capillary inserted containing wet d_6-DMSO).

[7][BArCl] + *N,N*-dimethyl-2-phenyl-benzimidazoline

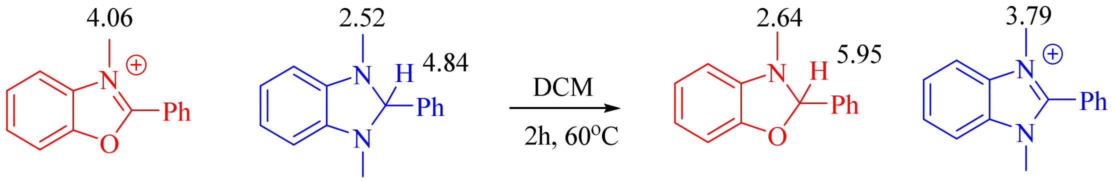

A J. Youngs NMR tube equipped with a DMSO-d_6 capillary was loaded with **[7][BArCl]** (16.1 mg, 0.020 mmol, 1.00 eq.), *N,N*-dimethyl-2-phenyl-benzimidazoline (4.5 mg, 0.020 mmol, 1.00 eq.) and dichloromethane (0.5 mL). After monitoring the initial reaction mixture by multinuclear NMR spectroscopy, the J. Youngs NMR tube was heated at 60°C for 2 hours. The reaction mixture was then analysed by ^{1}H-NMR spectroscopy. Diagnostic peaks: ^{1}H-NMR (400 MHz, DCM): δ 5.95 (s, 1H, C*H* benzoxazoline), 3.79 (s, 6H, N*Me* benzimidazolium), 2.64 (s, 3H, N*Me* benzoxazoline) ppm.

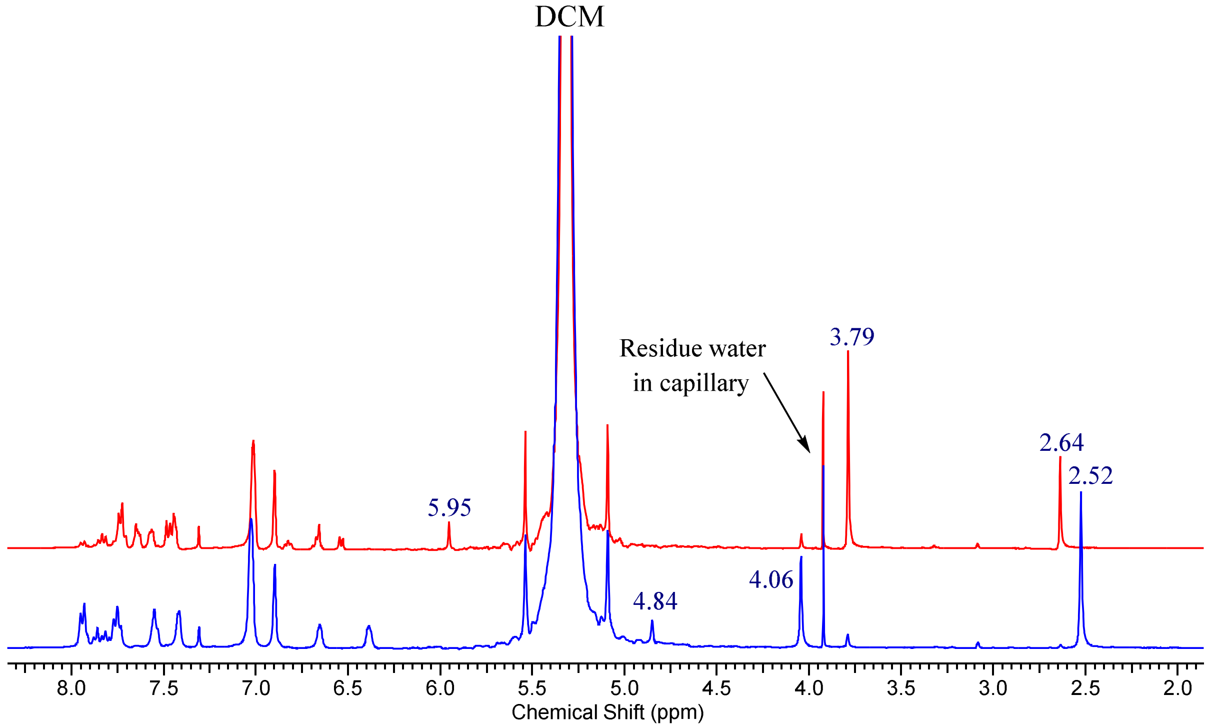

Figure S36. *In situ* ^{1}H-NMR spectra of the reaction between **[7][BArCl]** (1eq.) and benzimidazoline (1 eq.) in dichloromethane at t = 5 min at r.t. (blue) and after 2 hours at 60°C (red) (with capillary inserted containing wet d_6-DMSO).

14.3 Hydrosilylation of *N*-benzylidene-*t*-butylamine with [7][BArCl] and [8][BArCl]

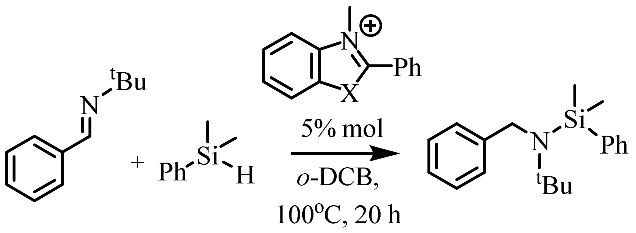

For the general procedure and the reaction spectra see section 11.

Entry 1**: [2][BArCl]** (9.8 mg, 0.012 mmol, 0.05 eq.), *N*-benzylidene-*t*-butylamine (43 µL, 0.238 mmol, 1.00 eq.) and dimethylphenylsilane (44 µL, 0.286 mmol, 1.20 eq.) in *o*-DCB.
Entry 2**: [7][BArCl]** (9.7 mg, 0.012 mmol, 0.05 eq.), *N*-benzylidene-*t*-butylamine (43 µL, 0.238 mmol, 1.00 eq.) and dimethylphenylsilane (44 µL, 0.286 mmol, 1.20 eq.) in *o*-DCB.
Entry 3**: [8][BArCl]** (9.8 mg, 0.012 mmol, 0.05 eq.), *N*-benzylidene-*t*-butylamine (43 µL, 0.238 mmol, 1.00 eq.) and dimethylphenylsilane (44 µL, 0.286 mmol, 1.20 eq.) in *o*-DCB.

**Entry**	**X (Catalyst)**	**Yield**[a]
1	S (**[2][BArCl]**)	24
2	O (**[7][BArCl]**)	25
3	NMe (**[8][BArCl]**)	38

[a] The yields were determined by ^{1}H-NMR spectroscopy using mesitylene as internal standard. The values reported are averages of two runs for each entry.

15. X-Ray Crystallography

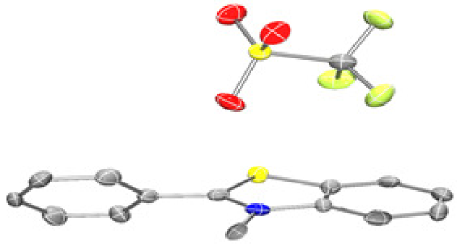

Single crystals of **[2][OTf]** were isolated from an acetonitrile solution layered with *o*-DCB. A suitable crystal was selected and mounted on an Agilent Supernova diffractometer, with Mo Kα radiation (mirror monochromator, λ = 0.7107 Å). The CrysAlisPro[9] software package was used for data collection, cell refinement and data reduction. The CrysAlisPro software package was also used for empirical absorption corrections, which were applied using spherical harmonics, implemented in SCALE3 ABSPACK scaling algorithm. The crystal was kept at 150 K during data collection. Using Olex2,[10] the structure was solved with the Superflip[11-13] structure solution program using Charge Flipping and refined with the XL[14] refinement package using Least Squares minimisation.

**Compound**	**[2][OTf]**
Empirical formula	$C_{15}H_{12}F_3NO_3S_2$
Formula weight	375.38
Temperature (K)	150(10)
Crystal system	triclinic
Space group	P-1
a (Å)	6.6141(5)
b (Å)	7.8819(8)
c (Å)	16.0543(17)
α (°)	84.388(9)
β (°)	81.549(8)
γ (°)	68.767(8)
Volume ($Å^3$)	770.75(13)
Z	2
ρ_{calc} (g/cm^3)	1.617
μ (mm^{-1})	0.393
F (000)	384.0
Crystal size (mm^3)	0.4 × 0.3 × 0.1
Radiation	Mo Kα (λ = 0.71073)
2Θ range for data collection/°	6.66 to 50.04
Reflections collected	7528
Independent reflections	2090 [R_{int} = 0.0892, R_{sigma} = 0.0860]
Data/restraints/parameters	2090/0/218
Goodness-of-fit on F^2	1.163
Final R indexes [I>=2σ (I)]	R_1 = 0.0808, wR_2 = 0.1502
Final R indexes [all data]	R_1 = 0.1097, wR_2 = 0.1596
Largest diff. peak/hole (e $Å^{-3}$)	0.37/-0.34

16. Carbon Lewis acid HIA calculation coordinates

Calculations were performed using the Gaussian09[15] suite of programmes. Structures were optimisated at the M06-2X/6-311G(d,p) level with PCM(Dichloromethane) solvation.[16] In all cases, structures were confirmed as minima by frequency analysis and the absence of imaginary frequencies. Structures and energies for Et_3B and Et_3BH were taken from prior work.[17] Full Cartesian coordinates for the optimised geometries for the HIA determinations are provided below.

_N_-methyl-2-phenyl-benzothiazolium [2]$^+$

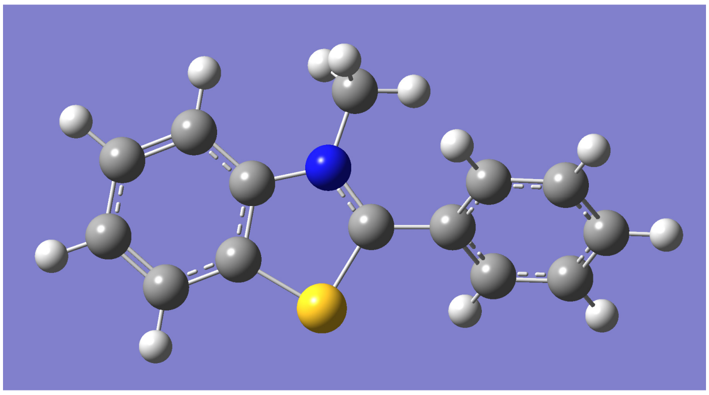

C	-1.792200	-1.177700	0.349300
C	-0.433400	-1.392200	0.494000
C	0.413800	-0.300100	0.315700
C	-0.090800	0.960700	-0.002300
C	-1.461400	1.177500	-0.146500
C	-2.298800	0.092500	0.034900
H	-2.477300	-2.004600	0.483700
H	-0.038200	-2.369300	0.738300
H	-1.856300	2.156900	-0.381500
H	-3.367900	0.228400	-0.065700
N	0.927600	1.908200	-0.149500
C	2.143000	1.431100	0.067200
C	0.618000	3.273600	-0.596500
H	-0.061600	3.201600	-1.443600
H	0.146100	3.822500	0.217700
H	1.533900	3.765600	-0.906800
C	3.389800	2.200300	0.033100
C	3.490700	3.405800	0.736400
C	4.495900	1.683100	-0.647800
C	4.694600	4.094400	0.742000
H	2.643900	3.784600	1.296800
C	5.692600	2.386000	-0.642900
H	4.411600	0.751300	-1.194500
C	5.792000	3.588300	0.050300
H	4.777600	5.023100	1.291800
H	6.546400	1.994100	-1.180700
H	6.728800	4.131600	0.056000

S	2.147400	-0.235700	0.454600

Total Energy: -993.3859025 Hartrees

_N_-methyl-2-phenyl-benzothiazoline 2-H

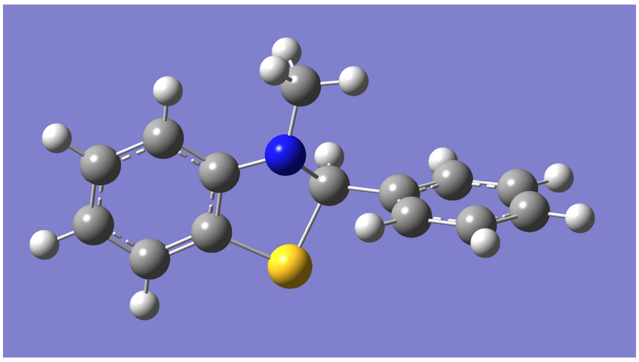

C	-1.876800	-0.950900	0.716100
C	-0.505000	-1.201700	0.822800
C	0.384800	-0.207300	0.466400
C	-0.057800	1.048100	0.019400
C	-1.422200	1.285100	-0.107100
C	-2.322900	0.278700	0.251900
H	-2.585700	-1.723300	0.986300
H	-0.143500	-2.162400	1.169700
H	-1.787600	2.235800	-0.474000
H	-3.385600	0.465400	0.154600
N	0.974600	1.954700	-0.226900
C	2.228800	1.261900	-0.507100
C	0.704200	3.092400	-1.090400
H	0.425600	2.780500	-2.106500
H	-0.102000	3.691700	-0.667700
H	1.595900	3.717100	-1.139300
C	3.448800	2.085800	-0.186000
C	3.503800	2.834900	0.990500
C	4.533400	2.093400	-1.058100
C	4.633300	3.585700	1.285600
H	2.651200	2.825800	1.660000
C	5.669800	2.841400	-0.758300
H	4.488800	1.515800	-1.975600
C	5.720200	3.588500	0.412500
H	4.668900	4.170300	2.197300
H	6.510600	2.842700	-1.441500
H	6.601300	4.174400	0.645900
S	2.154700	-0.256800	0.543900
H	2.265900	0.935700	-1.556500

Total energy: -994.1489075 Hartrees

_N_-methyl-2-(4-_t_-butylphenyl)-benzothiazolium [4]$^+$

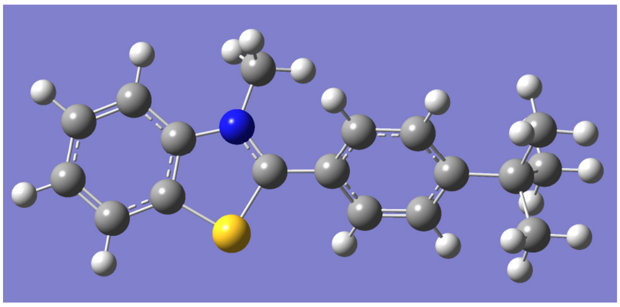

C	-1.823500	-1.156300	0.472200
C	-0.477900	-1.337700	0.736900
C	0.379000	-0.267700	0.485300
C	-0.102400	0.939400	-0.022400
C	-1.460400	1.123700	-0.284100
C	-2.308000	0.061200	-0.029400
H	-2.515100	-1.967700	0.658400
H	-0.100600	-2.273800	1.126900
H	-1.840000	2.063200	-0.663500
H	-3.367400	0.174000	-0.219800
N	0.924600	1.869100	-0.218900
C	2.122900	1.434600	0.142100
C	0.654200	3.161500	-0.865100
H	-0.015100	2.982000	-1.704000
H	0.188600	3.838200	-0.149700
H	1.587400	3.578900	-1.229700
C	3.364500	2.204900	0.097800
C	3.411900	3.507200	0.599500
C	4.535000	1.610800	-0.388100
C	4.609800	4.208300	0.596400
H	2.524700	3.964800	1.021500
C	5.717900	2.327500	-0.393800
H	4.511100	0.601900	-0.783200
C	5.784000	3.640000	0.095000
H	4.615800	5.211500	0.999900
H	6.607200	1.852500	-0.790000
S	2.098200	-0.170600	0.737800
C	7.116300	4.387100	0.070900
C	6.993200	5.805500	0.634500
H	6.671300	5.798900	1.678800
H	7.969000	6.293500	0.588800
H	6.290800	6.409500	0.054600
C	8.139700	3.609900	0.916800
H	8.311800	2.606500	0.522000
H	9.094800	4.140900	0.916400
H	7.797000	3.518200	1.950300
C	7.613400	4.481300	-1.382100
H	7.771600	3.494600	-1.821700
H	6.895000	5.023200	-2.002200

H	8.564600	5.018700	-1.409400

Total energy: -1150.607169 Hartrees

N-methyl-2-(4-*t*-butylphenyl)-benzothiazoline 4-H

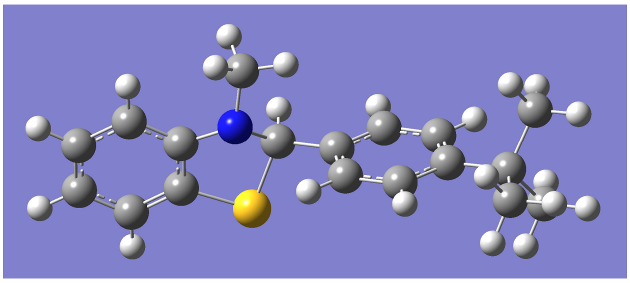

C	-1.982300	-0.648100	1.054900
C	-0.627600	-0.874000	1.320400
C	0.310200	-0.015200	0.781600
C	-0.068500	1.083800	-0.006800
C	-1.414600	1.290900	-0.287500
C	-2.363900	0.420600	0.255900
H	-2.727700	-1.318200	1.464700
H	-0.316700	-1.715000	1.928600
H	-1.728000	2.116100	-0.914200
H	-3.412500	0.583700	0.037800
N	1.001800	1.884300	-0.409500
C	2.250100	1.126600	-0.415900
C	0.814600	2.755100	-1.558400
H	0.569700	2.188400	-2.467300
H	0.013600	3.466100	-1.355700
H	1.731200	3.320400	-1.725200
C	3.475500	1.984800	-0.249500
C	3.489500	3.050400	0.645200
C	4.627200	1.708600	-0.982500
C	4.632100	3.827600	0.798900
H	2.594400	3.272900	1.215500
C	5.768400	2.482500	-0.818500
H	4.630600	0.883900	-1.688000
C	5.796900	3.560800	0.073000
H	4.602500	4.652600	1.498700
H	6.648900	2.241200	-1.403700
S	2.069500	-0.057600	0.991800
C	7.072300	4.394400	0.212700
C	6.915300	5.526400	1.232500
H	6.689200	5.140700	2.230000
H	7.850300	6.088100	1.294100
H	6.124500	6.222500	0.941700
C	8.225300	3.485200	0.670100
H	8.413700	2.684700	-0.048300
H	9.143400	4.070400	0.771900

H	7.997700	3.029700	1.637200
C	7.422300	5.015500	-1.150100
H	7.592300	4.249200	-1.909400
H	6.614200	5.665200	-1.496000
H	8.333100	5.614600	-1.064400
H	2.336000	0.530800	-1.336700

Total Energy: -1151.368591 Hartrees

N-methyl-2-(1-naphthyl)-benzothiazolium [5]$^+$

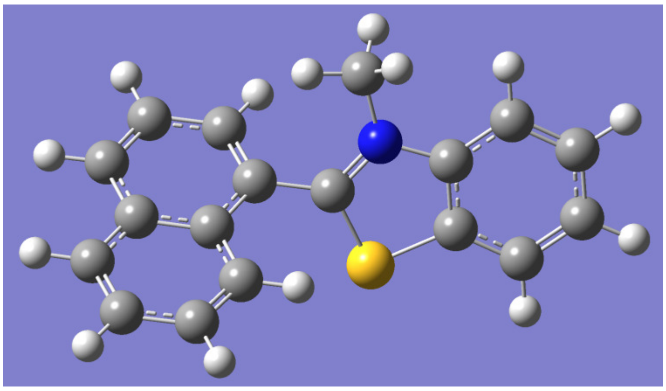

C	-1.872900	-1.038600	0.607200
C	-0.541100	-1.251800	0.913000
C	0.363700	-0.241300	0.590600
C	-0.061500	0.937300	-0.022500
C	-1.405500	1.153500	-0.329100
C	-2.299300	0.150300	-0.004900
H	-2.600400	-1.803600	0.846100
H	-0.210000	-2.165900	1.387300
H	-1.737000	2.071700	-0.795700
H	-3.349600	0.286700	-0.227100
N	1.004200	1.809300	-0.265600
C	2.177600	1.356000	0.140500
C	0.790200	3.092000	-0.950600
H	0.203200	2.900900	-1.846800
H	0.254900	3.767100	-0.283300
H	1.752400	3.514400	-1.221600
S	2.083100	-0.195400	0.855500
C	3.433200	2.121100	0.083800
C	4.538900	1.668500	-0.699700
C	3.518600	3.260900	0.851800
C	4.484000	0.534800	-1.552000
C	5.742000	2.425800	-0.639500
C	4.715800	4.002500	0.896100
H	2.667300	3.576900	1.444400
C	5.581300	0.162300	-2.282400
H	3.565900	-0.033400	-1.639300
C	6.860100	2.008800	-1.407100

C	5.801500	3.587200	0.172500
H	4.767600	4.891200	1.511400
C	6.784700	0.900700	-2.206100
H	5.526600	-0.704000	-2.930300
H	7.773200	2.590500	-1.351800
H	6.729200	4.147500	0.206200
H	7.642100	0.589900	-2.790100

Total Energy: -1147.00027777 Hartrees

N-methyl-2-(1-naphthyl)-benzothiazoline 5-H

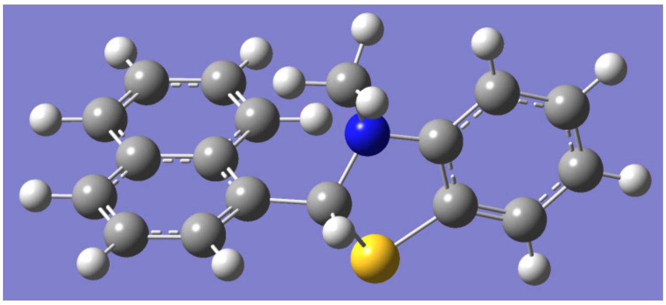

C	-1.931200	-0.980900	0.630100
C	-0.563600	-1.193400	0.832200
C	0.327000	-0.199000	0.477300
C	-0.112500	1.019700	-0.066000
C	-1.471900	1.217600	-0.285800
C	-2.372400	0.211100	0.072900
H	-2.639500	-1.754200	0.899400
H	-0.204300	-2.126100	1.250500
H	-1.833100	2.139000	-0.724600
H	-3.430800	0.368100	-0.097400
N	0.910200	1.938200	-0.294400
C	2.206500	1.278000	-0.429100
C	0.681400	3.036200	-1.215600
H	0.489100	2.682400	-2.238200
H	-0.167900	3.630400	-0.878300
H	1.560700	3.680400	-1.220600
S	2.090500	-0.217000	0.657100
H	2.335200	0.912300	-1.457600
C	3.391900	2.144700	-0.081100
C	3.487900	2.882200	1.145600
C	4.420100	2.201400	-0.990500
C	2.478100	2.879800	2.147700
C	4.662300	3.654200	1.379300
C	5.583500	2.967600	-0.751000
H	4.337700	1.644500	-1.917800
C	2.631400	3.603700	3.301900
H	1.577800	2.305300	1.991500
C	4.788400	4.391700	2.585400

C	5.699700	3.678500	0.409600
H	6.373400	2.986600	-1.491700
C	3.796900	4.369400	3.528300
H	1.849000	3.586600	4.051400
H	5.690800	4.972200	2.744100
H	6.584400	4.272700	0.611200
H	3.901100	4.934200	4.446900

Total Energy: -1147.76352357 Hartrees

N-methyl-2-(4-methoxyphenyl)-benzothiazolium [6]$^+$

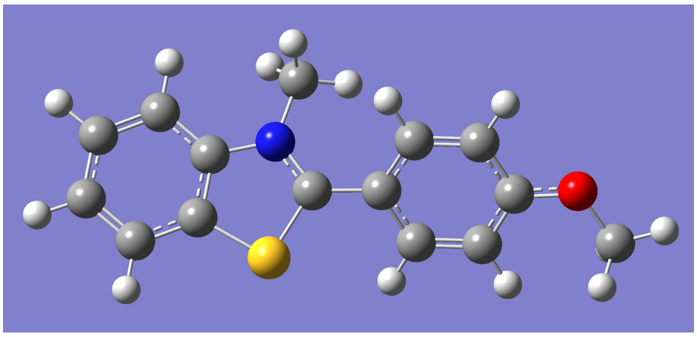

C	-1.851300	-1.182200	0.175400
C	-0.520200	-1.380500	0.497100
C	0.341100	-0.291800	0.380200
C	-0.120500	0.951800	-0.050900
C	-1.462600	1.152000	-0.373800
C	-2.315600	0.069900	-0.253300
H	-2.546300	-2.007600	0.259200
H	-0.156500	-2.343000	0.831800
H	-1.827400	2.117600	-0.697900
H	-3.363500	0.195100	-0.493100
N	0.908300	1.898500	-0.112600
C	2.090500	1.440400	0.276500
C	0.662200	3.234000	-0.673200
H	0.045900	3.113800	-1.561900
H	0.145500	3.851500	0.060800
H	1.609900	3.684800	-0.950100
C	3.323700	2.209900	0.366600
C	3.333200	3.489000	0.949400
C	4.528100	1.641100	-0.054700
C	4.516600	4.178100	1.084800
H	2.416600	3.925500	1.327400
C	5.721700	2.334700	0.067200
H	4.532900	0.656700	-0.508100
C	5.721300	3.611000	0.639700
H	4.547000	5.157900	1.543400
H	6.635700	1.878300	-0.284900
S	2.042900	-0.210800	0.737100

O	6.816300	4.363300	0.809000
C	8.072600	3.830100	0.401200
H	8.077600	3.628100	-0.672400
H	8.807100	4.596000	0.632400
H	8.303700	2.917500	0.955600

Total energy: -1107.901041 Hartrees

_N_-methyl-2-(4-methoxyphenyl)-benzothiazoline 6-H

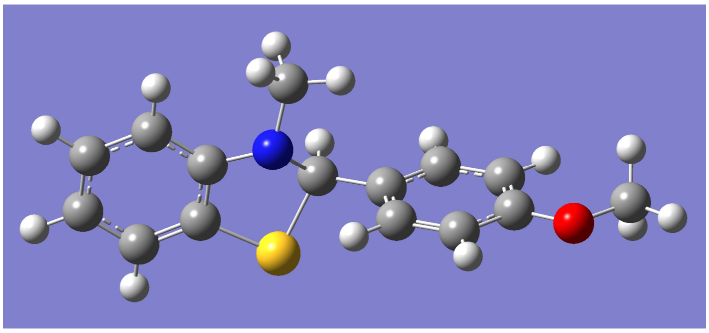

C	-2.072800	-0.832400	0.575100
C	-0.736200	-1.120500	0.870700
C	0.232000	-0.184400	0.564300
C	-0.099100	1.049600	-0.018400
C	-1.426200	1.321900	-0.332500
C	-2.406100	0.374800	-0.023100
H	-2.841700	-1.560000	0.802600
H	-0.460900	-2.065800	1.322800
H	-1.701700	2.255000	-0.807200
H	-3.440000	0.588600	-0.266700
N	0.992900	1.901500	-0.193800
C	2.242100	1.145700	-0.272400
C	0.876200	2.982500	-1.157800
H	0.705900	2.607700	-2.176700
H	0.051700	3.637900	-0.877400
H	1.793900	3.570000	-1.141200
C	3.449200	1.949200	0.124500
C	3.438400	2.725600	1.289500
C	4.596700	1.927100	-0.654500
C	4.549700	3.457400	1.655600
H	2.542300	2.749700	1.899600
C	5.731000	2.656900	-0.295900
H	4.616200	1.334600	-1.563400
C	5.707600	3.425500	0.865500
H	4.555900	4.065200	2.552200
H	6.608700	2.618700	-0.926300
S	1.975200	-0.285500	0.866400
O	6.744800	4.173800	1.311100
C	7.937300	4.172200	0.543100
H	7.758400	4.568500	-0.460500

H	8.634100	4.818700	1.070200
H	8.356800	3.165000	0.470500
H	2.383900	0.737600	-1.283900

Total energy: -1108.6613 Hartrees

N-methyl-2-phenyl-benzoxazolium [7]$^+$

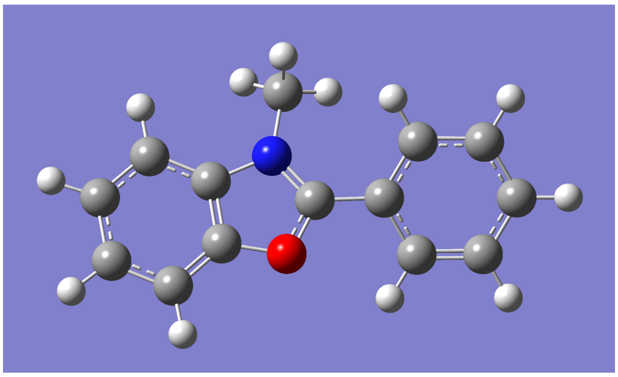

C	-1.448600	-1.279000	0.243300
C	-0.062100	-1.272200	0.329100
C	0.525700	-0.030700	0.191100
C	-0.190500	1.134500	-0.018100
C	-1.576300	1.134800	-0.104700
C	-2.186900	-0.104200	0.031100
H	-1.975000	-2.219100	0.344600
H	0.520900	-2.167500	0.493900
H	-2.146000	2.040800	-0.262200
H	-3.265800	-0.165800	-0.025600
N	0.771900	2.149600	-0.114400
C	1.962200	1.600900	0.053500
C	0.457200	3.542600	-0.440800
H	-0.386200	3.536100	-1.127700
H	0.194900	4.082300	0.467900
H	1.315900	3.998100	-0.926500
C	3.277300	2.216100	0.047900
C	3.476000	3.508700	0.545400
C	4.354500	1.460300	-0.430200
C	4.753500	4.048200	0.540900
H	2.655400	4.073200	0.968500
C	5.623800	2.015600	-0.437000
H	4.188800	0.456900	-0.801400
C	5.822200	3.307500	0.044100
H	4.915200	5.043200	0.934700
H	6.459200	1.440900	-0.815400
H	6.816900	3.735700	0.040000
O	1.861600	0.294000	0.231500

Total Energy: -670.412227 Hartrees

_N_-methyl-2-phenyl-benzoxazoline 7-H

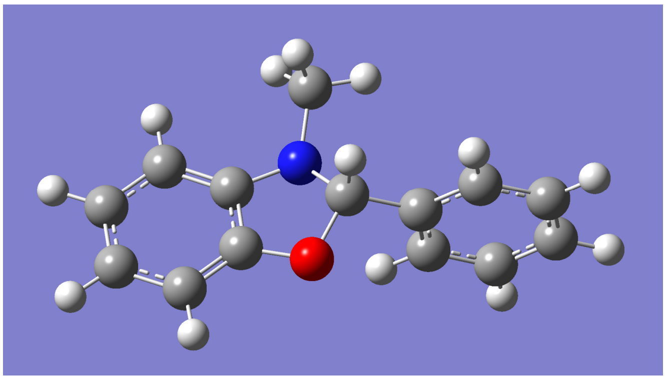

C	-1.567300	-0.927400	-0.829200
C	-0.173900	-1.048000	-0.689800
C	0.512400	0.070700	-0.286500
C	-0.120100	1.292000	-0.035000
C	-1.491500	1.410000	-0.157000
C	-2.208900	0.273000	-0.562800
H	-2.144400	-1.790600	-1.136900
H	0.342700	-1.979900	-0.879900
H	-1.998300	2.345000	0.047200
H	-3.285400	0.338500	-0.663700
N	0.875900	2.221100	0.278000
C	2.046600	1.416000	0.638100
C	0.576700	3.330700	1.161700
H	-0.237600	3.921000	0.742300
H	0.293200	2.990100	2.167500
H	1.456400	3.971700	1.235600
C	3.350300	2.066000	0.266400
C	3.553800	2.502000	-1.043000
C	4.351000	2.233700	1.216800
C	4.755300	3.099800	-1.396000
H	2.762200	2.372200	-1.772400
C	5.556100	2.838000	0.864300
H	4.188500	1.892700	2.233900
C	5.758100	3.269100	-0.441100
H	4.913500	3.438000	-2.413000
H	6.332600	2.970100	1.608100
H	6.694000	3.739700	-0.717800
O	1.863700	0.194200	-0.090700
H	2.031100	1.178200	1.717000

Total energy: -671.172217 Hartrees

N,N-dimethyl-2-phenyl-benzimidazolium [8]$^+$

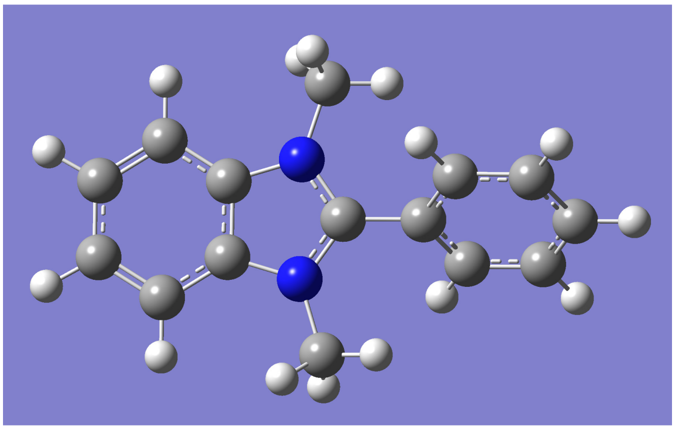

C	-1.567900	-1.283700	0.352200
C	-0.197400	-1.351800	0.534500
C	0.512200	-0.170900	0.330400
C	-0.122500	1.015700	-0.035600
C	-1.501200	1.085800	-0.220200
C	-2.207900	-0.087300	-0.018800
H	-2.163600	-2.175700	0.497300
H	0.295800	-2.273900	0.813100
H	-1.993900	2.007200	-0.502400
H	-3.282200	-0.085000	-0.150100
N	0.872600	1.976100	-0.160500
C	2.054500	1.409600	0.120700
C	0.631400	3.350400	-0.594500
H	-0.033700	3.326300	-1.456100
H	0.172300	3.915900	0.215900
H	1.576000	3.804800	-0.880400
C	3.354900	2.089500	0.088000
C	3.560200	3.240800	0.851600
C	4.378800	1.574700	-0.710800
C	4.795700	3.873200	0.815300
H	2.765300	3.626300	1.479300
C	5.609000	2.217000	-0.743300
H	4.206800	0.689000	-1.312100
C	5.816700	3.362900	0.018900
H	4.961900	4.762000	1.410500
H	6.402800	1.825000	-1.366200
H	6.778200	3.860600	-0.007900
N	1.867200	0.117200	0.424100
C	2.882600	-0.844900	0.848200
H	2.487700	-1.407200	1.692500
H	3.117600	-1.520900	0.026800
H	3.775100	-0.307600	1.157300

Total energy: -689.8732624 Hartrees

N,N-dimethyl-2-phenyl-benzimidazoline 8-H

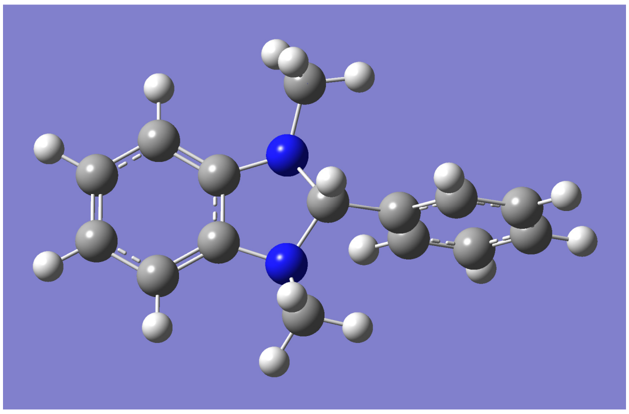

C	-1.639400	-0.963500	0.883200
C	-0.241900	-1.090900	0.963800
C	0.531300	-0.024500	0.550500
C	-0.060800	1.149700	0.049200
C	-1.432800	1.271300	-0.044500
C	-2.222300	0.192400	0.389600
H	-2.264300	-1.789800	1.199600
H	0.210000	-2.003000	1.334500
H	-1.891000	2.166800	-0.446400
H	-3.301000	0.266200	0.322700
N	0.960800	2.050800	-0.270900
C	2.179200	1.246300	-0.394700
C	0.740500	3.033300	-1.312700
H	0.553900	2.562800	-2.288900
H	-0.112600	3.659500	-1.051900
H	1.620000	3.674400	-1.388200
C	3.432200	2.019900	-0.062100
C	3.556900	2.626300	1.188400
C	4.462000	2.135600	-0.989300
C	4.703500	3.341700	1.504100
H	2.746300	2.527700	1.901400
C	5.614900	2.853500	-0.674100
H	4.361600	1.664100	-1.961900
C	5.735100	3.456700	0.571800
H	4.798500	3.811700	2.476000
H	6.413300	2.941600	-1.401200
H	6.629100	4.016500	0.820100
N	1.919800	0.148100	0.540500
C	2.776600	-1.013000	0.409300
H	2.590900	-1.702000	1.233200
H	2.611000	-1.539800	-0.541800
H	3.818600	-0.694300	0.463100
H	2.265100	0.846700	-1.430200

Total energy: -690.607451 Hartrees

17. References

[1] S. Ranjit and X. Liu, *Chem. Eur. J.,* **2011**, *17*, 1105 – 1108.

[2] R. Anulewicz-Ostrowska, T. Kliś, D. Krajewski, Ba. Lewandowski and J. Serwatowski, *Tetrahedron Letters*, **2003**, *44*, 7329–7331.

[3] T. J. Herrington, B. J. Ward, L. R. Doyle, J. McDermott, A. J. P. White, P. A. Hunt and A. E. Ashley, *Chem. Commun.*, **2014**, *50*, 12753-12756.

[4] B. Xu, R. A. A. Yanez, H. Nakatsuka, M. Kitamura, R. Fröhlich, G. Kehr, G. Erker, *Chem. Asian J.*, **2012**, *7*, 1347 – 1356.

[5] E. R. Clark and M. J. Ingleson, *Angew. Chem. Int. Ed.*, **2014**, *53*, 11306 –11309.

[6] D. V. Gutsulyak, S. F. Vyboishchikov and G. I. Nikonov, *J. Am. Chem. Soc.*, **2010**, *132*, 5950–5951.

[7] Y. Li, L. Lu, S. Das, S. Pisiewicz, K. Junge, and M. Beller, *J. Am. Chem. Soc.* **2012**, *134*, 18325−18329.

[8] I. Popov, *Chemistry of Heterocyclic Compounds*, **1996**, *32*, 672-681.

[9] CrysAlisPro, Agil. Technol. Version 1.1 71.35.19 (release 27-10-2011 CrysAlis171 .NET) (compiled Oct 27 2011,150211).

[10] O. V. Dolomanov, L. J. Bourhis, R. J. Gildea, J. A. K. Howard and H. Puschmann, *J. Appl. Crystallogr.*, **2009**, *42*, 339–341.

[11] L. Palatinus and G. Chapuis, *J. Appl. Crystallogr.*, **2007**, ***40***, 786–790.

[12] L. Palatinus and A. van der Lee, *J. Appl. Crystallogr.*, **2008**, *41*, 975–984.

[13] L. Palatinus, S. J. Prathapa and S. van Smaalen, *J. Appl. Crystallogr.*, **2012**, *45*, 575–580.

[14] G. M. Sheldrick, *Acta Crystallogr. Sect. A Found. Crystallogr.*, **2008**, *64*, 112–122.

[15] Gaussian 09, Revision C1, Frisch, M. J.; Trucks, G. W.; Schlegel, H. B.; Scuseria, G. E.; Robb, M. A.; Cheeseman, J. R.; Scalmani, G.; Barone, V.; Mennucci, B.; Petersson, G. A.; Nakatsuji, H.; Caricato, M.; Li, X.; Hratchian, H. P.; Izmaylov, A. F.; Bloino, J.; Zheng, G.; Sonnenberg, J. L.; Hada, M.; Ehara, M.; Toyota, K.; Fukuda, R.; Hasegawa, J.; Ishida, M.; Nakajima, T.; Honda, Y.; Kitao, O.; Nakai, H.; Vreven, T.; Montgomery, Jr., J. A.; Peralta, J. E.; Ogliaro, F.; Bearpark, M.; Heyd, J. J.; Brothers, E.; Kudin, K. N.; Staroverov, V. N.; Kobayashi, R.; Normand, J.; Raghavachari, K.; Rendell, A.; Burant, J. C.; Iyengar, S. S.; Tomasi, J.; Cossi, M.; Rega, N.; Millam, J. M.; Klene, M.; Knox, J. E.; Cross, J. B.; Bakken, V.; Adamo, C.; Jaramillo, J.; Gomperts, R.; Stratmann, R. E.; Yazyev, O.; Austin, A. J.; Cammi, R.; Pomelli, C.; Ochterski, J. W.; Martin, R. L.; Morokuma, K.; Zakrzewski, V. G.; Voth, G. A.; Salvador, P.; Dannenberg, J. J.; Dapprich, S.; Daniels, A. D.; Farkas, Ö.; Foresman, J. B.; Ortiz, J. V.; Cioslowski, J.; Fox, D. J. Gaussian, Inc., Wallingford CT, **2009**.

[16] http://comp.chem.umn.edu/info/DFT.htm.

[17] E. R. Clark, A. Del Grosso and M. J. Ingleson, *Chem. Eur. J.*, **2013**, *19*, 2462 – 2466.

18. NMR Spectra of all salts

_N_-methyl-2-phenyl-benzothiazolium iodide ([2][I])
^{1}H and ^{13}C{^{1}H}-NMR spectra

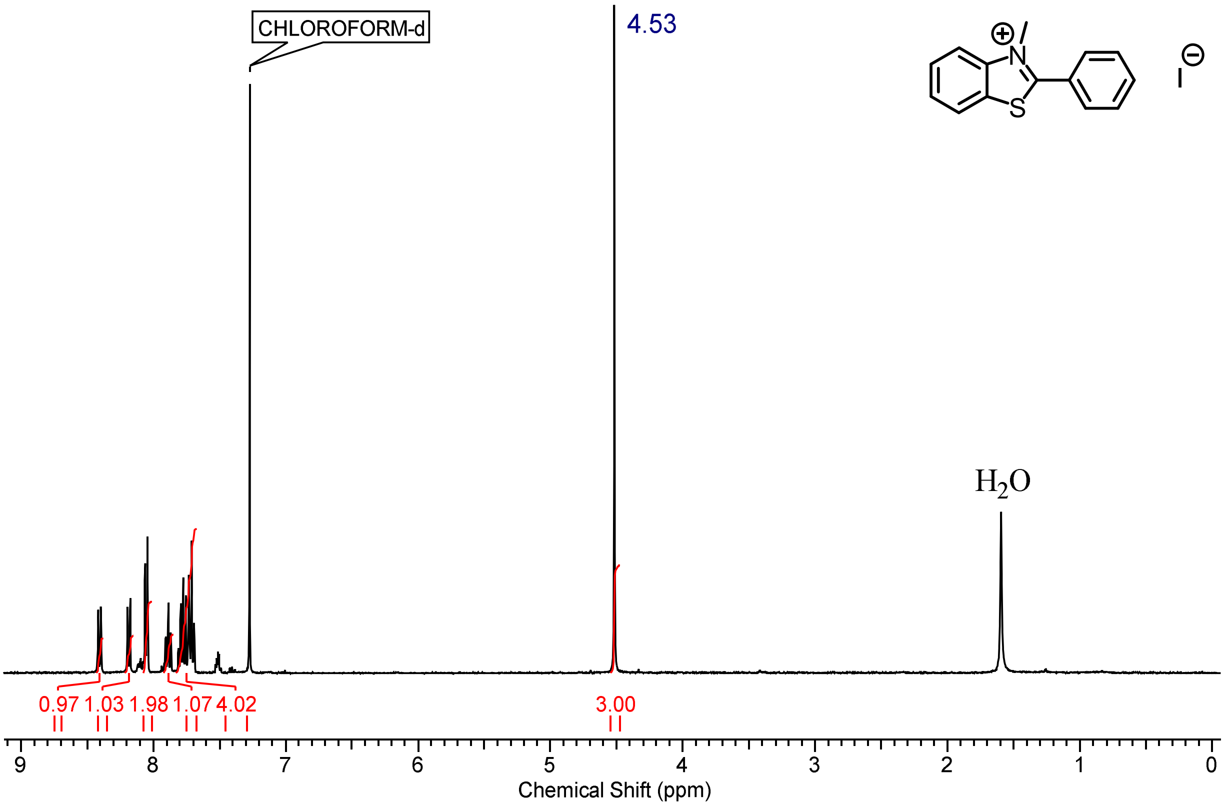

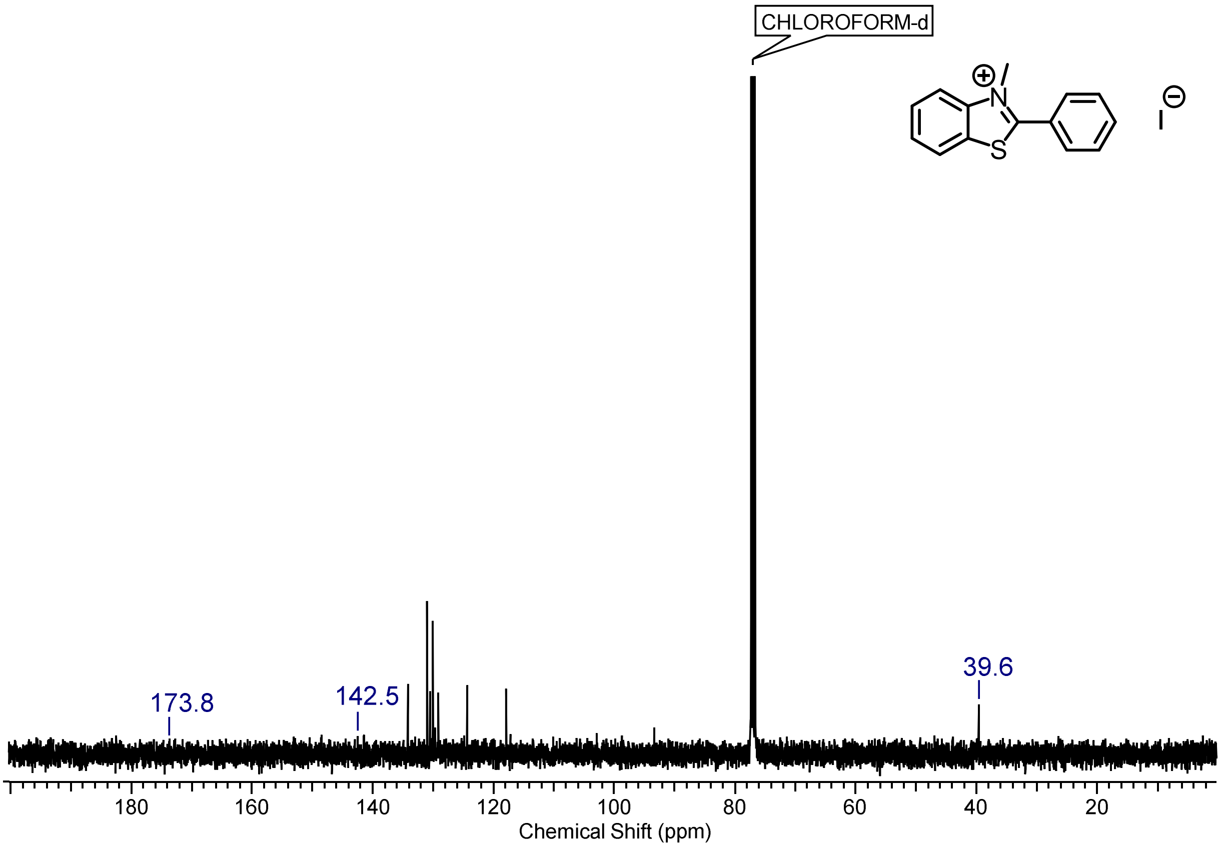

_N_-methyl-2-phenyl-benzothiazolium triflate ([2][OTf])
^{1}H, $^{13}C\{^{1}H\}$ and ^{19}F -NMR spectra

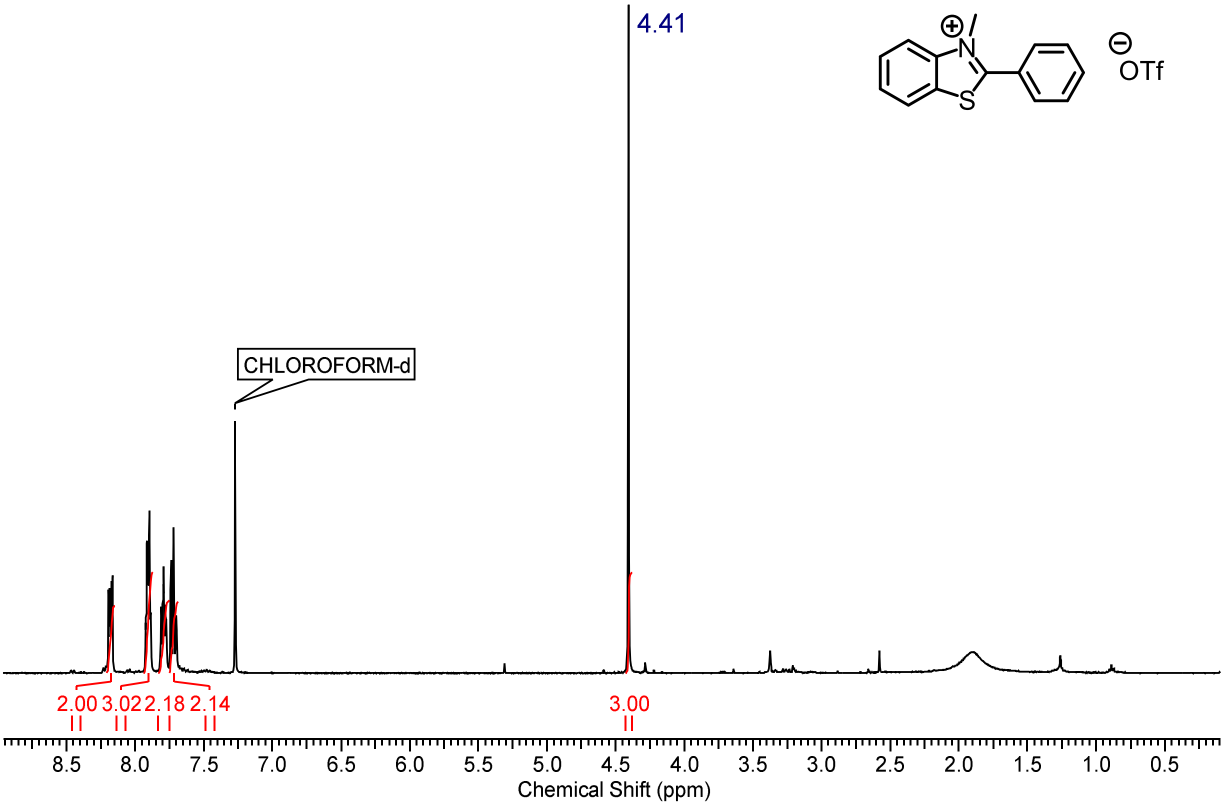

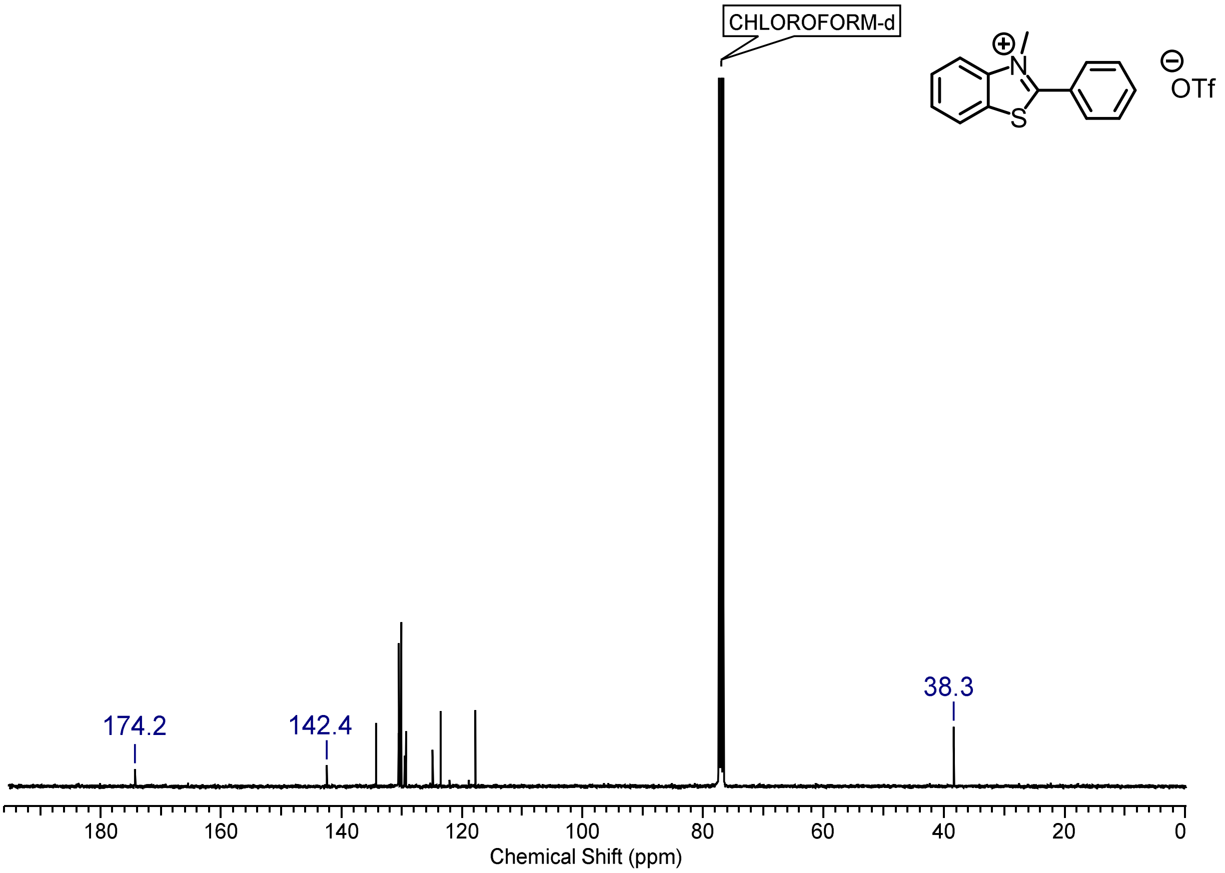

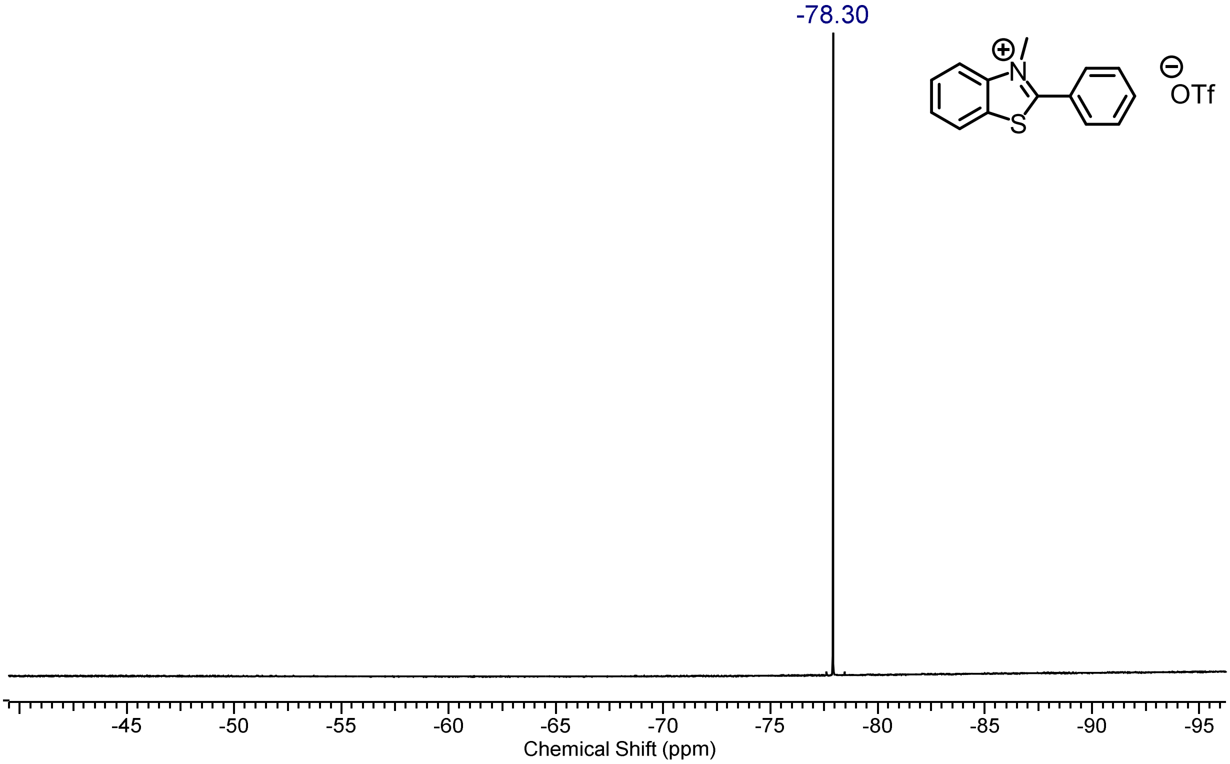

N-methyl-2-phenyl-benzothiazolium tetraphenylborate ([2][BPh_4])

^1H, $^{13}C\{^1H\}$ and ^{11}B -NMR spectra

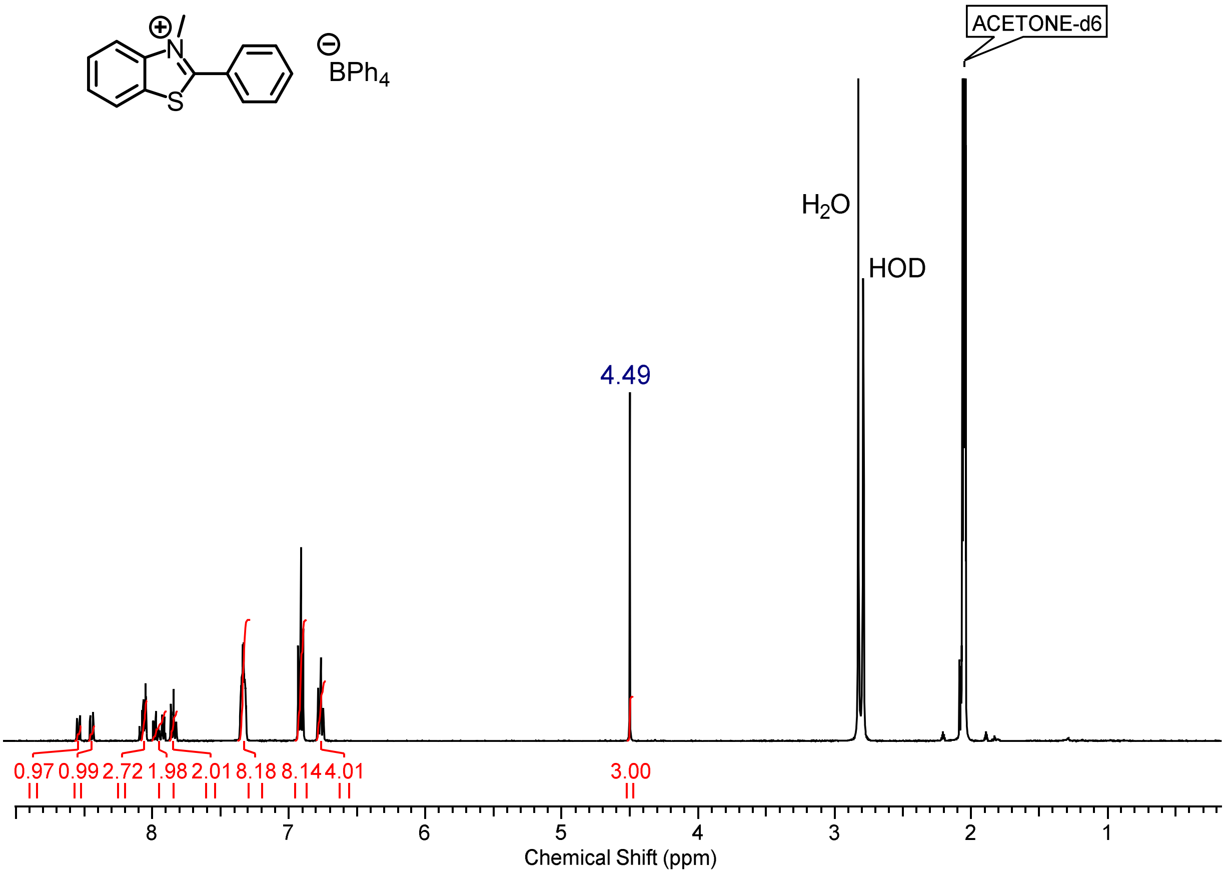

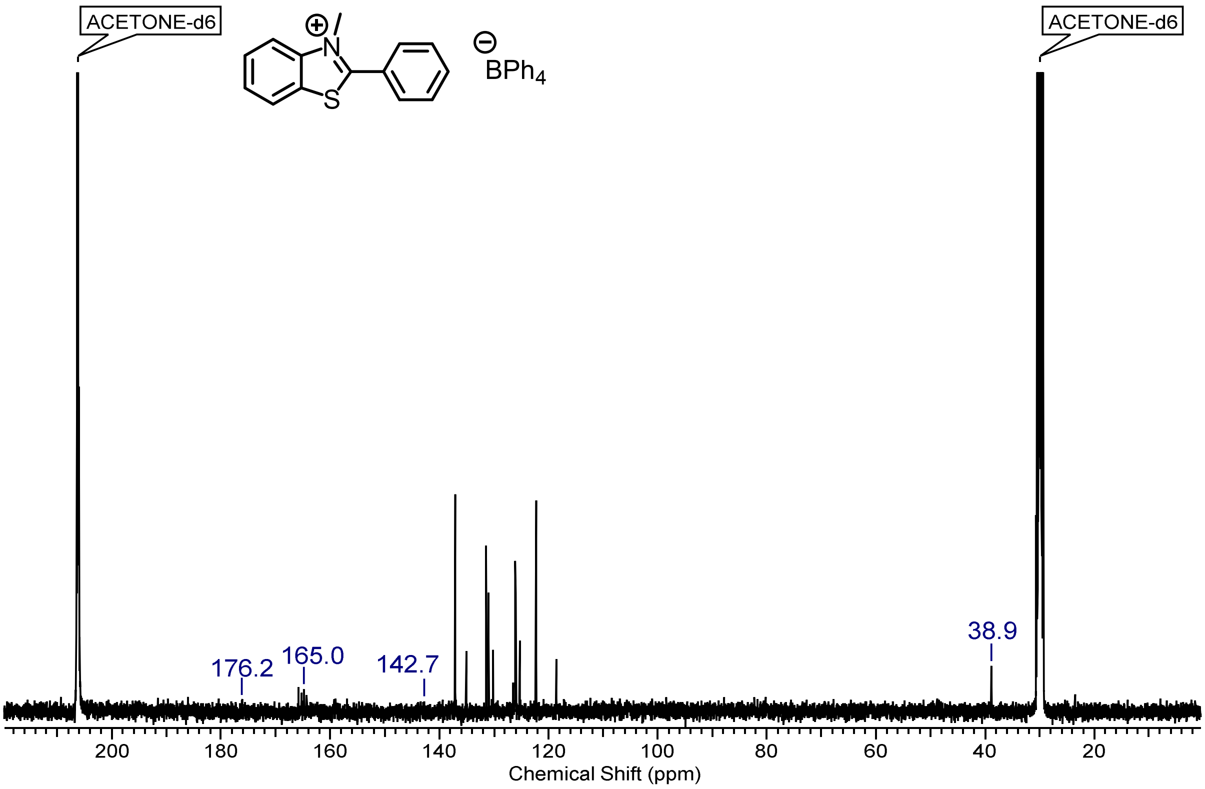
ACETONE-d6
N
S
BPh4
ACETONE-d6
176.2
165.0
142.7
38.9
200
180
160
140
120
100
80
60
40
20
Chemical Shift (ppm)

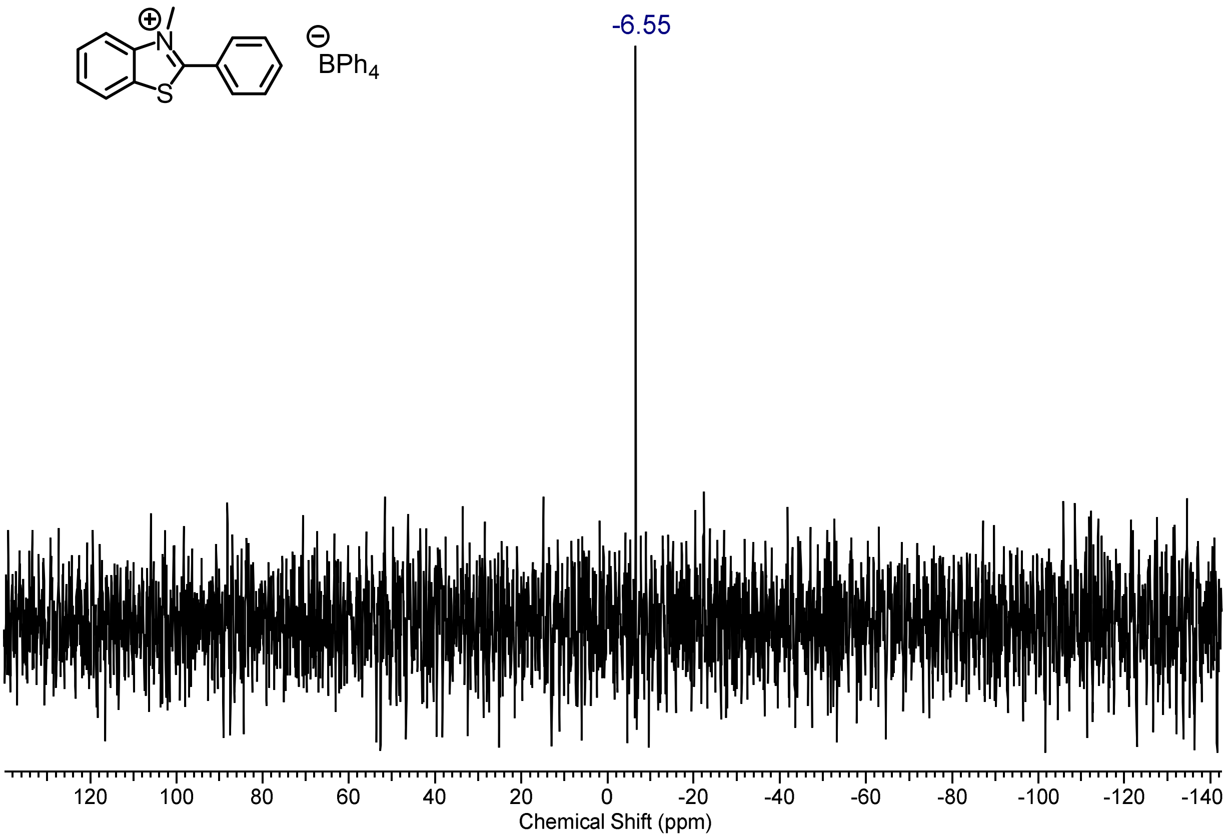
N
S
BPh4
-6.55
120
100
80
60
40
20
0
-20
-40
-60
-80
-100
-120
-140
Chemical Shift (ppm)

***N*-methyl-2-phenyl-benzothiazolium tetra(3,5-dichlorophenyl)borate ([2][BArCl])**
^{1}H, $^{13}C\{^{1}H\}$ and ^{11}B -NMR spectra

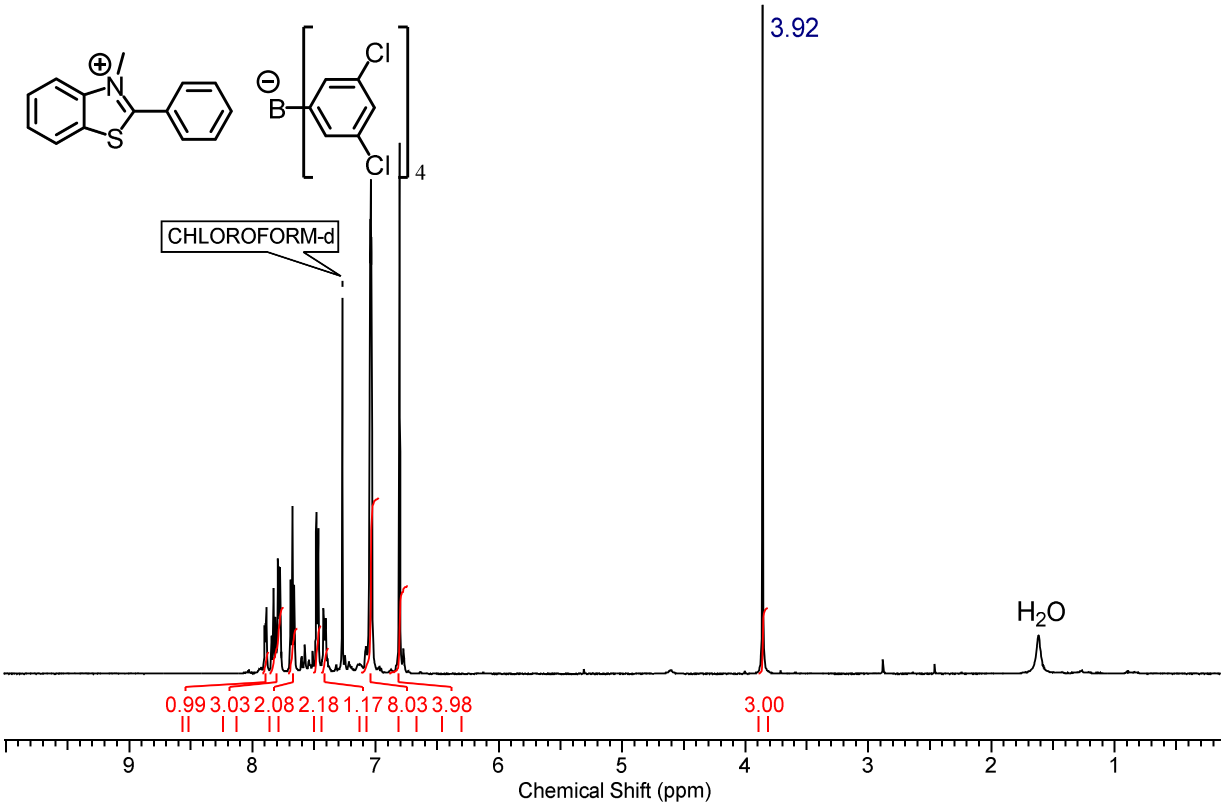

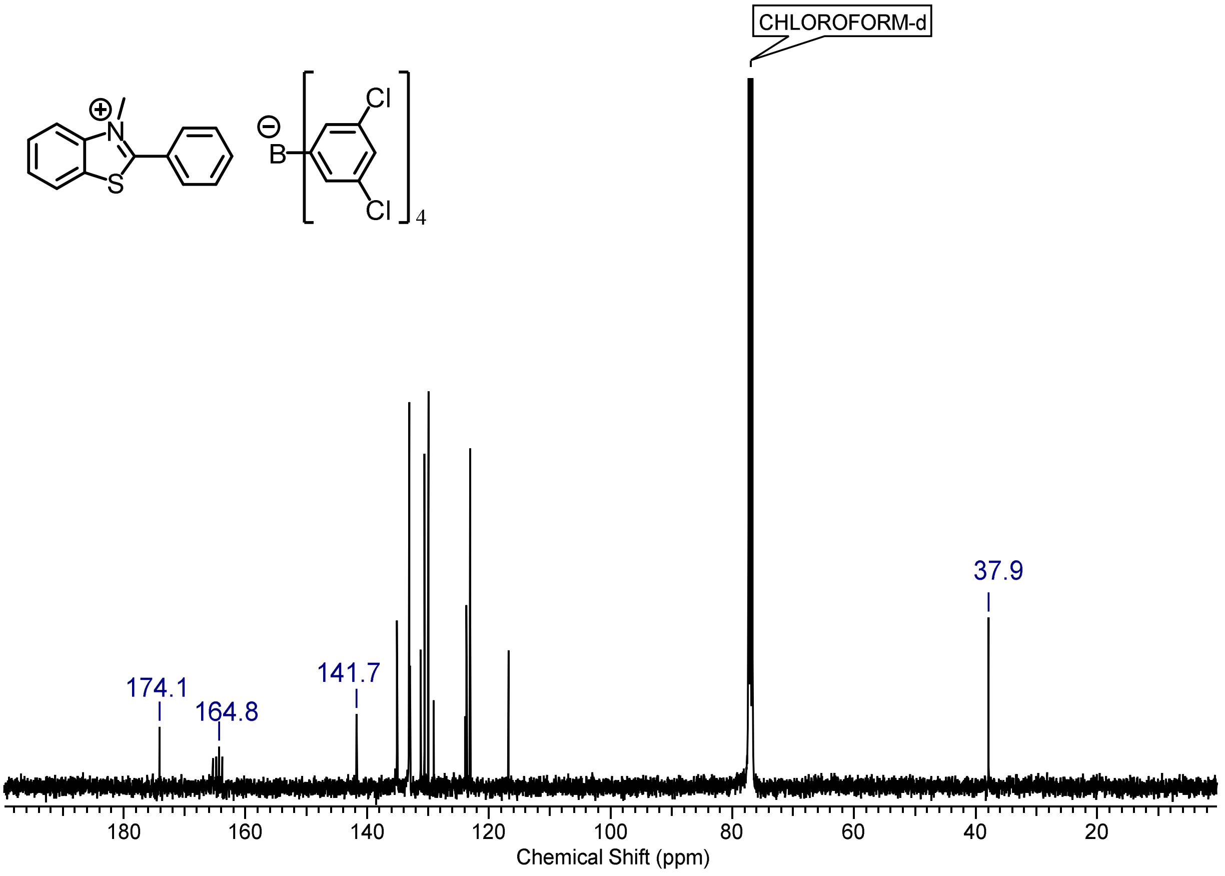

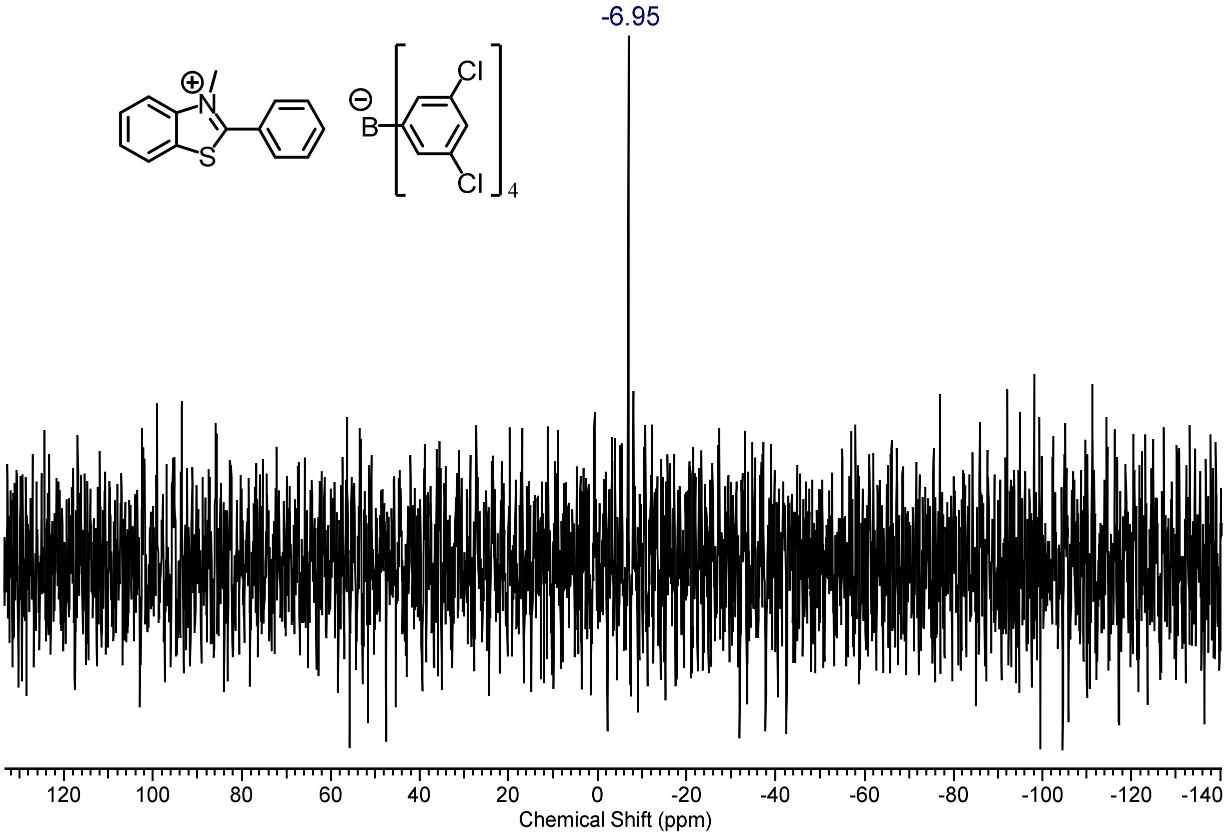

_N_-methyl-2-(1-naphthyl)-benzothiazolium iodide ([5][I])
^{1}H and ^{13}C{^{1}H}-NMR spectra

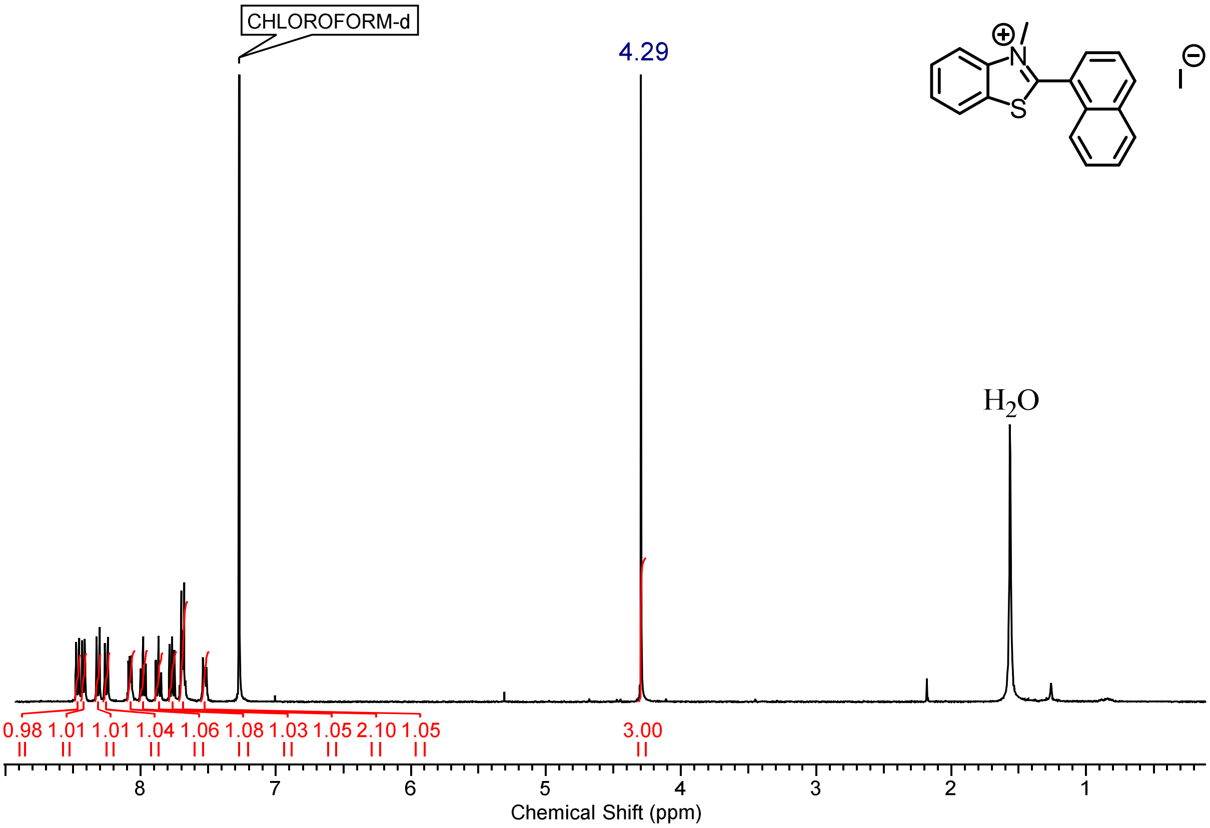

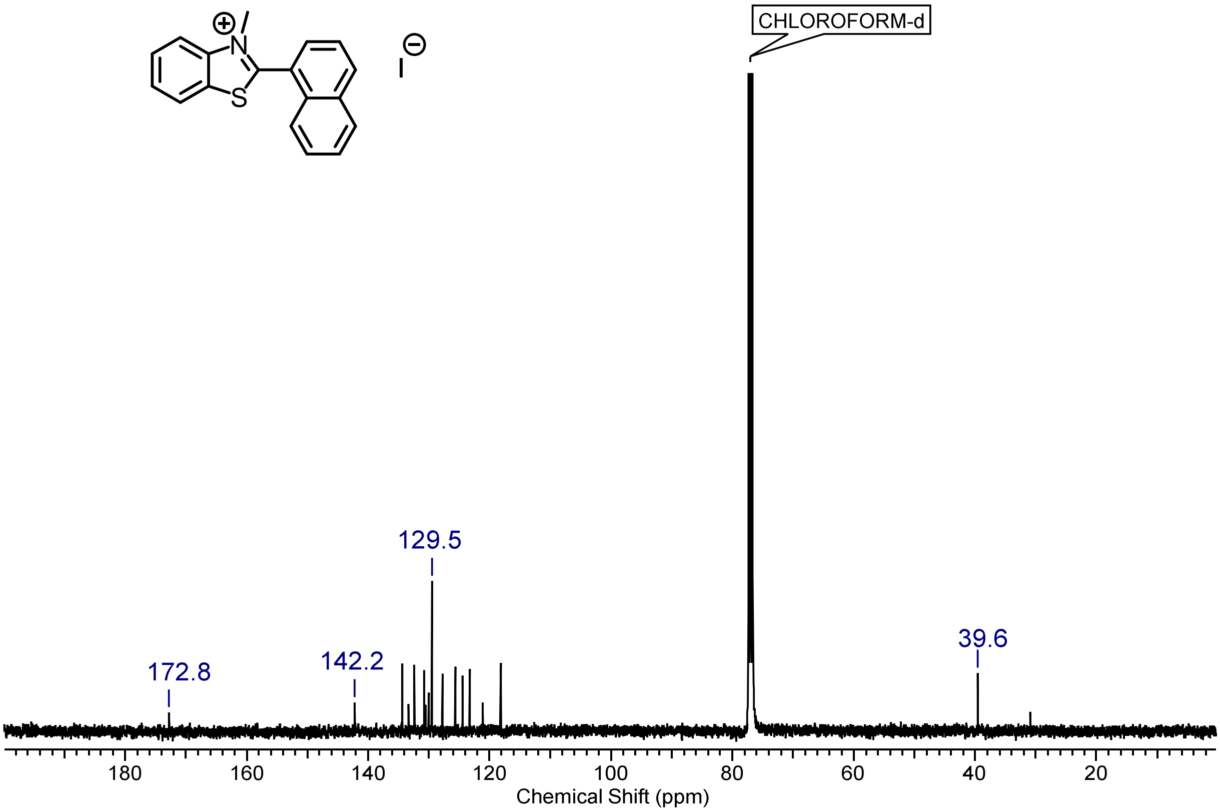

_N_-methyl-2-(1-naphthyl)-benzothiazolium tetraphenylborate ([5][BPh_4])
^{1}H, ^{13}C and ^{11}B-NMR spectra

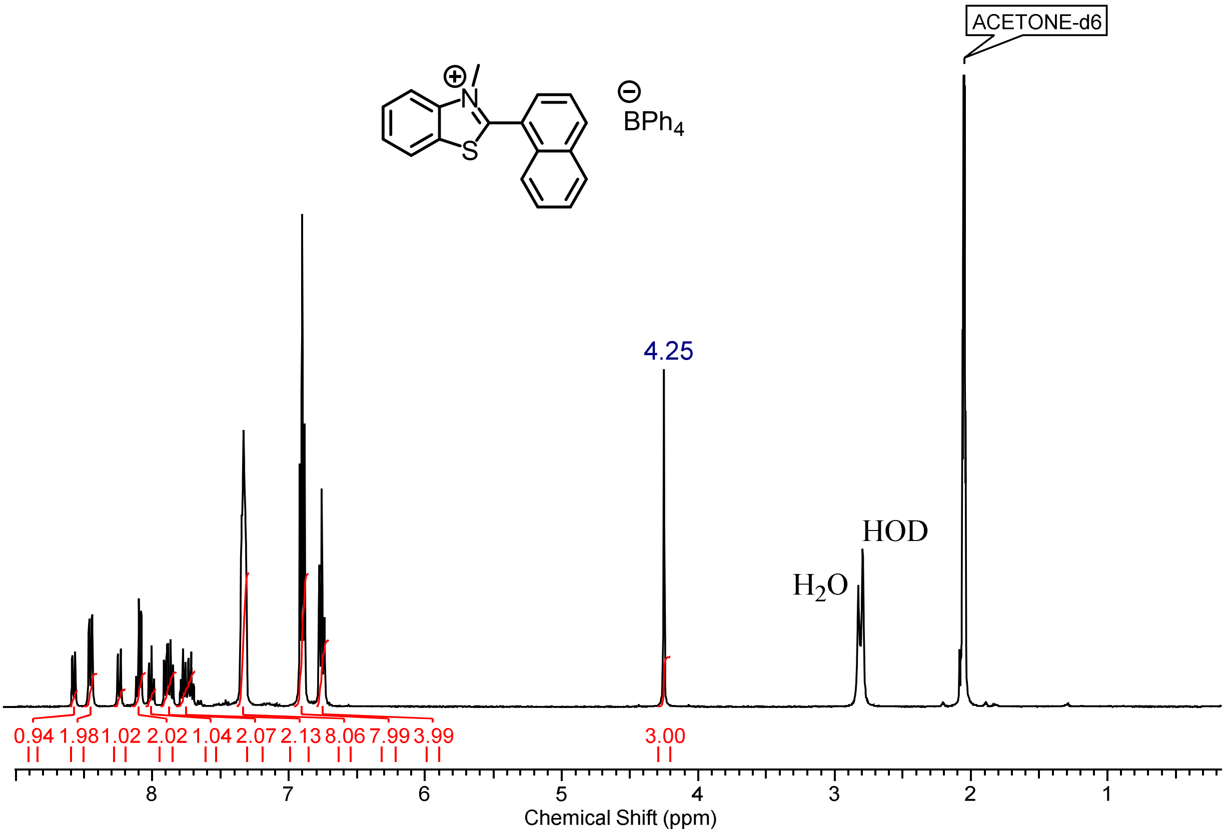

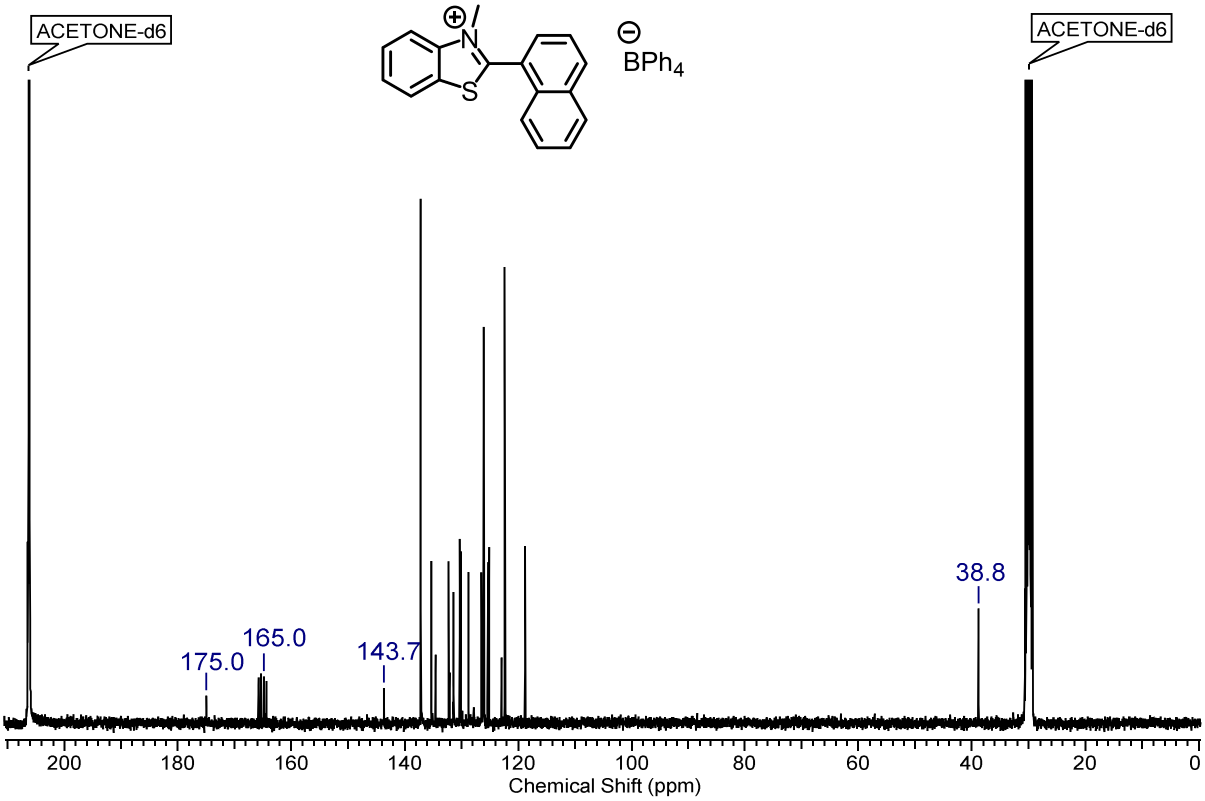
ACETONE-d6
BPh4
ACETONE-d6
38.8
165.0
175.0
143.7
200
180
160
140
120
100
80
60
40
20
0
Chemical Shift (ppm)

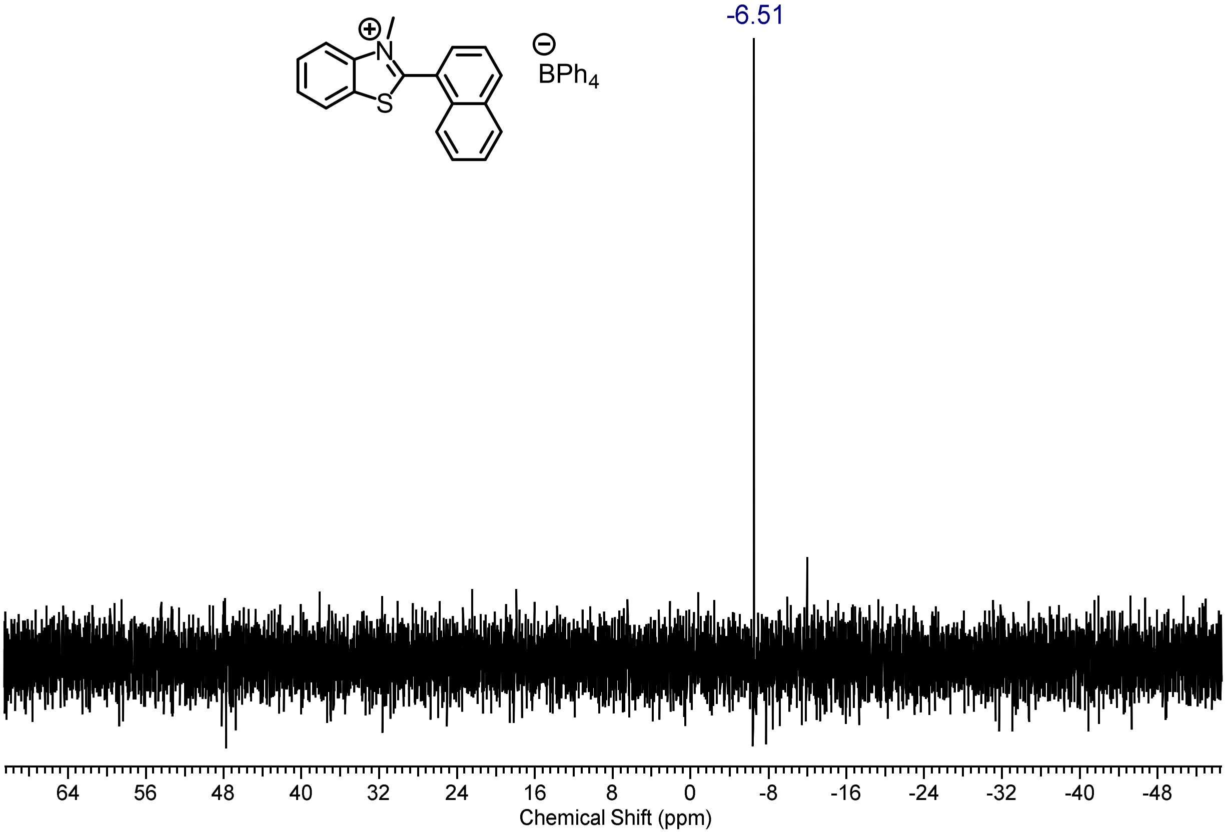
-6.51
BPh4
64
56
48
40
32
24
16
8
0
-8
-16
-24
-32
-40
-48
Chemical Shift (ppm)

_N_-methyl-2-(1-naphthyl)-benzothiazolium tetra(3,5-dichlorophenyl)borate ([5][BArCl])
^{1}H, ^{13}C{^{1}H} and ^{11}B-NMR spectra

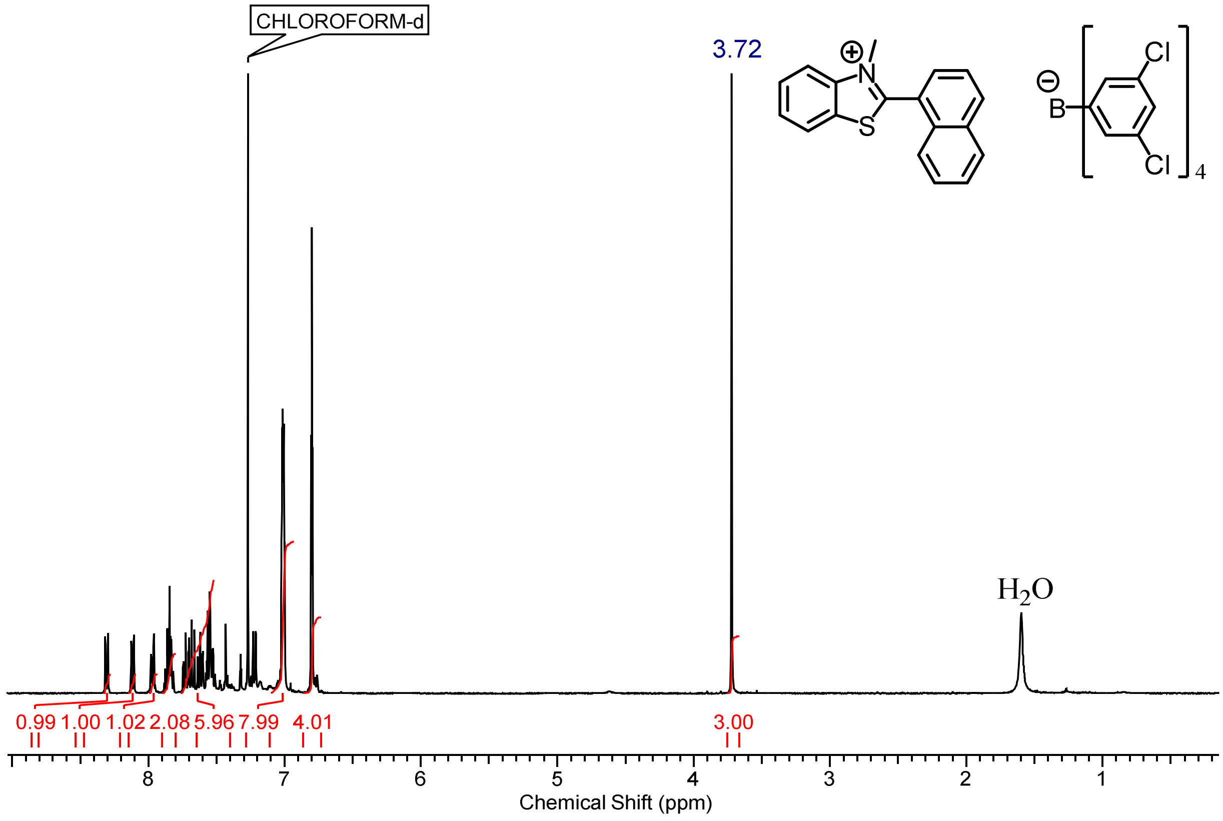

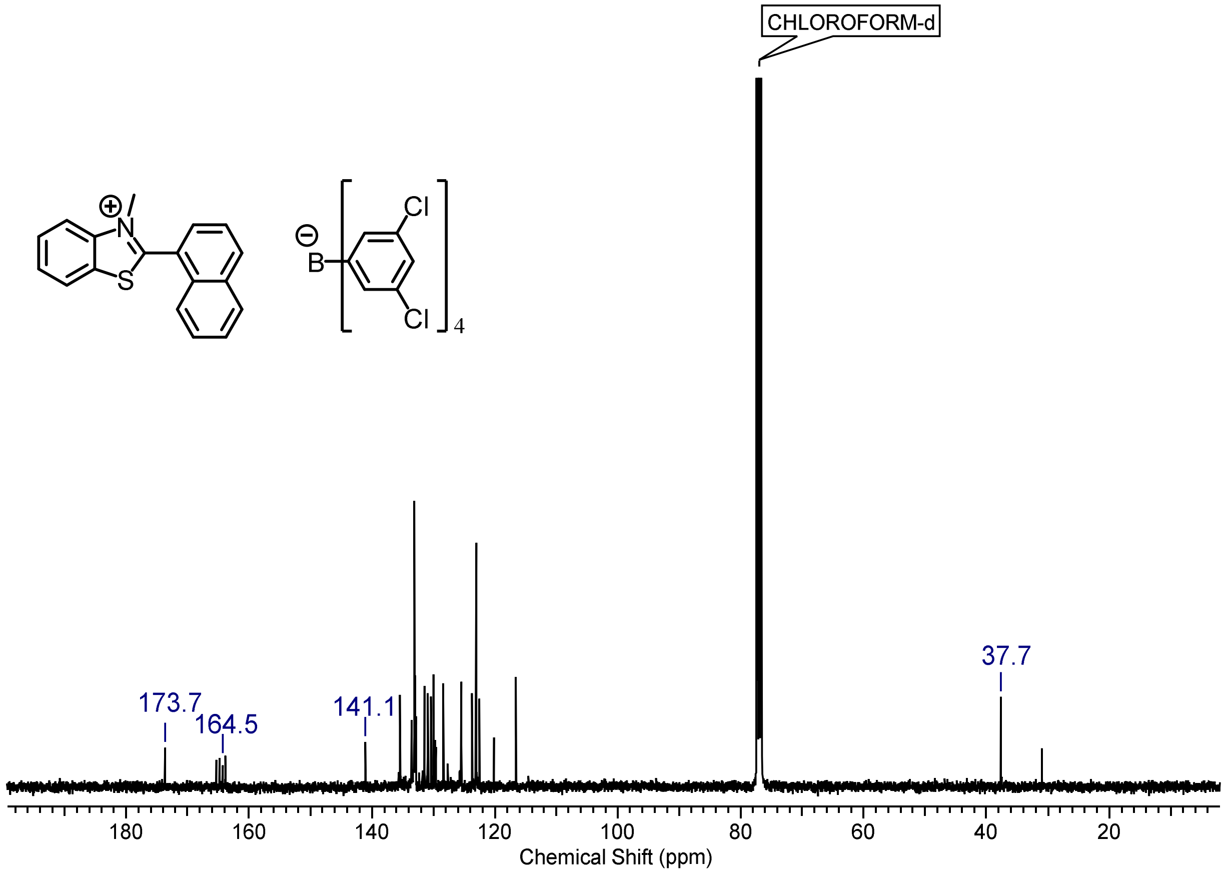

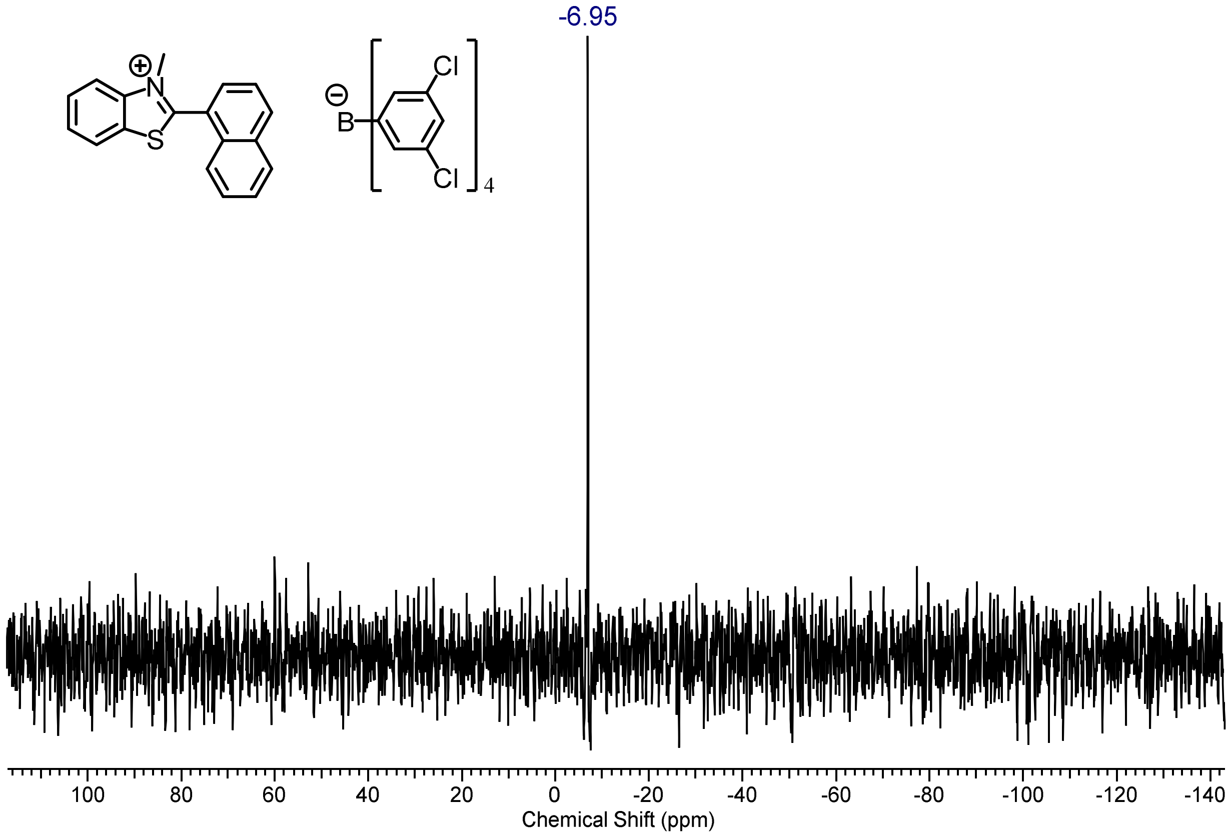

_N_-methyl-2-(4-_t_-butylphenyl)-benzothiazolium iodide ([4][I])

^{1}H and ^{13}C{^{1}H}-NMR spectra

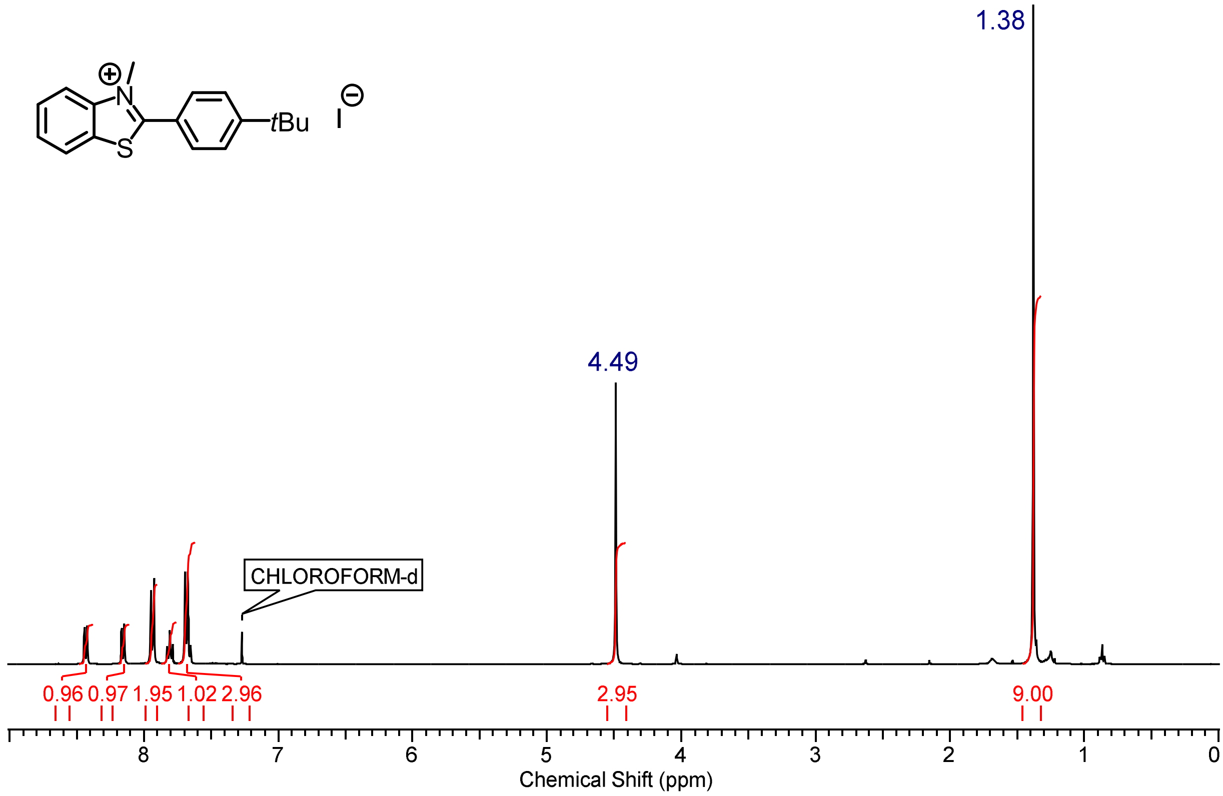

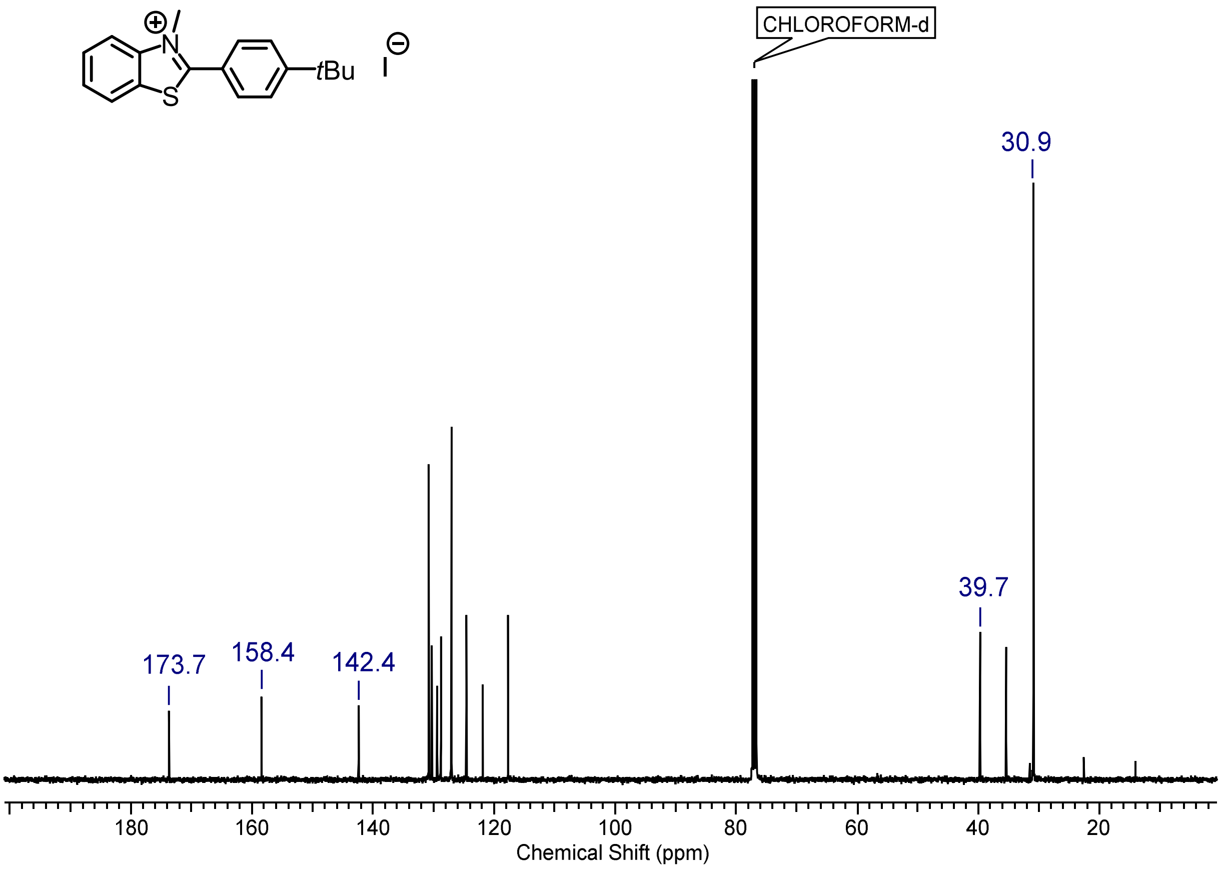

N-methyl-2-(4-t-butylphenyl)-benzothiazolium tetra(3,5-dichlorophenyl)borate ([4][BArCl])

^{1}H, ^{13}C{^{1}H} and ^{11}B-NMR spectra

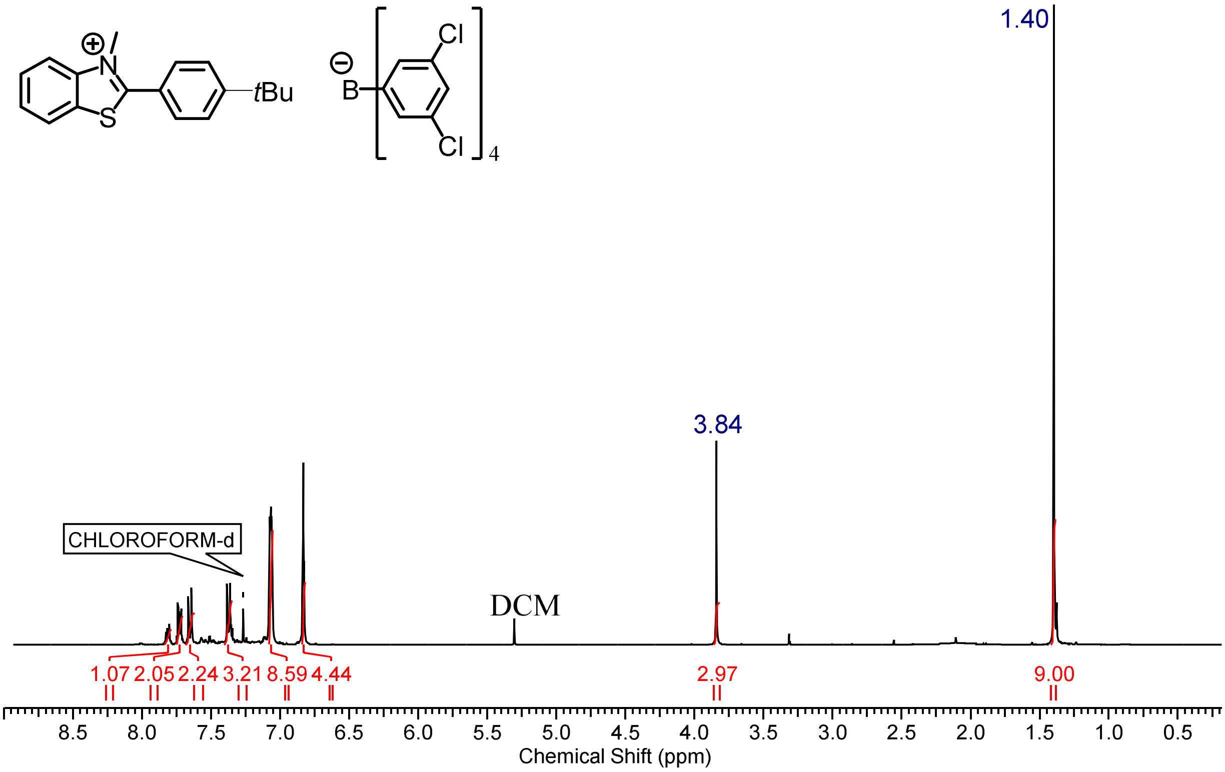

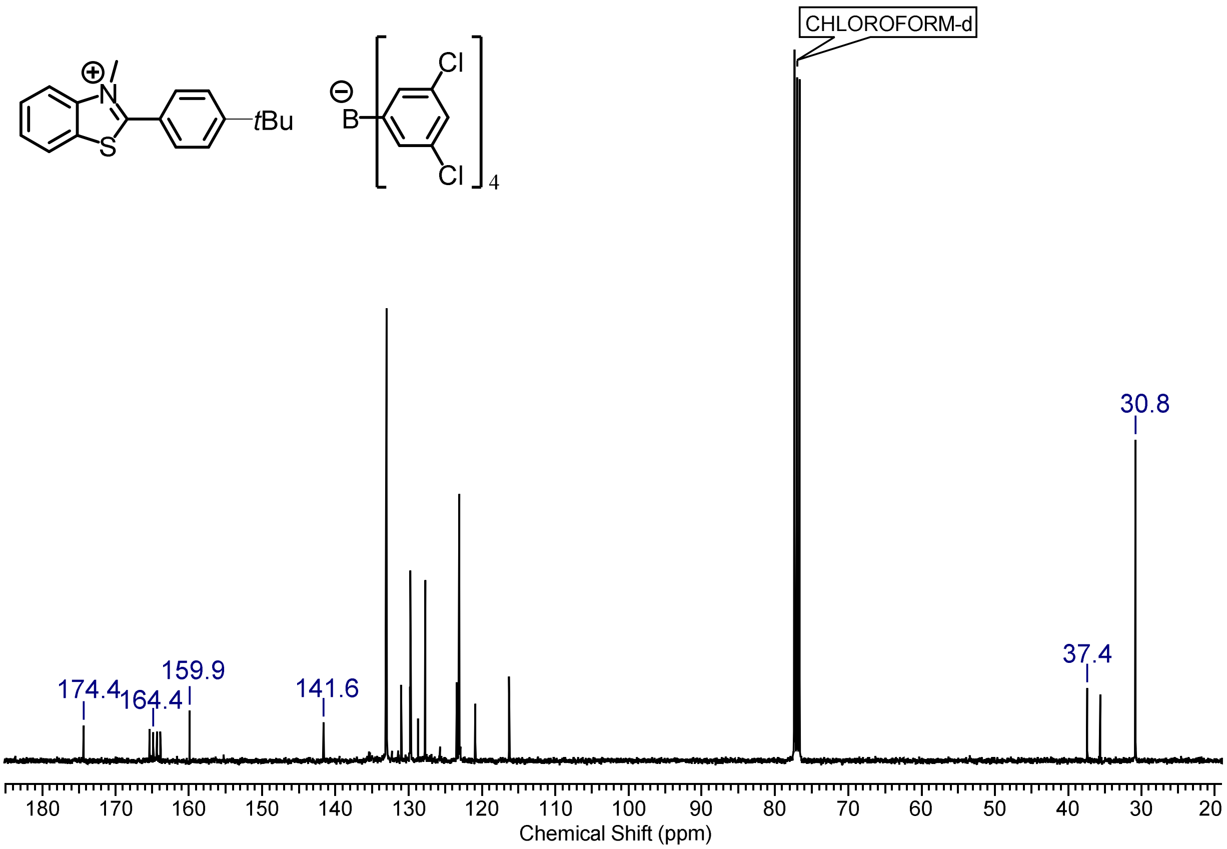
tBu
B
Cl
Cl
4
CHLOROFORM-d
30.8
37.4
174.4
164.4
159.9
141.6
180 170 160 150 140 130 120 110 100 90 80 70 60 50 40 30 20
Chemical Shift (ppm)

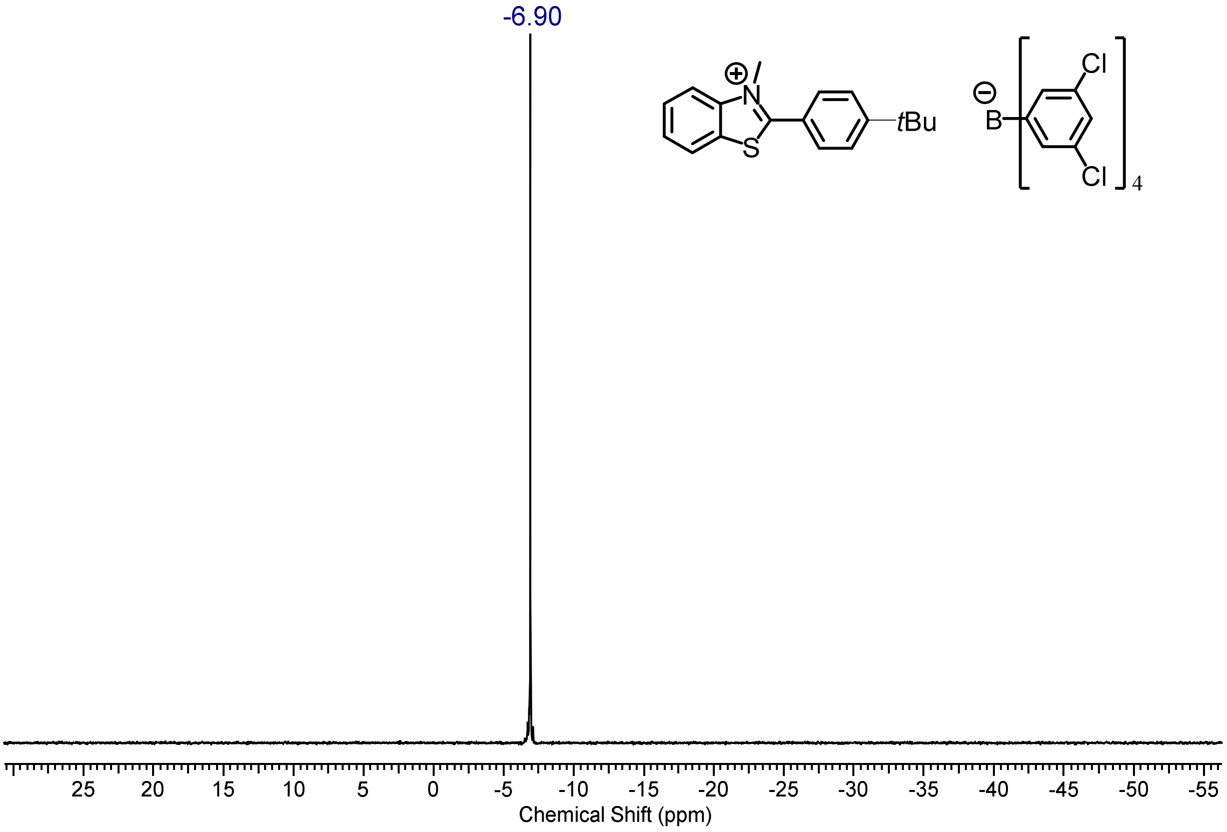
-6.90
tBu
B
Cl
Cl
4
25 20 15 10 5 0 -5 -10 -15 -20 -25 -30 -35 -40 -45 -50 -55
Chemical Shift (ppm)

N-methyl-2-(4-methoxyphenyl)-benzothiazolium iodide ([6][I])
^{1}H and $^{13}C\{^{1}H\}$-NMR spectra

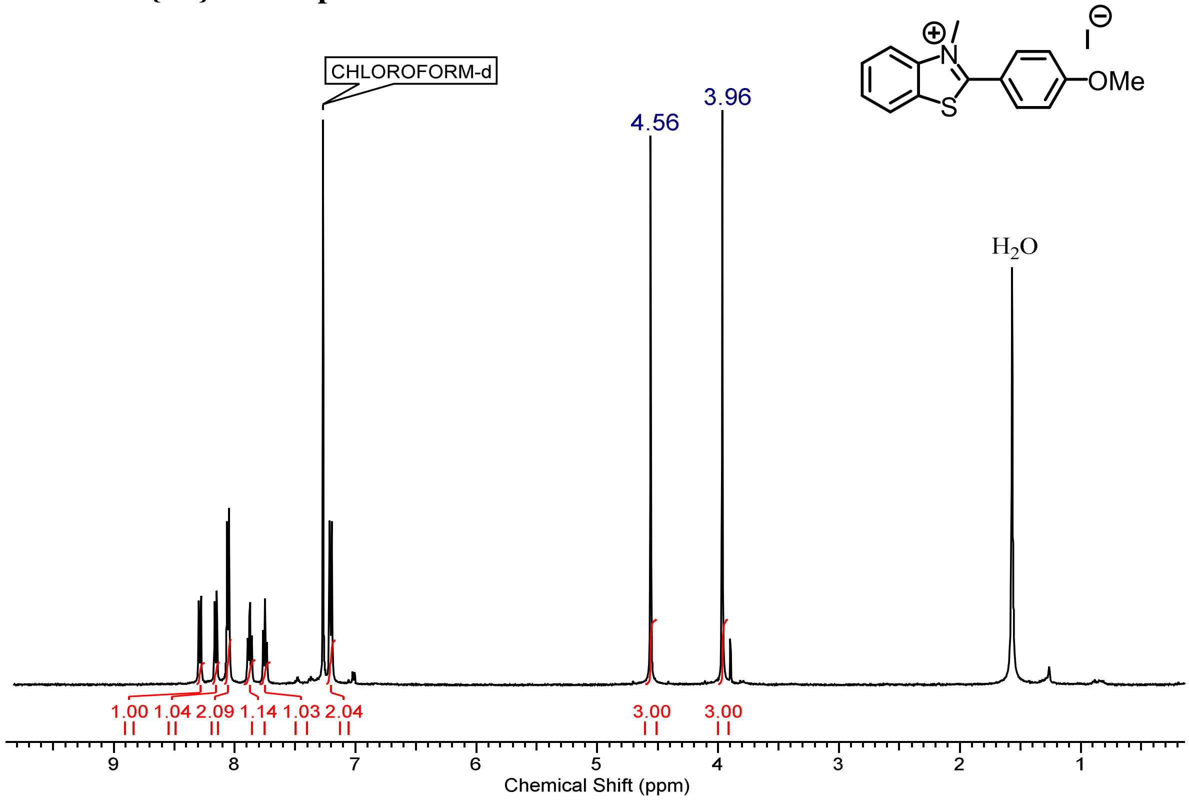

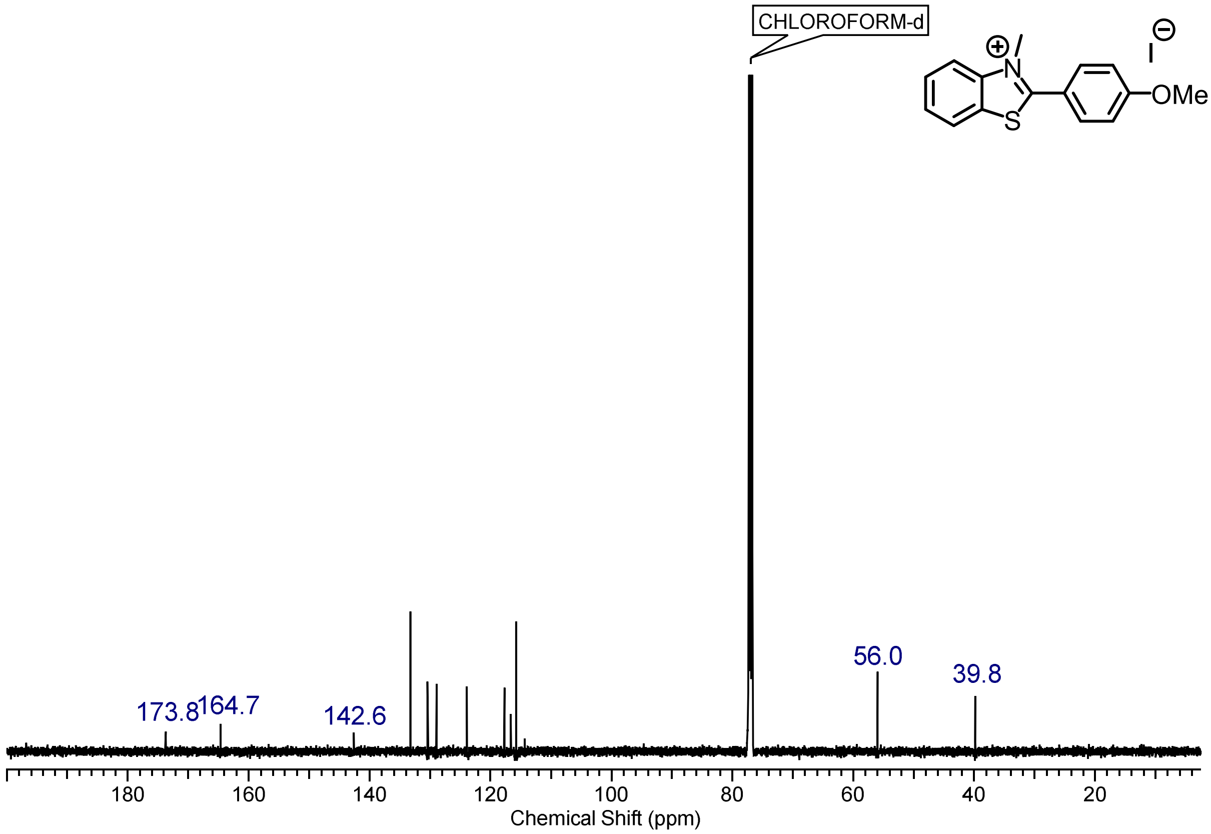

N-methyl-2-(4-methoxyphenyl)-benzothiazolium tetra(3,5-dichlorophenyl)borate ([6][BArCl])

^{1}H and ^{13}C{^{1}H} and ^{11}B -NMR spectra

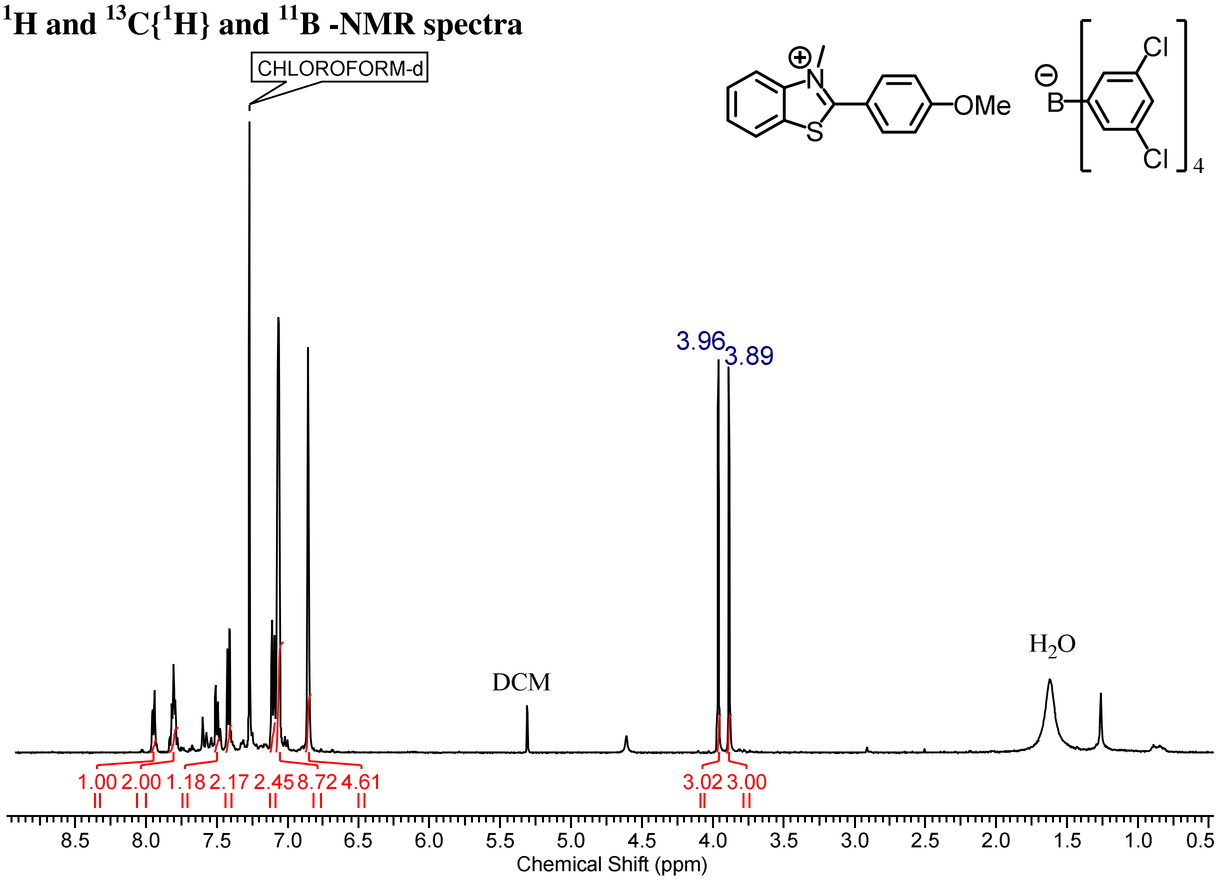

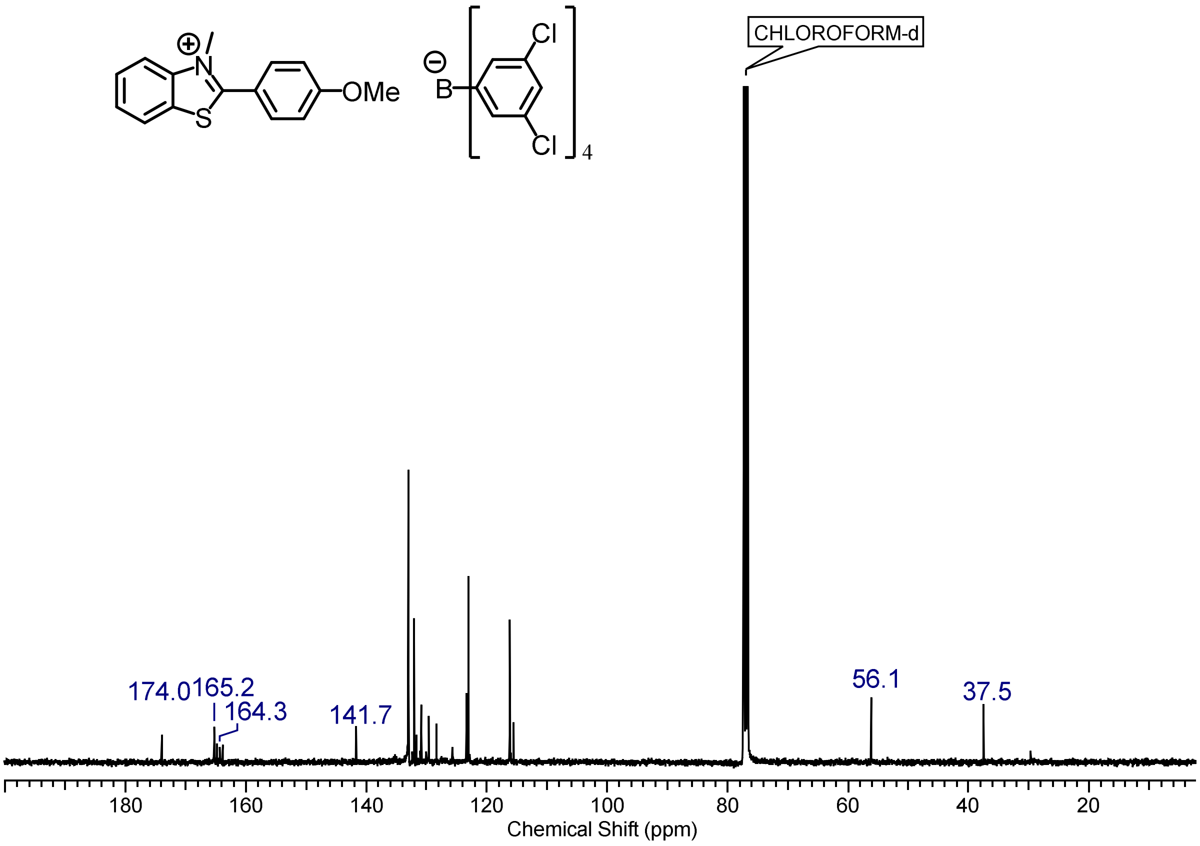

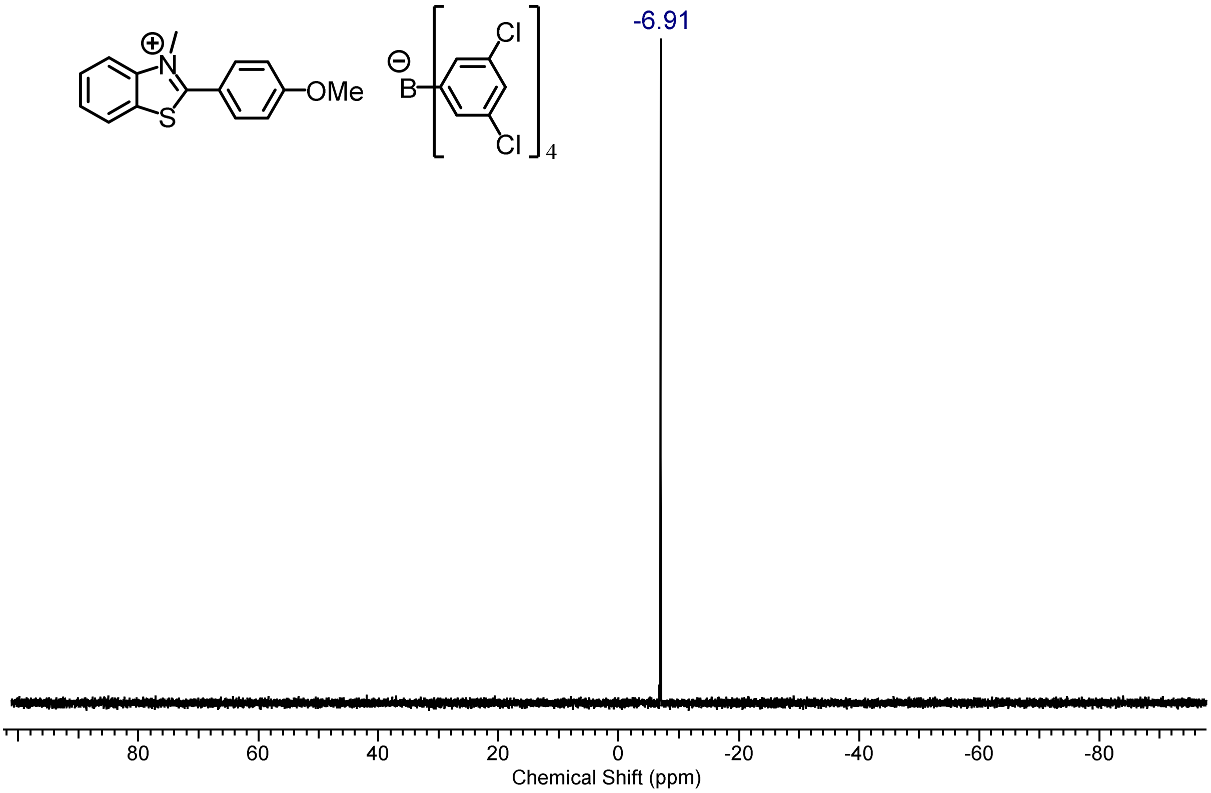

***N*-methyl-2-phenyl-benzoxazolium iodide ([7][I])**
^{1}H and ^{13}C{^{1}H}-NMR spectra

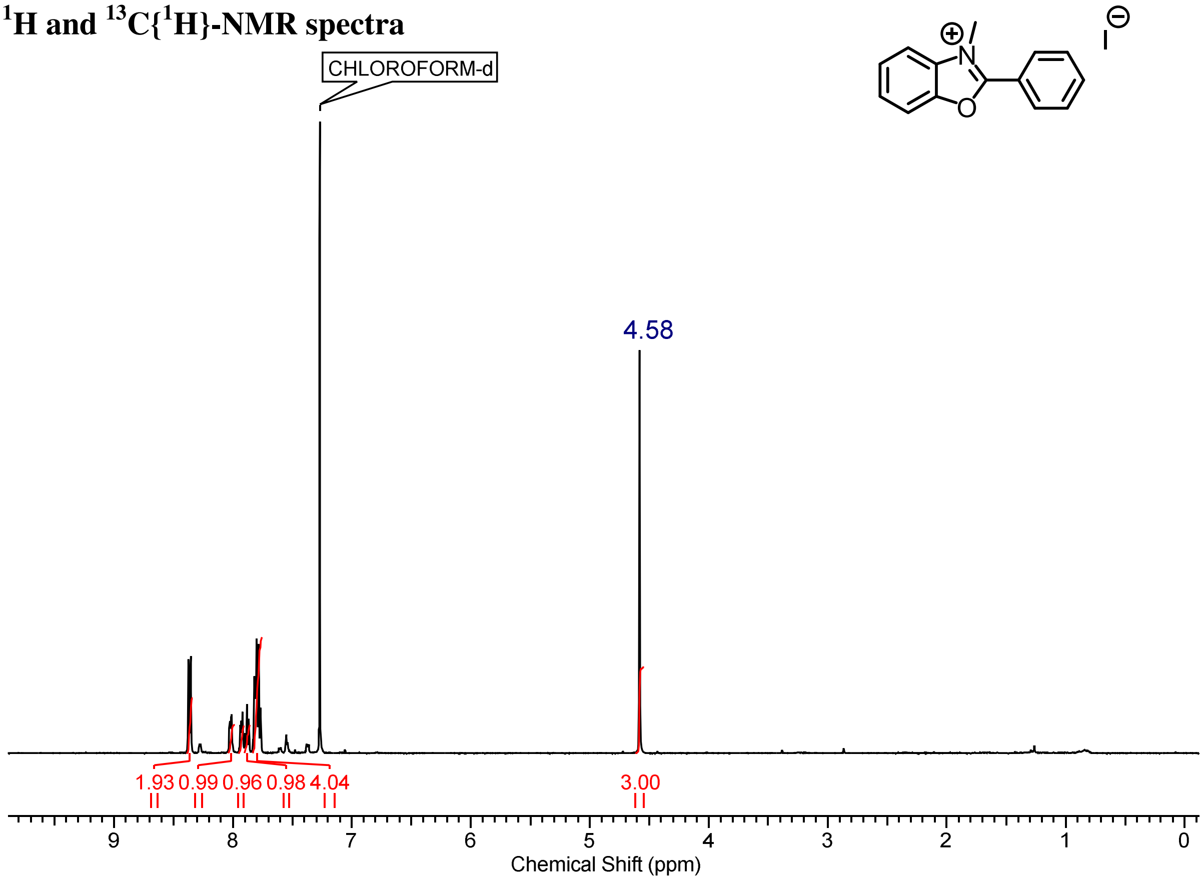

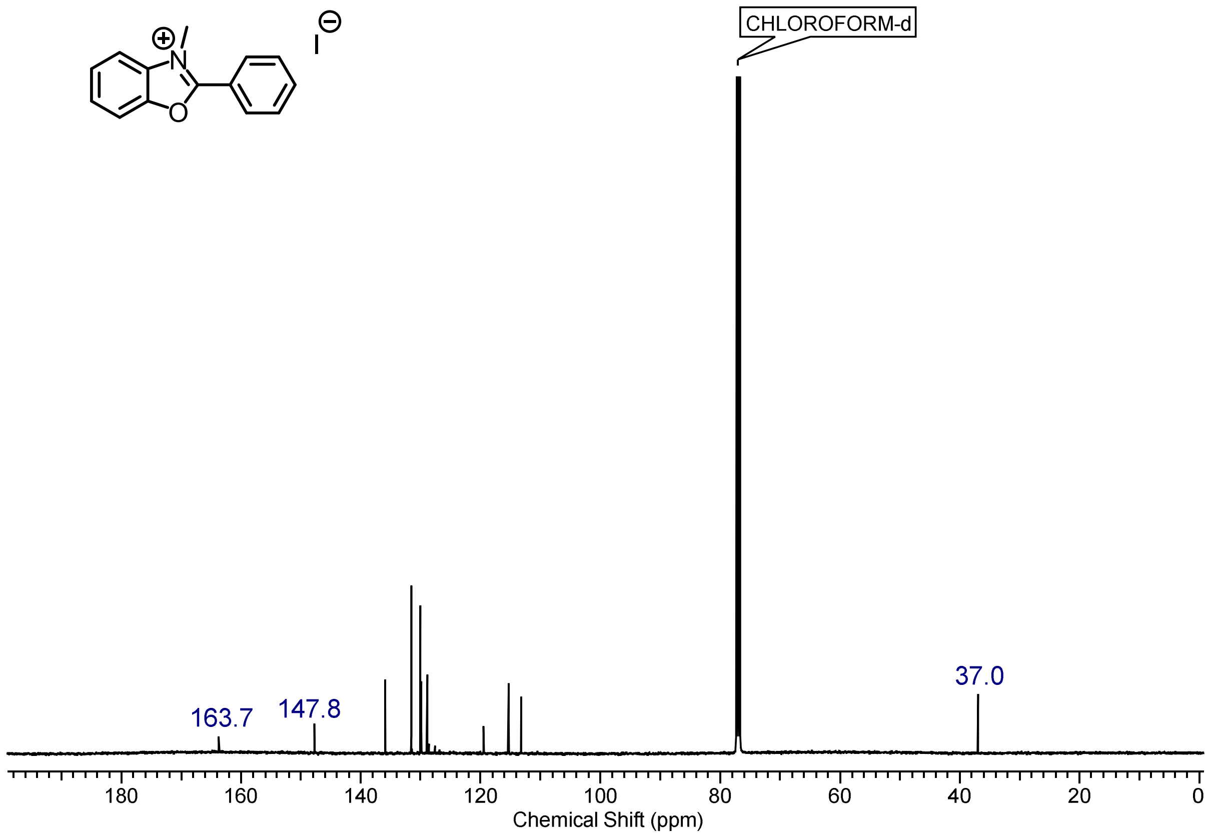

_N_-methyl-2-phenyl-benzoxazolium tetra(3,5-dichlorophenyl)borate ([7][BArCl])
^{1}H and ^{13}C{^{1}H} and ^{11}B -NMR spectra

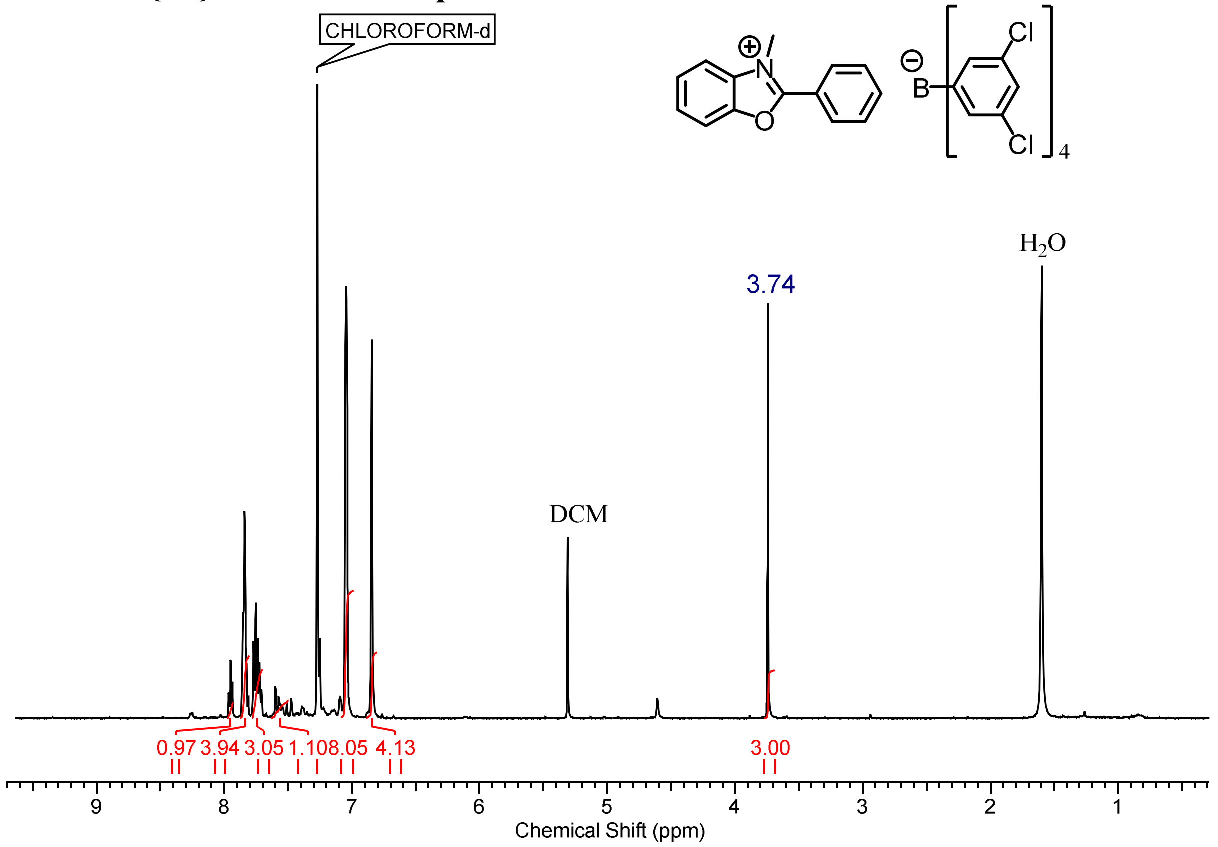

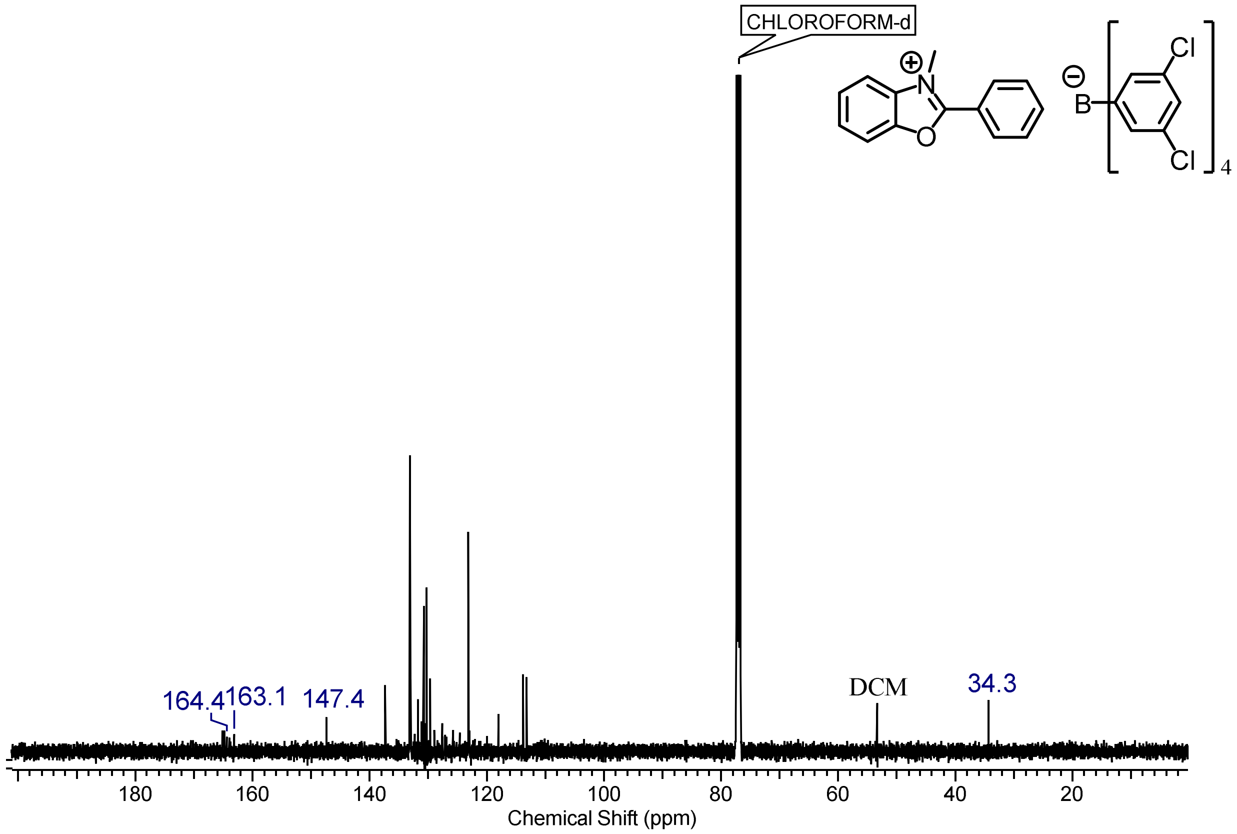
CHLOROFORM-d
Cl
B
Cl
N
O
4
164.4
163.1
147.4
DCM
34.3
180
160
140
120
100
80
60
40
20
Chemical Shift (ppm)

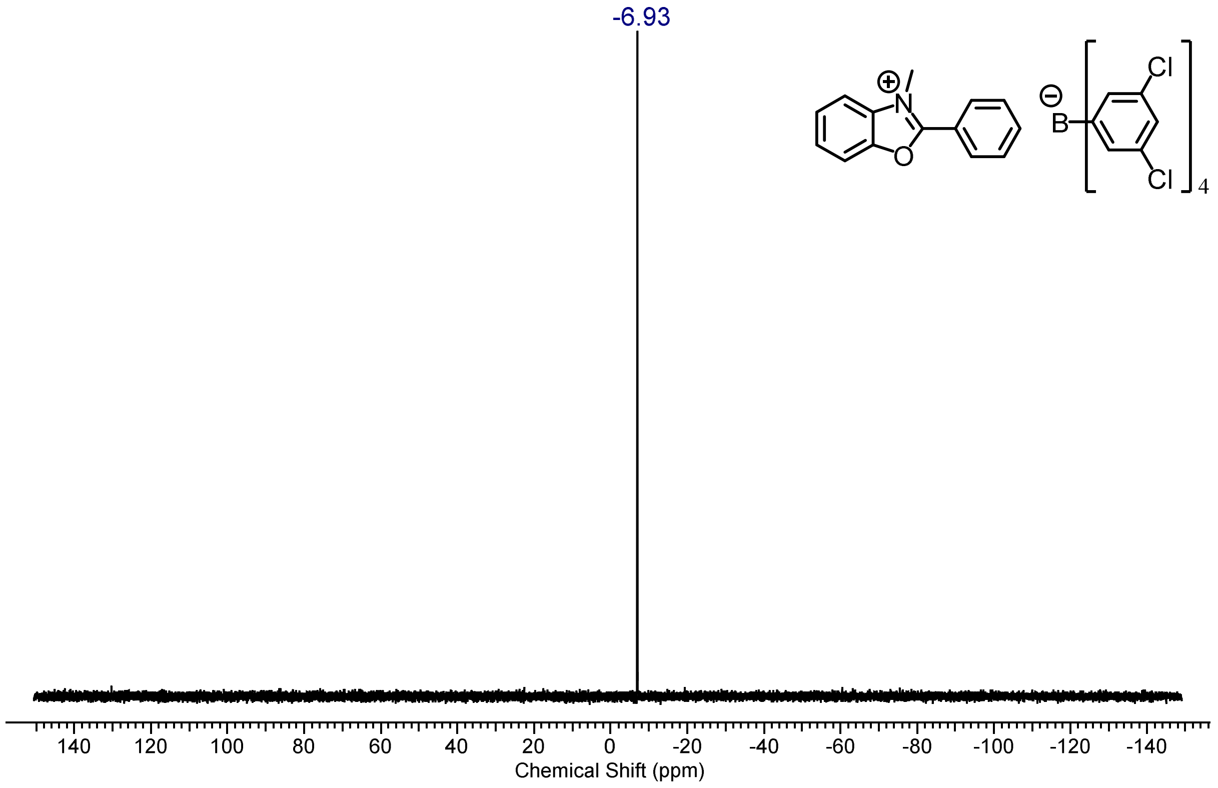
-6.93
Cl
B
Cl
N
O
4
140
120
100
80
60
40
20
0
-20
-40
-60
-80
-100
-120
-140
Chemical Shift (ppm)

***N,N*-dimethyl-2-phenyl-benzimidazolium iodide ([8][I])**
^{1}H and $^{13}C\{^{1}H\}$-NMR spectra

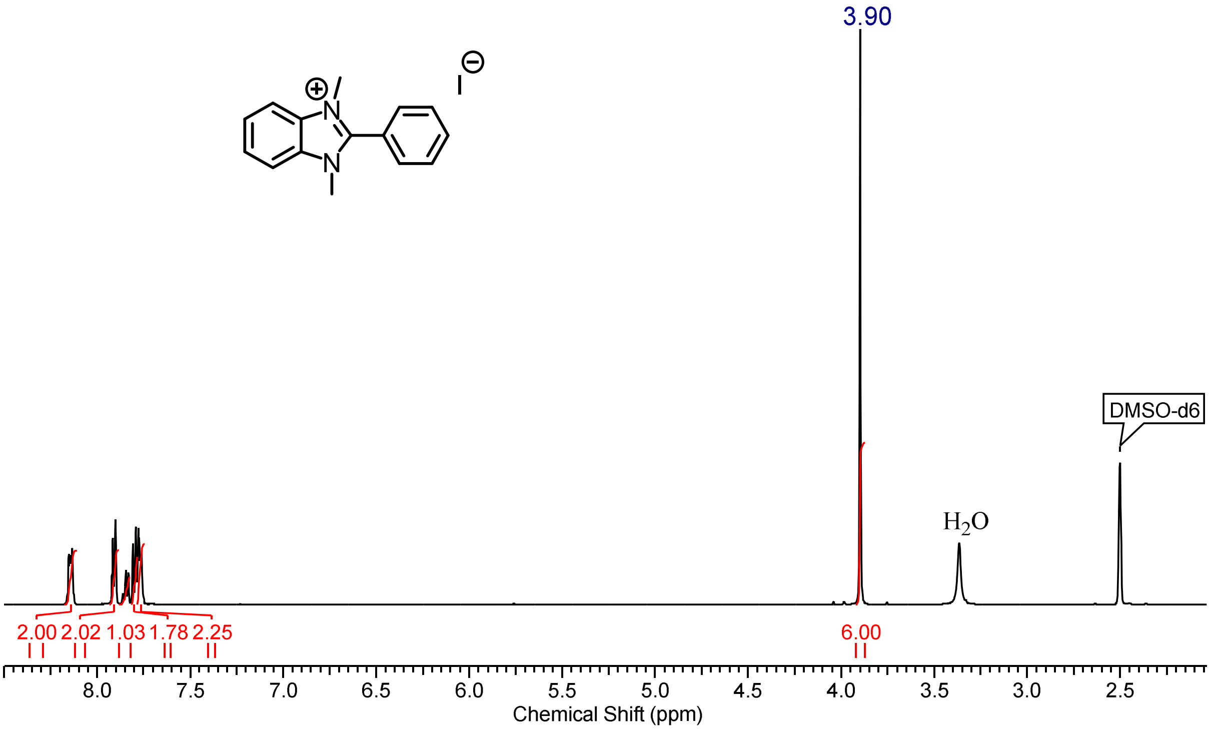

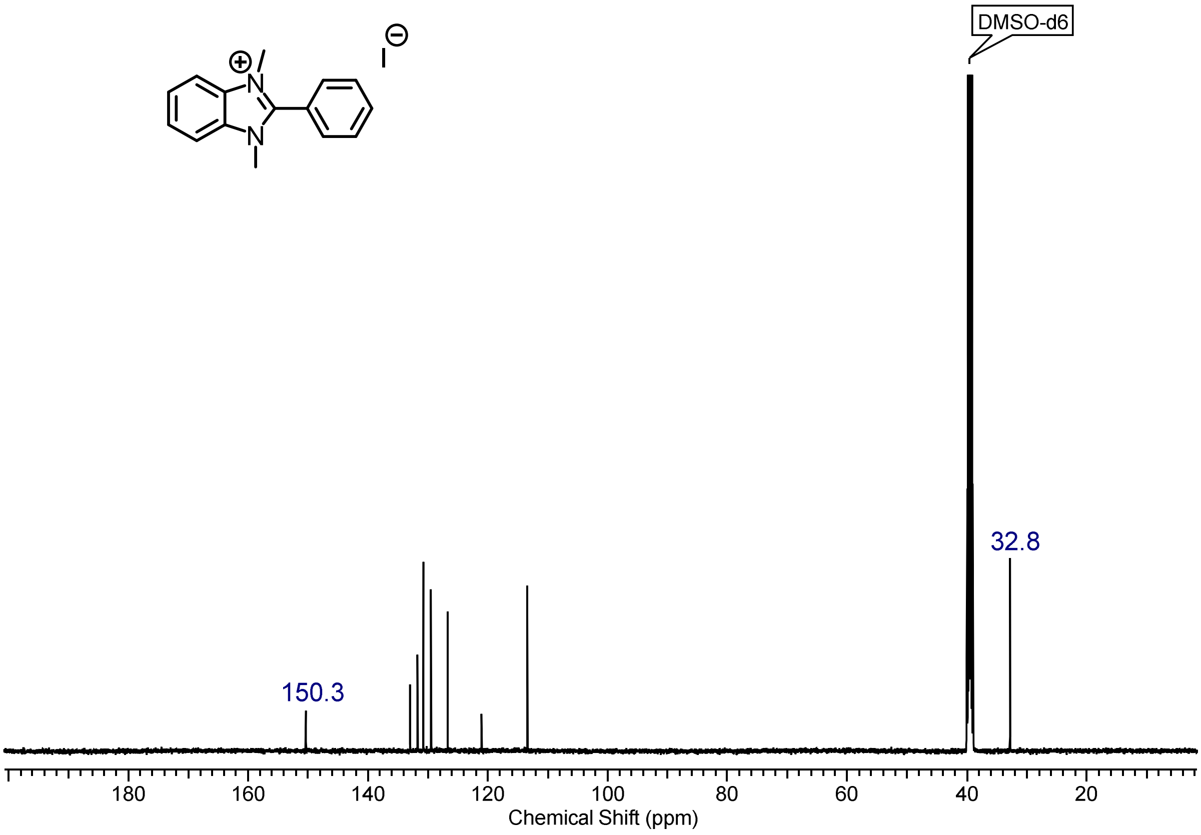

***N,N*-dimethyl-2-phenyl-benzimidazolium tetra(3,5-dichlorophenyl)borate ([8][BArCl])**
^{1}H and ^{13}C{^{1}H} and ^{11}B -NMR spectra

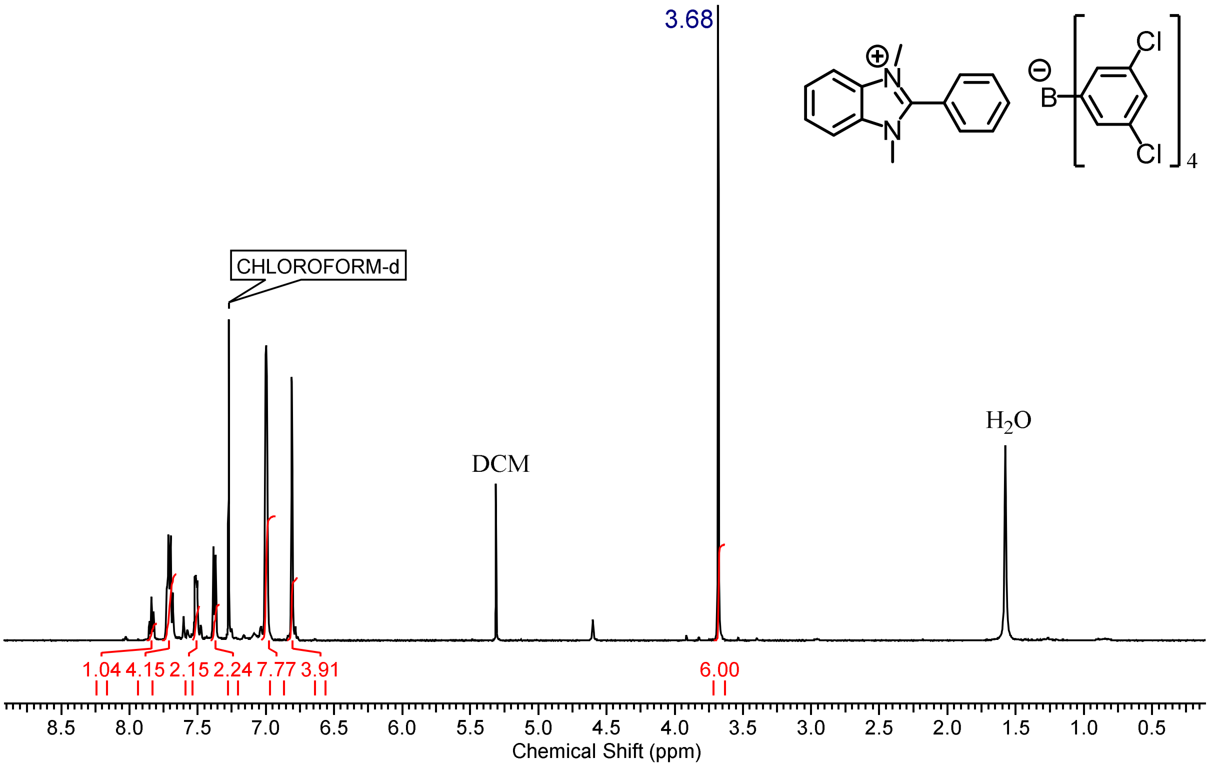

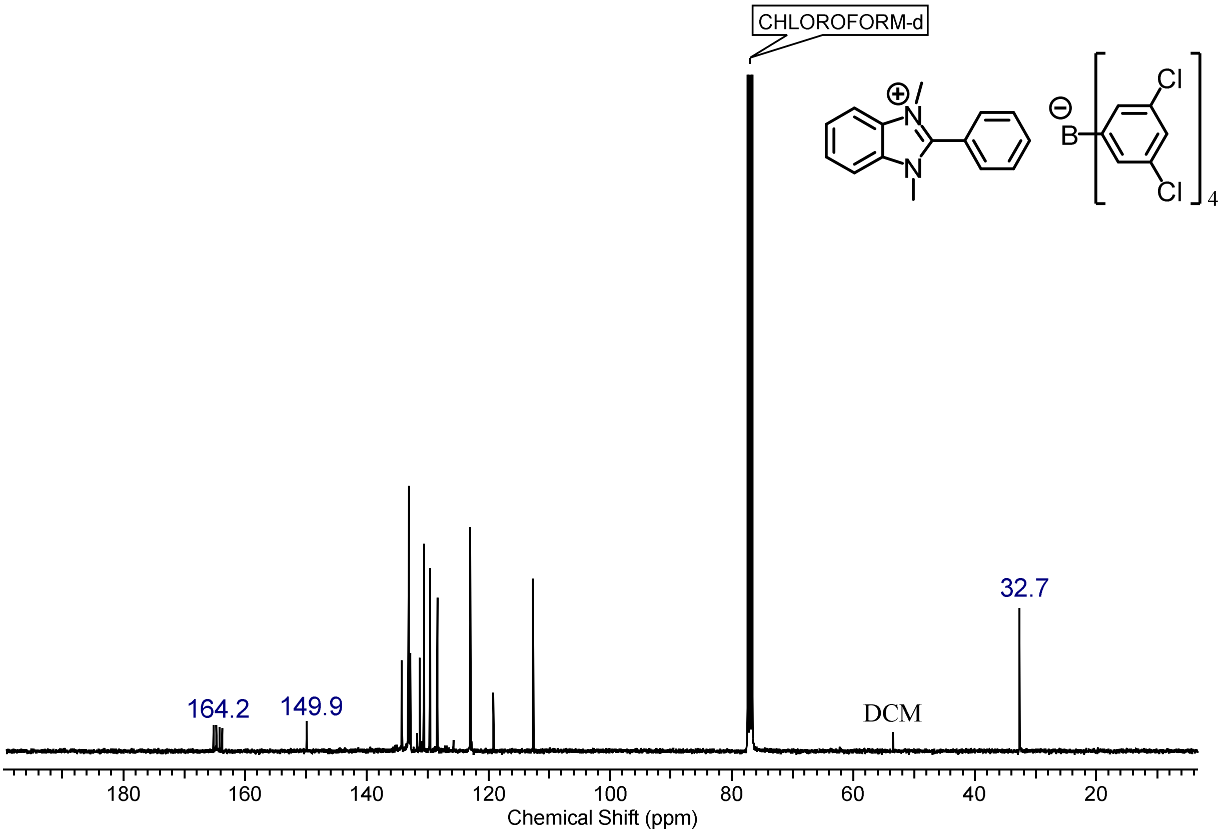

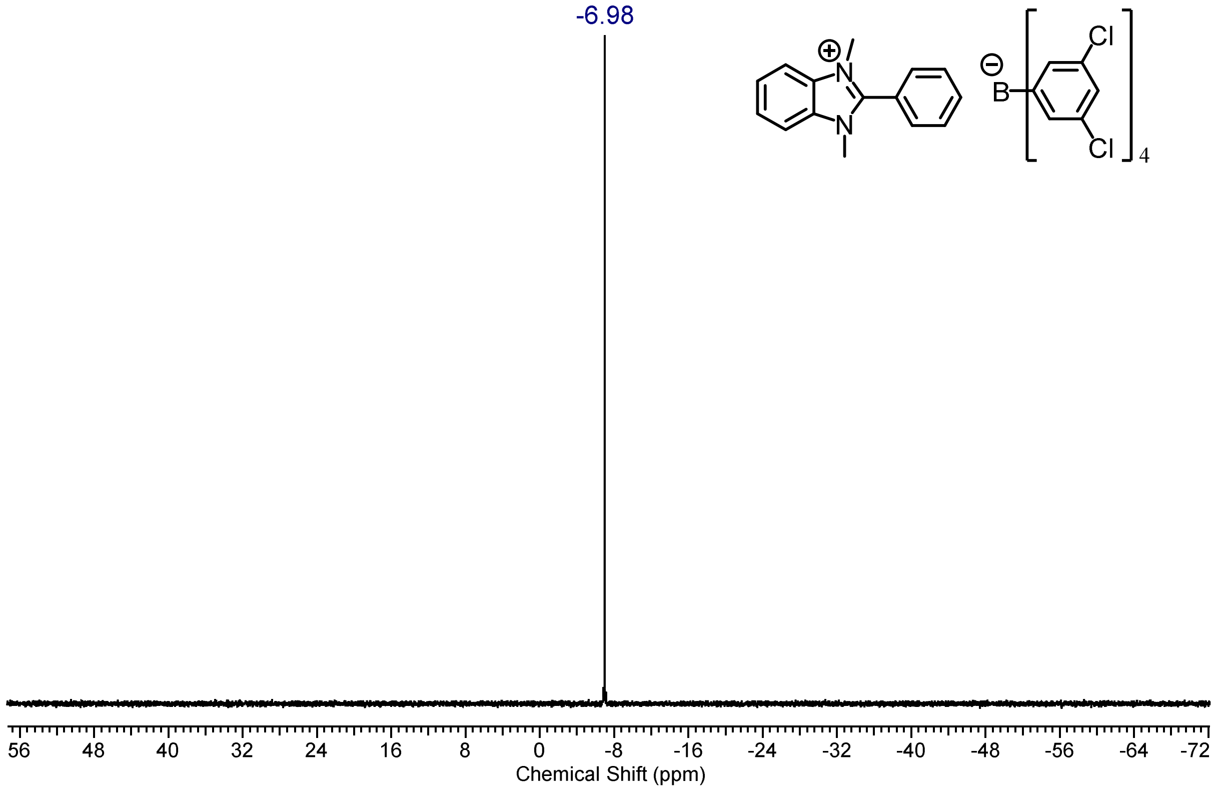
-6.98
N
N
B
Cl
Cl
4
56
48
40
32
24
16
8
0
-8
-16
-24
-32
-40
-48
-56
-64
-72
Chemical Shift (ppm)
